# Macrocyclic Covalent Encapsulation of a Multi-Resonant Emitter: Understanding and Controlling Interactions in Highly Efficient Deep-Blue OLEDs

**DOI:** 10.1021/jacs.5c16290

**Published:** 2026-02-17

**Authors:** Erin M. Holdsworth, Hwan-Hee Cho, Andrew D. Bond, Stephanie Montanaro, Seung-Je Woo, Tianyu Huang, Jordan Shaikh, Fathy Hassan, Sebastian Gorgon, Víctor Riesgo-Gonzalez, Alexander J. Gillett, Daniel G. Congrave, Richard H. Friend, Hugo A. Bronstein

**Affiliations:** † Yusuf Hamied Department of Chemistry, 2152 University of Cambridge, Lensfield Rd, Cambridge CB2 1EW, United Kingdom; ‡ Cavendish Laboratory, University of Cambridge, JJ Thomson Ave, Cambridge CB3 0HE, United Kingdom; § Department of Materials Science and Engineering, Yonsei University, 50 Yonsei-ro, Seodaemun-gu, Seoul 03722, Republic of Korea; ∥ Department of Chemistry, 6396 University of Oxford, Mansfield Rd, Oxford OX1 3TA, United Kingdom; ⊥ Chemistry Department, Faculty of Science, Tanta University, Tanta, El Gharbia 31527, Egypt; # Department of Physics, Chemistry and Biology (IFM), Linköping University, 581 83 Linköping, Sweden

## Abstract

Multi-resonant thermally activated delayed fluorescence (MR-TADF) emitters have emerged as popular candidates for the development of blue organic light-emitting diodes (OLEDs), offering narrowband emission, high photoluminescence quantum yields (PLQYs), and the ability to upconvert dark triplet states to bright singlet states. However, their planar polycyclic structures promote detrimental intermolecular interactions in the solid-state which diminish the color purity and introduce nonradiative loss pathways. Furthermore, the intrinsic luminescence of many MR-TADF emitters fails to satisfy the stringent color purity standards required for next-generation display technologies. Here, we synthetically address these issues by covalently encapsulating a blue-shifted MR-TADF emitter within a protective macrocyclic ring. We identify a previously undiscovered utility of macrocyclic encapsulation, whereby it can shield the MR core from the surrounding environment to enhance its radiative rate, PLQY, and reverse intersystem crossing (RISC) efficiency. Only with spectrally resolved transient photoluminescence measurements were we able to identify the weakly emissive aggregate and excimer species, and definitively confirm that the macrocycle suppresses their formation in the solid-state, thereby preserving narrowband deep-blue emission and reducing nonradiative losses. Notably, these performance enhancements were achieved without compromising thermal stability or vacuum-processability. When integrated into an OLED device based on the “hyperfluorescent” strategy, this emitter delivers an exceptional combined maximum external quantum efficiency (EQE) of 33% and (0.146, 0.046) CIE_*x*,*y*_ coordinates with peak emission at 451 nm, satisfying BT.2020 blue color requirement, and significantly outperforming its nonencapsulated analogue. This material represents one of the highest efficiency deep-blue OLEDs to date and therefore establishes macrocyclic encapsulation as a powerful synthetic strategy for unlocking the full potential of MR-TADF materials for next-generation OLEDs.

## Graphical abstract

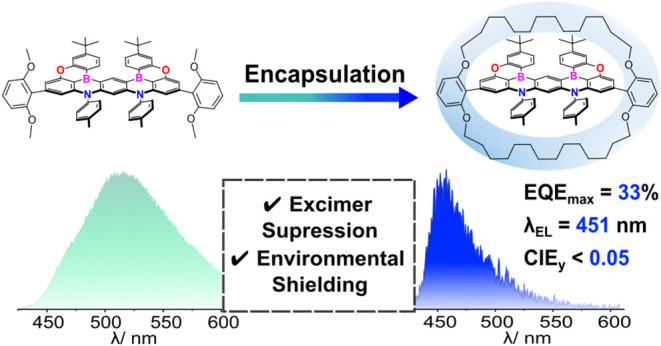

## Introduction

The continued development of luminescent materials is crucial for advancing the field of optoelectronics, particularly for their application in light-emitting devices. Purely organic luminescent materials generally consist of highly conjugated polycyclic structures, which provide an appropriate band gap for the absorption and emission of light. Recently, attention has focused on functionalizing these frameworks with electron-rich and electron-deficient heteroatoms or moieties (e.g oxygen, nitrogen, boron, carbonyl).^1,2^ These seemingly subtle structural changes can significantly alter the electronic structure, skewing the orbital distributions toward the electron-rich and electron-deficient dopants in the highest occupied molecular orbital (HOMO) and lowest unoccupied molecular orbital (LUMO), respectively. As a result, markedly different photophysical properties are observed compared to the parent molecule.

Specific dopant heteroatom arrangements can give rise to frontier molecular orbitals (FMOs) with nonbonding character which are spatially separated by their alternating distributions across the same molecular framework. Sufficient FMO separation reduces their exchange energy term and can yield a small energy gap between the first singlet (S_1_) and triplet (T_1_) excited states (Δ*E*_ST_).^3^ This energetic configuration can trigger a thermally activated delayed fluorescence (TADF) mechanism, allowing triplets to be upconverted into singlets using available thermal energy.^4^ These materials are known as ‘multi-resonant’ TADF emitters (MR-TADFs). Additionally, their unique FMOs also endow them with highly efficient narrowband emission, which results from their high radiative rate and a lack of vibronic coupling with high energy stretching modes.^1^

MR-TADFs are promising candidates for the emissive layer of organic light-emitting diodes (OLEDs), which have been established in display technologies and are emerging in the lighting industry. Their narrow emission provides color-pure electroluminescence (EL), while their TADF behavior facilitates harvesting of the 75% of electrically generated excitons that possess triplet spin multiplicity and would otherwise decay nonradiatively. For example, the archetypal boron–nitrogen doped MR-TADF ν-DABNA exhibits electroluminescence with a full width half-maximum (FWHM) of only 18 nm and a maximum external quantum efficiency (EQE) as high as 34.4% (Figure 1.).^5^ However, the applicability of MR-TADFs in OLEDs requires that the intrinsic properties of the isolated emitter are retained in a solid-state environment. Naturally, MR-TADFs have highly planar structures, making them vulnerable to through-space intermolecular interactions such as aggregation, excimer/exciplex formation, and exciton annihilation.^6^ These processes are detrimental to the EL performance because they can broaden and red-shift the spectrum, and they provide additional nonradiative decay pathways which reduce device efficiency.

**Figure 1:**
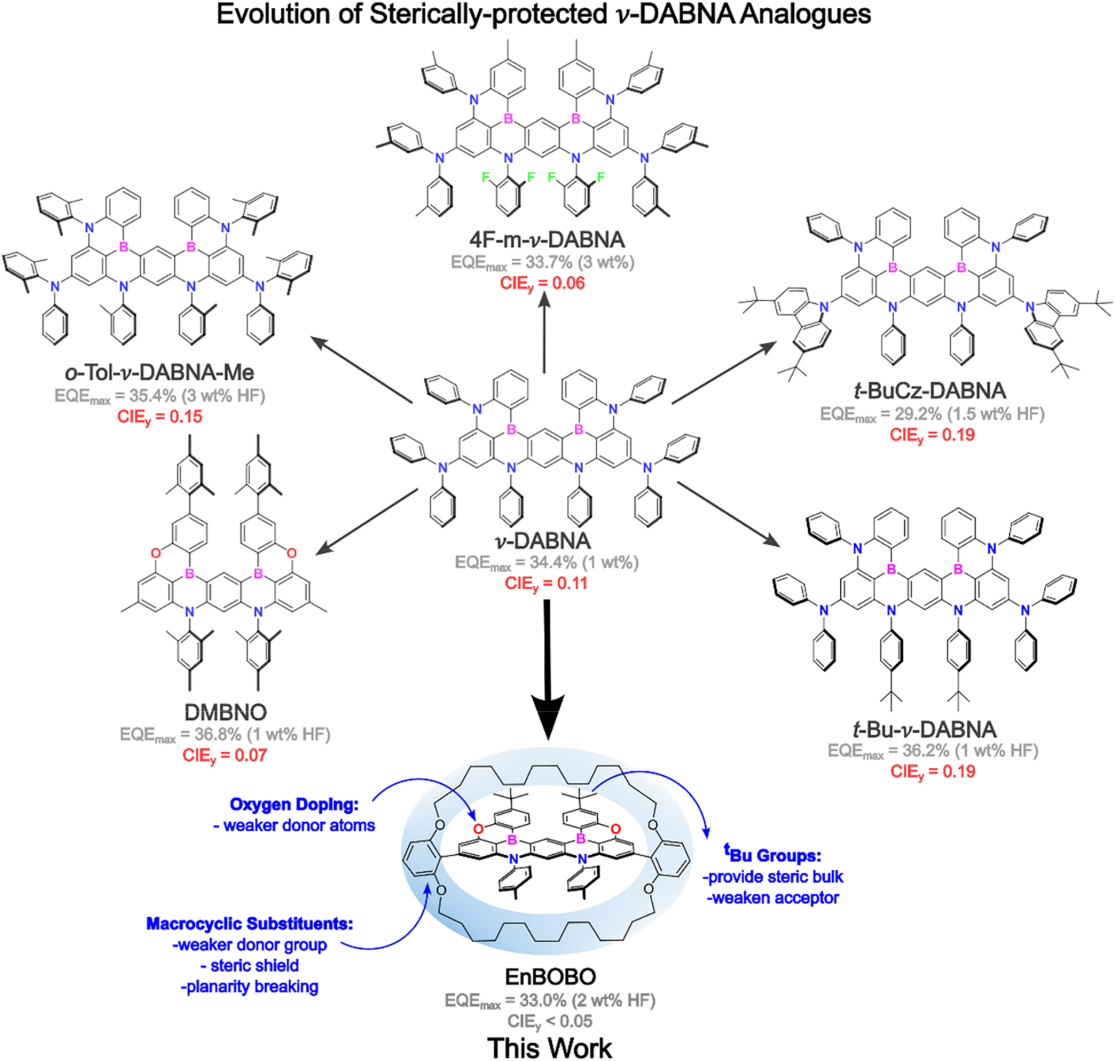
Previously reported ν-DABNA analogues which exhibit an element of steric protection in their molecular design. The molecular design presented in this work: a deep-blue ν-DABNA analogue which is sterically protected by macrocyclic covalent encapsulation.

Despite the model performance of ν-DABNA, it has been shown to exhibit excimer formation at device-relevant doping concentrations,^7^ and does not intrinsically satisfy the BT. 2020 blue color requirement. Accordingly, if these intermolecular processes could be understood and subsequently controlled, there is an opportunity for device performances to be enhanced even further.

Several recent publications have attempted to circumvent these issues by modifying the structure of ν-DABNA through the introduction of steric groups intended to reduce these undesirable processes and their effect on device performance (Figure 1).^8–12^ Although these emitters have seemingly minor variations in the positioning of their alkyl substituents (methyl, *tert*-butyl), their performance in the solid-state varies, with the degree of steric protection provided being difficult to predict. This inconsistency highlights the clear need for new molecular design approaches that can reliably introduce steric protection to enhance device performance. Additionally, in most cases these emitters also fail to meet the expected color purity requirements.

Recently, we presented an alternative synthetic strategy to improve the performance of blue OLEDs through the steric protection of a narrowband terminal emitter to maintain color purity and suppress intermolecular processes in a device environment, such as Dexter energy transfer.^13^ This ‘encapsulation’ strategy involves introducing aryl substituents which bear a macrocyclic ring via an ether linkage. This strategy functions by the macrocycle acting as a steric shield to break the planarity of the polycyclic emitter so its intrinsic photophysical properties can be maintained in the solid-state. It follows that the encapsulation of a ν-DABNA analogue is a promising avenue for the development of high-performance OLEDs.

Herein, we report the first encapsulated deep-blue MR-TADF emitter which exhibits enhanced efficiency and color purity versus its nonencapsulated analogue and results in an OLED device which satisfies the BT.2020 CIEy color requirement with maximum emission at 451 nm and a maximum external quantum efficiency of 33%. We use spectrally resolved transient photoluminescence studies to attribute our efficiency enhancements to a reduction in aggregate and excimer formation, and we attribute enhanced color purity also to this, in addition to the ability of the encapsulating macrocycle to shield and protect the core of the emitter from the device environment. This work further demonstrates the utility of the encapsulation strategy in OLED devices.

## Results and Discussion

When designing the encapsulated MR-TADF emitter, we envisioned replacing the peripheral diphenylamine groups of ν-DABNA with aryl substituents bearing a macrocycle that encases the MR core (see Figure 1). However, these carbon-linked substituents are less electron-donating than diphenylamine and therefore increase the electron-withdrawing strength of the *para* boron atoms.^14^ As a result, the intrinsic luminescence would undesirably red-shift by a stabilization of the LUMO.^15^ To counteract this electronic effect, and to shift the emission into the deep-blue region, two of the phenylamine groups were replaced with less electron-donating oxygen atoms to decrease the HOMO energy.^8,14,16–18^ Additionally, weakly donating tertiary butyl groups were introduced at positions *para* to the boron atoms to reduce their withdrawing strength and provide steric bulk at sites furthest from the encapsulating substituents.^9^

The synthetic route to this blue-shifted and encapsulated MR-TADF emitter (**EnBOBO**) is outlined in Scheme 1 (see S2 for the complete synthetic route and procedures). The synthesis began with the preparation of a doubly halogenated MR core (**Compound 1**) in four steps from commercially available starting materials. This portion of the route, inspired by previous work,^8,10,14,17^ involved nucleophilic aromatic substitution of an aryl fluoride with a phenol to form an ether linkage, two Buchwald–Hartwig amination steps to form tertiary amine units, and a regioselective lithium-free one-shot double borylation. This borylation step was performed in a sealed system using deactivated trichlorobenzene as the solvent and boron tribromide (BBr_3_) as the borylating reagent. As seen previously,^2,19^ the positive mesomeric effect of the diphenylamine units in the pre- borylation scaffold should direct initial electrophilic aromatic substitution with BBr_3_ to their sterically accessible *para* positions on the central and most activated ring. This intermolecular C–B bond formation is then followed by rapid intramolecular borylation with the oxygen-substituted rings and the new BBr_2_ substituent to give this MR framework.^2,20^

**Scheme 1:**
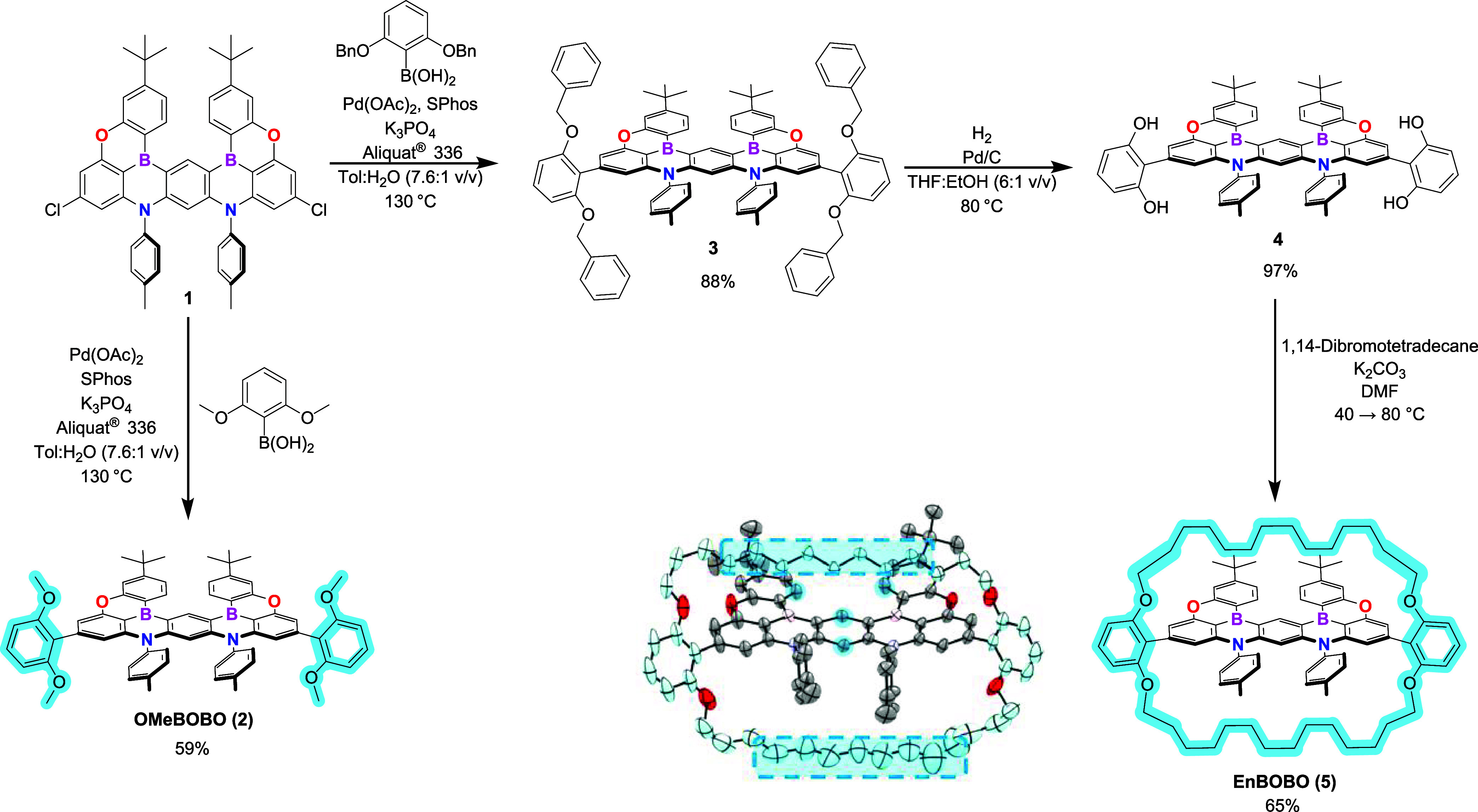
Synthetic Route towards OMeBOBO and EnBOBO^a^

a The extent of encapsulation is highlighted in blue. The X-ray crystal structure of EnBOBO is shown and the aromatic and alkyl proton environments which exhibit ROEs with each other are highlighted in blue.

The two chlorine atoms of **Compound 1** enabled postborylation peripheral functionalization of the MR core, where palladium-catalyzed cross-coupling chemistry was used to install the encapsulating substituents. Following our previous work,^13^ doubly benzyl-protected encapsulating groups were introduced via a Suzuki–Miyaura cross-coupling reaction (**Compound 3**) and were subsequently deprotected by palladium-catalyzed hydrogenation to afford **Compound 4**. Finally, the macrocycle’s encasing alkyl straps were installed via nucleophilic substitution under dilute and basic conditions in a polar aprotic solvent, giving **EnBOBO** (**Compound 5**) in a 65% yield. Additionally, a nonencapsulated control molecule **OMeBOBO** (**Compound 2**), featuring peripheral dimethoxyaryl substituents, was similarly prepared from **Compound 1**. This allowed the impact of alkyl chain encapsulation on emitter performance to be determined by comparison. Notably, postborylation functionalization is necessary in the synthesis of **OMeBOBO**, as the Lewis acidic BBr_3_ would otherwise deprotect the methoxy ethers to their corresponding alcohols.^2^

The identity of all final products and synthetic intermediates was confirmed by solution-state ^1^H and ^13^C nuclear magnetic resonance (NMR) spectroscopy and high- resolution mass spectrometry (HRMS), while the purity was assessed by elemental/HPLC analysis (see S2–S3 for complete characterization data and NMR spectra). To assess the effectiveness of our synthetic strategy in encapsulating the MR core, we first examined the ^1^H NMR spectra of **EnBOBO** and the free 1,14-dibromotetradecane chain used in the macrocyclization step (see Figure S26). A comparison of these spectra reveals that macrocyclization induces diastereotopicity in the three methylene groups closest to the oxygen linkage of **EnBOBO**, with the two protons on each carbon appearing as distinct resonances. As proposed in our previous work,^21^ we attribute this observation to the reduced conformational freedom of the alkyl chains in **EnBOBO**. Additionally, the alkyl environments experience an upfield shift upon macrocyclization, which is consistent with expected shielding from the ring current of the aromatic MR core.^21^ The exception to this is the terminal methylene environment, which experiences a downfield shift from 2.98 ppm to approximately 3.58 ppm due to substitution of bromine with more electron-withdrawing oxygen. Furthermore, the ^1^H–^1^H ROESY spectrum of **EnBOBO** (see Figure S27) exhibits significant ROEs between the eight most central methylene environments of the alkyl chains and the central aromatic environments of the MR core (Scheme 1), which are assigned using the characteristic downfield shift of protons in proximity to boron,^22^ integrals, and multiplicities. Overall, this NMR data provides strong indication that the alkyl chains encapsulate the MR core by lying above and below its plane.

An X-ray crystal structure of **EnBOBO** (see Scheme 1 and Figure S28) unequivocally confirms the encapsulation and demonstrates that the macrocycle enhances the dimensionality of the molecular unit. First evidence of the utility of the macrocycle is observed from its crystal packing (see Figure S29). The crystal structure comprises molecules lying in layers (parallel to the **ac** plane of the unit cell), with the MR cores embedded within the layers and molecules in adjacent layers meeting end-on. Within each layer, the planes of the MR cores are parallel, but the macrocycle enforces a minimum separation of 9.15 Å between molecular centroids, which is far greater than a typical π–π stacking distance. The alkyl chains in neighboring molecules adopt an aligned parallel arrangement similar to that seen in *n*-alkane crystal structures, with a perpendicular separation <4 Å, and it is likely that the dispersion interactions along the length of these chains provide a significant driving force for the observed packing arrangement. The crystal structure demonstrates how the macrocycle can prevent close intermolecular contacts between MR cores in a solid-state environment, which could otherwise promote undesirable processes such as aggregation or excimer formation.^6^

Thermogravimetric analysis (see Figure S30) demonstrates that both emitters are highly thermally stable, with a 5% weight loss occurring at 436 °C, making them suitable for the fabrication of industrially relevant evaporated OLEDs and films. Importantly, the macrocycle does not compromise thermal stability, which suggests that the high-temperature degradation process originates from the MR core itself.

We next characterized the intrinsic photophysical properties of the emitters using dilute solution-state spectroscopic measurements (see S7 for full experimental details). The results are shown in Figure 2 and the key photophysical parameters are summarized in Table 1. The emitters exhibit similar molar extinction profiles, each featuring a distinct, strong (ε­(λ_max_) = ∼42,000 dm^3^ mol^–1^ cm^–1^), and narrow (∼0.13 eV) absorption band in the visible region. The photoluminescence (PL) spectra appear as near mirror images of their lowest-energy absorption band. Based on this spectral symmetry, and the TD-DFT calculations for **OMeBOBO** (see S8 for full details) which predict an allowed S_0_–S_1_ transition (*f* = 0.423), the absorption and PL bands are assigned to the S_0_–S_1_ absorption and the corresponding S_1_–S_0_ fluorescence, respectively. As intended by our molecular design, the emitters display narrowband and deep-blue emission. A blue-shift of at least 16 nm relative to ν-DABNA (λ_PL_ = 468 nm) is observed and both emitters achieve CIEy < 0.05, approaching the BT.2020 blue color requirement.^5,23^ The narrow spectral profiles suggest there is minimal vibronic coupling between the S_0_–S_1_ electronic transition and high-frequency vibrational modes, while the small Stokes shift implies the S_1_ state experiences limited structural relaxation following photoexcitation. These spectral features are defining characteristics of MR-TADF emitters, and confirm that the S_1_ state retains MR character despite the structural modifications required to widen the band gap.^1^ Hole–electron density analysis of the TD-DFT calculations (see S8 for full details) further supports this, with the hole and electron of the S_1_ state being spatially separated over the same molecular framework (see Figures S41–S44) to give short-range charge-transfer (SRCT) character.^24^ Additionally, positive solvatochromism is observed for both emitters, confirming that the S_1_ state possesses charge-transfer character.

**Table 1:** A Summary of the Solution-state Photophysical Properties of OMeBOBO and EnBOBO in Toluene

	λ_abs_ ^a^/nm	λ_PL_ ^b^/nm	Stokes^c^/cm^–1^ (eV)	FWHM^d^/nm (eV)	CIE_*xy*_ ^e^	ϕ^f^/%	τ_p_ ^g^/ns	τ_d_ ^h^/μs	ϕ_d_/ϕ_p_ ^i^	*k*_r_ ^j^/10^8^ s^–1^	*k*_ISC_ ^k^/10^7^ s^–1^	*k*_RISC_ ^l^/10^4^ s^–1^
**OMeBOBO**	438	452	707 (0.088)	24 (0.14)	(0.144, 0.049)	78	5.4	41	0.12	1.3	5.7	0.96
**EnBOBO**	435	445	517 (0.064)	19 (0.11)	(0.153, 0.031)	91	5.3	12	0.05	1.6	2.5	3.2

a Wavelength of maximum absorbance of the lowest energy band.

b Wavelength of maximum photoluminescence.

c Stokes shift for the lowest energy absorbance band and the emission spectrum.

d The full width at half-maximum of the photoluminescence spectrum.

e Commission internationale de l’éclairage 1931 color coordinates of the photoluminescence.

f Photoluminescence quantum yield.

g Prompt fluorescence lifetime.

h Delayed fluorescence lifetime.

i Ratio between the delayed and prompt fluorescence contribution to the photoluminescence quantum yield.

j Radiative rate constant.

k Intersystem crossing rate constant.

l Reverse intersystem crossing rate constant.

**Figure 2:**
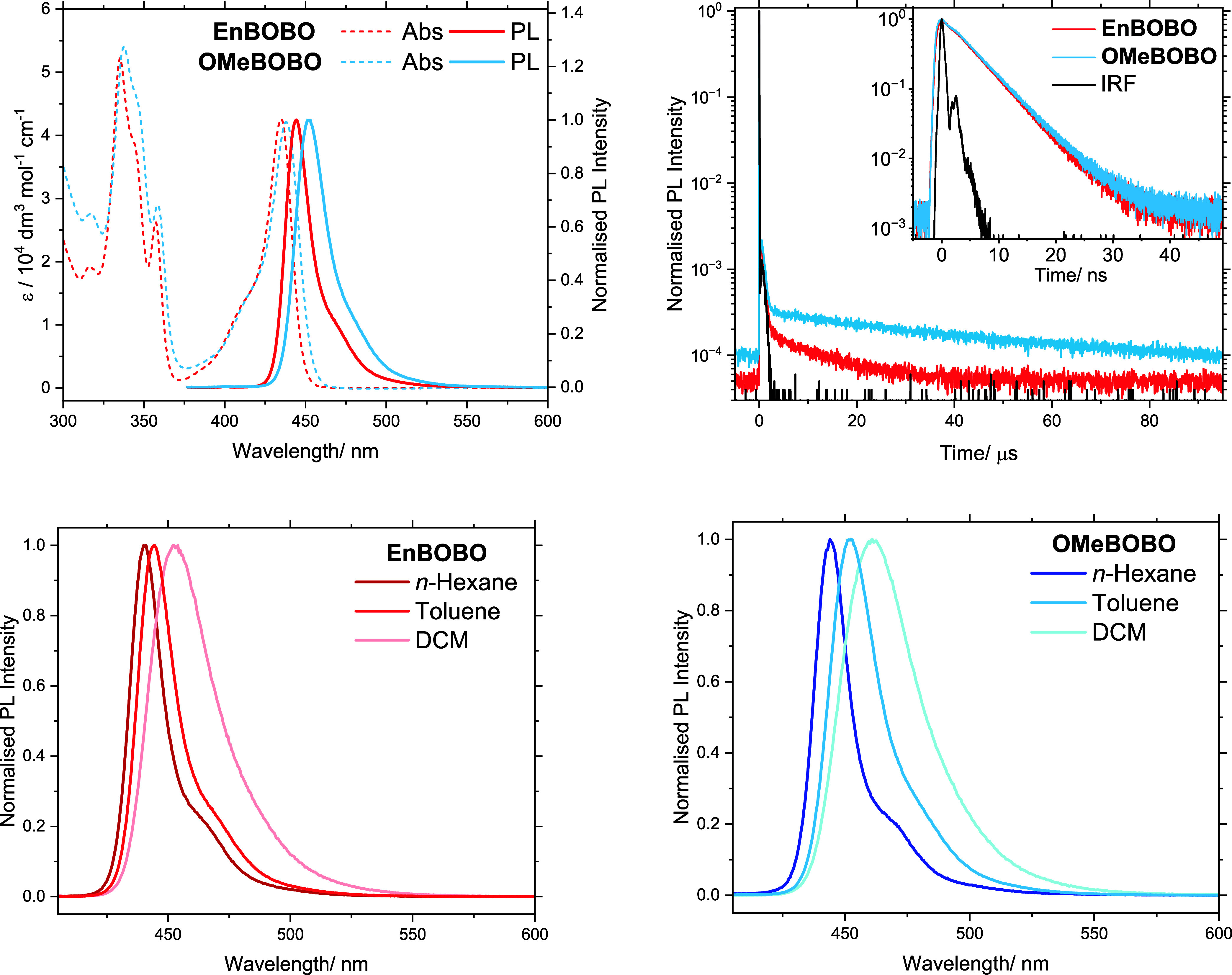
Solution-state photophysical properties of **OMeBOBO** and **EnBOBO** in toluene.

Despite sharing the same MR core, the photophysical properties of the emitters differ marginally, with **OMeBOBO** showing slightly red-shifted and broader spectra compared to **EnBOBO**, particularly in the photoluminescence. **OMeBOBO** also displays greater sensitivity to the solvent polarity, with more pronounced red-shifts and broadening observed across its solvatochromic series. This trend suggests that the S_1_ state of **OMeBOBO** exhibits a greater charge-transfer character versus **EnBOBO**. We propose that the encapsulating macrocycle of **EnBOBO** shields the dipole of the S_1_ state from interacting with the solvent environment. This finding is supported by the identical PL FWHM observed for both emitters in nonpolar *n*-hexane (see Table S4), although **OMeBOBO** remains red-shifted by 4 nm here, indicating that there is an intrinsic bandgap difference between the two emitters. These findings highlight an unanticipated utility of the macrocycle in which it can insulate the emissive core from its environment, protecting it from undesirable spectral shifting and broadening. As a result, **EnBOBO** retains narrower and deeper-blue emission even in polar media, a property that is advantageous in device-relevant solid-state environments.

Transient photoluminescence measurements reveal the emitters exhibit prompt and delayed contributions to their total fluorescence. Identical PL spectra are observed at both nanosecond and microsecond times (see Figure S36), confirming that these contributions both originate from the same S_1_ state. This behavior is assigned to TADF based on the MR/SRCT character of the S_1_ state and the measured singlet–triplet energy gap (Δ*E*_ST_) of 0.19 eV (see Figure S38). Determining photophysical rate constants and PL contributions reveals further differences between the two emitters. First, the photoluminescence quantum yield of **EnBOBO** is 13% higher than that of **OMeBOBO** (91% vs 78%) and can be attributed to its greater radiative rate (1.6 vs 1.3 × 10^8^ s^–1^) and reduced intersystem crossing rate (ISC) (2.5 vs 5.7 × 10^7^ s^–1^), as both emitters exhibit similar prompt lifetimes (see S7). Second, the delayed lifetime is approximately three times shorter in **EnBOBO** (12 vs 41 μs), while the delayed contribution to total photoluminescence is roughly twice as high in **OMeBOBO** (11 vs 5%). Together, these differences suggest that the larger delayed contribution of **OMeBOBO** arises from a more efficient ISC process, which generates a greater population of triplets following photoexcitation. Conversely, the smaller delayed contribution of **EnBOBO** is due to its reduced ISC rate, although the triplets that form are more efficiently upconverted to the S_1_ state by its higher reverse intersystem crossing (RISC) rate. These findings were unexpected given the shared MR cores of the emitters, prompting a consideration of potential differences in their ISC/RISC mechanisms. TD-DFT calculations (see S8), along with low-temperature fluorescence and phosphorescence measurements (see Figure S38), indicate that the S_1_ and T_1_ states possess similar character, suggesting that direct RISC between them may be inefficient, and that higher-energy triplet states of differing character are likely involved.^7,25^ Specifically, we calculate intermediate T_2_ and T_3_ states with a different electronic character to S_1_ (see Figures S41–S44), both of which show increased SOCME values for the spin conversion to S_1_ versus the T_1_ state (see Table S15). Interestingly, the macrocycle also appears to enhance this spin-vibronic mechanism, which could explain the enhanced RISC rate of **EnBOBO**. Additionally, we propose that the ISC/RISC efficiency in these systems is highly sensitive to the exact energetic ordering within the excited state manifolds. In the case of **OMeBOBO**, the absence of the macrocycle exposes the MR core to the solvent environment, enhancing the interaction between its electronic states and the surrounding medium, especially for states with greater CT character, which could influence the ISC/RISC efficiency.

Having confirmed that our molecular design yields an encapsulated MR-TADF emitter with intrinsic deep-blue photoluminescence, we next turned to its electroluminescence (EL) performance. To assess this, we fabricated TADF-sensitized (hyperfluorescent­(HF)) OLED devices which applied the BOBO materials as terminal emitters (see S9 for full details).^26^ HF architectures are appropriate here because the emitters combine color-pure emission with a high radiative rate to achieve a high photoluminescence quantum yield, while exhibiting only a modest RISC rate. Notably, the TADF behavior of the emitters may still provide benefit, as any triplet excitons that reach the terminal emitters by ISC or direct charge recombination can still be harvested and contribute to emission.^12^ A key requirement for successful HF is the efficient Förster resonance energy transfer (FRET) of more energetic sensitizer singlets to the terminal emitter. FRET efficiency can be assessed by considering the spectral overlap of the sensitizer emission and the terminal emitter absorption. We selected two recently reported blue TADF emitters, **TDBA-PAS** and **TDBA-SPQ**,^27,28^ as our sensitizers because they exhibit high photoluminescence quantum yields (>90%) and RISC rates (∼10^6^ s^–1^). The molecular structures of these sensitizers and their doped-film PL spectra overlaid with the molar extinction spectra of the BOBO emitters is given in Figure 3. The sensitizers feature a boron–oxygen doped MR acceptor unit and a nitrogen-containing donor unit. **TDBA-PAS** has the greatest overlap with the terminal emitter spectra as it exhibits deep-blue emission, while **OMeBOBO** exhibits slightly enhanced overlap with both sensitizer spectra versus **EnBOBO**, because of its narrower band gap (see Table S6 for calculated overlap integrals). Additionally, cyclic voltammetry (see S6) suggests the terminal emitters possess virtually isoenergetic or deeper HOMO levels versus the TADF sensitizers, reducing the likelihood of hole-trapping by the BOBO emitters. This energetic alignment is favorable in hyperfluorescent systems, as trapping on the terminal emitter could result in polaron–exciton annihilation or trap-assisted recombination, both of which reduce device performance.

**Figure 3:**
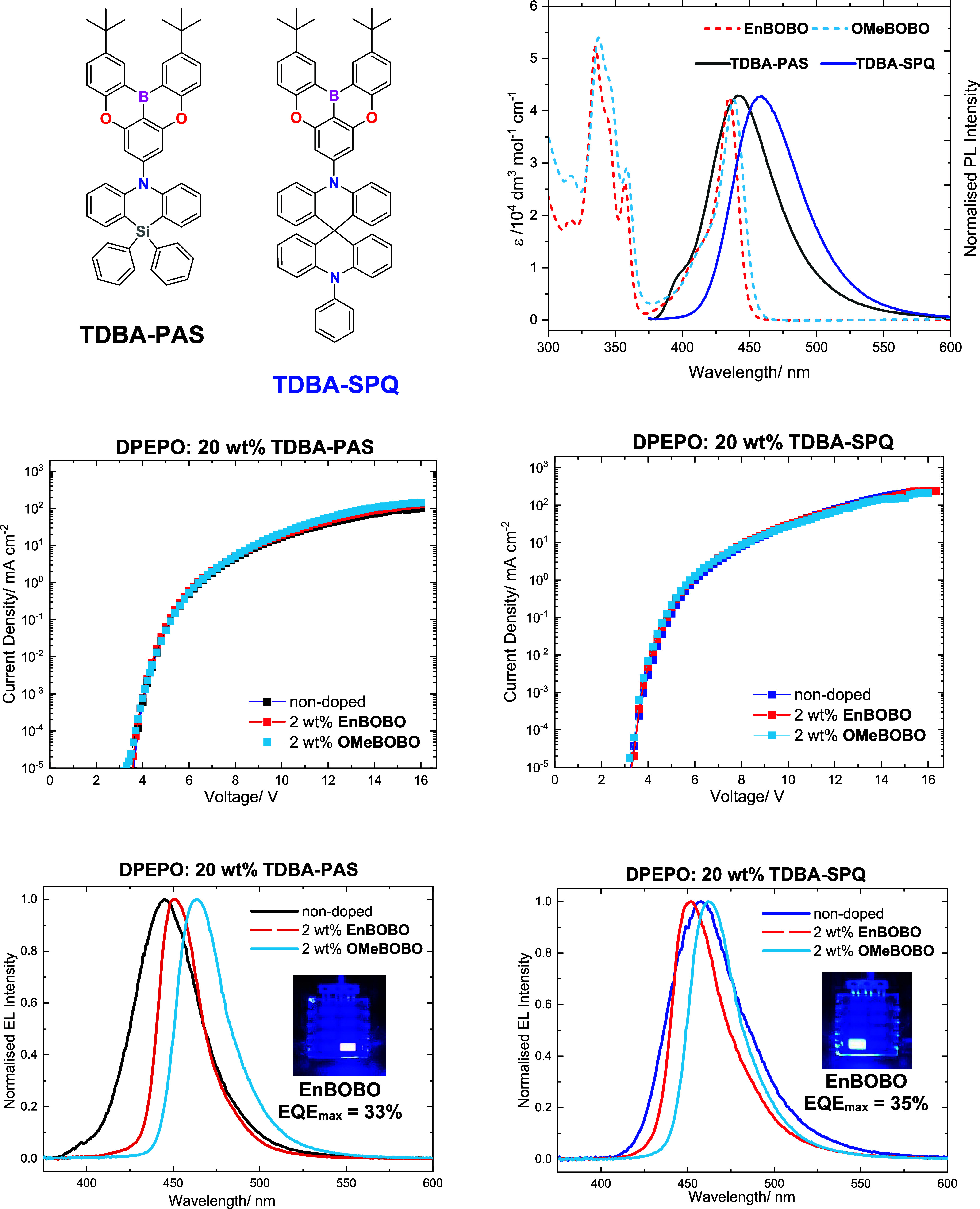
Molecular structures of the TADF sensitizers: **TDBA-PAS** and **TDBA-SPQ**. The spectral overlap of the molar extinction spectra of **OMeBOBO** and **EnBOBO** (dashed lines) with the photoluminescence spectra of 20 wt % of **TDBA-PAS** and **TDBA-SPQ** doped into DPEPO (20 wt %, solid lines). The current–voltage characteristics of the nondoped (20 wt % TADF sensitizer in DPEPO) and the doped devices (2 wt % BOBO emitter and 20 wt % TADF sensitizer in DPEPO) and their corresponding electroluminescence spectra.

The OLED devices were fabricated using a conventional multilayer architecture (see S9 for details). The emissive layer (EML), deposited between the hole-transport layer and the electron-transport layer, consists of a ternary blend of DPEPO, 20 wt % of either **TDBA-PAS** or **TDBA-SPQ**, and 1, 2, or 5 wt % of either **EnBOBO** or **OMeBOBO**. Here, DPEPO was selected as a host matrix for its wide band gap and high triplet energy, which directs charges and excitons to the rest of the emissive layer.^29,30^

The best-performing OLEDs were those incorporating 2 wt % of the terminal emitter. The current density–voltage characteristics and EL spectra of these devices are shown in Figure 3, alongside the nondoped devices based solely on the TADF sensitizers, while their performance metrics are summarized in Table 2 (see S9 for device performance of all doping concentrations). The nondoped devices exhibit blue EL in which **TDBA-PAS** produces deep-blue emission with a maximum at 445 nm, satisfying the BT.2020 color requirement (CIE_*y*_ < 0.05), while **TDBA-SPQ** delivers superior external quantum efficiency (EQE) and roll-off characteristics (EQE_max_ = 33.1%, EQE_100_ = 30%). As expected for donor–acceptor TADF emitters, both sensitizers show relatively broad EL spectra due to the long-range CT character of their S_1_ states.^4^ Doping the emissive layer with the BOBO emitters narrows the EL spectra, which is consistent with efficient FRET from the sensitizer. However, the EL remains broader and red-shifted compared to the corresponding toluene solution measurements, especially in the case of **OMeBOBO**. We attribute this difference to a combination of the enhanced polarity of the solid-state device environment, inhomogeneous broadening, and potentially incomplete energy transfer and aggregate/excimer formation in the case of **EnBOBO** and **OMeBOBO**, respectively. The current density–voltage characteristics are virtually unchanged upon doping, suggesting these emitters do not function as charge-carrier traps. Additionally, the EQE roll-off characteristics are retained upon doping (see S9), especially in the case of **EnBOBO**, suggesting that device stability and exciton quenching mechanisms are dictated by DPEPO and the TADF sensitizer. Thus, the introduction of terminal emitters is not expected to reduce the stability of these devices. We also observe EQE enhancements relative to the nondoped devices when the BOBO emitters are introduced, which we attribute to their enhanced alignment (see Figure S39) and resulting outcoupling,^12^ and perhaps the TADF behavior of the terminal emitters themselves.

**Table 2:** A Summary of the OLED Device Performance Metrics

device	*V*_ON_ ^a^/V	EQE_max_ ^b^/%	EQE_100_ ^c^/%	λ_EL_ ^d^/nm	FWHM^e^/nm (eV)	CIE_*xy*_ ^f^
**DPEPO**: 20 wt % **TDBA-PAS**	3.8	22.7	15	445	43 (0.25)	0.152, 0.044
2 wt % **EnBOBO**	3.7	33.0	20	451	28 (0.16)	0.146, 0.046
2 wt % **OMeBOBO**	3.7	25.1	14	464	33 (0.18)	0.131, 0.102
**DPEPO:** 20 wt % TDBA-SPQ	3.5	33.1	30	458	50 (0.27)	0.144, 0.098
2 wt % **EnBOBO**	3.4	34.9	31	452	34 (0.19)	0.144, 0.074
2 wt % **OMeBOBO**	3.3	34.5	27	462	32 (0.17)	0.133, 0.098

a Device turn-on voltage.

b The maximum external quantum efficiency.

c The external quantum efficiency at 100 cd m^–2^.

d Wavelength of maximum electroluminescence intensity.

e Full width half-maximum of the electroluminescence spectrum.

f Commission internationale de l’éclairage 1931 color coordinates of the electroluminescence.

These device results clearly demonstrate the beneficial influence of the macrocycle on the electroluminescent properties. Devices incorporating **EnBOBO** exhibit bluer EL with improved CIEy coordinates compared to those using **OMeBOBO**, which we attribute to a combination of **EnBOBO**’s intrinsically wider band gap, its resistance to environmentally driven spectral red-shifting and broadening, and probably reduced aggregate/excimer formation. In fact, the **TDBA-PAS** devices exhibit an electroluminescence maximum of 451 nm with CIE_*xy*_ coordinates of (0.146, 0.046) when incorporating **EnBOBO**. However, in devices using **TDBA-SPQ** as the sensitizer, **EnBOBO** shows broader EL than **OMeBOBO**. This is likely due to incomplete energy transfer, highlighting the importance of optimal spectral overlap in the HF strategy. In terms of efficiency, the **EnBOBO** devices outperform their **OMeBOBO** counterparts in all cases (33.0 vs 25.1% for **TDBA-PAS** devices and 34.9 vs 34.5% for **TDBA-SPQ** devices). While this can be partially attributed to the higher PLQY and RISC efficiency of **EnBOBO**, the magnitude of improvement is highly sensitizer dependent. Only a modest 0.4% increase in the EQE_max_ is observed with **TDBA-SPQ**, whereas an impressive increase of ∼10% is seen with **TDBA-PAS**. This clear sensitizer dependence highlights the complex and interesting interplay between macrocycle protection and the sensitizer environment in determining device performance. Comparison of the EL of **OMeBOBO** doped with the two sensitizers (λ_EL_ = 464 and 462 nm for **TDBA-PAS** and **TDBA-SPQ** devices, respectively), suggests the polarity of the device environment is similar and therefore any observed differences likely do not arise from differences in terminal emitter intrinsic photophysical parameters, but how each sensitizer modulates the nonradiative loss pathways resulting from solid-state intermolecular interactions.

It is important to try and understand the origin of this sensitizer-dependent device efficiency performance between the BOBO emitters. To elucidate this, we turned to spectrally resolved transient photoluminescence measurements of the device films. These results are presented in Figure 4 and summarized in Table 3 (See S7 for full details and data).

**Table 3:** A Summary of the Photophysical Properties of the 2 wt % BOBO Emitter Device Films^5^

film	λ_PL_ ^a^/nm	FWHM^b^/nm (eV)	τ_p_ ^c^/ns
**DPEPO:** 20 wt % TDBA-PAS	441	57 (0.32)	9.9
2 wt % **OMeBOBO**	465	35 (0.19)	5.1
2 wt % **EnBOBO**	449	30 (0.17)	6.8
**DPEPO:** 20 wt % TDBA-SPQ	459	60 (0.31)	20
2 wt % **OMeBOBO**	467	36 (0.19)	5.6
2 wt % **EnBOBO**	451	32 (0.18)	10

a Wavelength of maximum steady-state photoluminescence intensity.

b Full width half-maximum of the steady-state photoluminescence spectrum.

c Prompt fluorescence decay lifetime.

**Figure 4:**
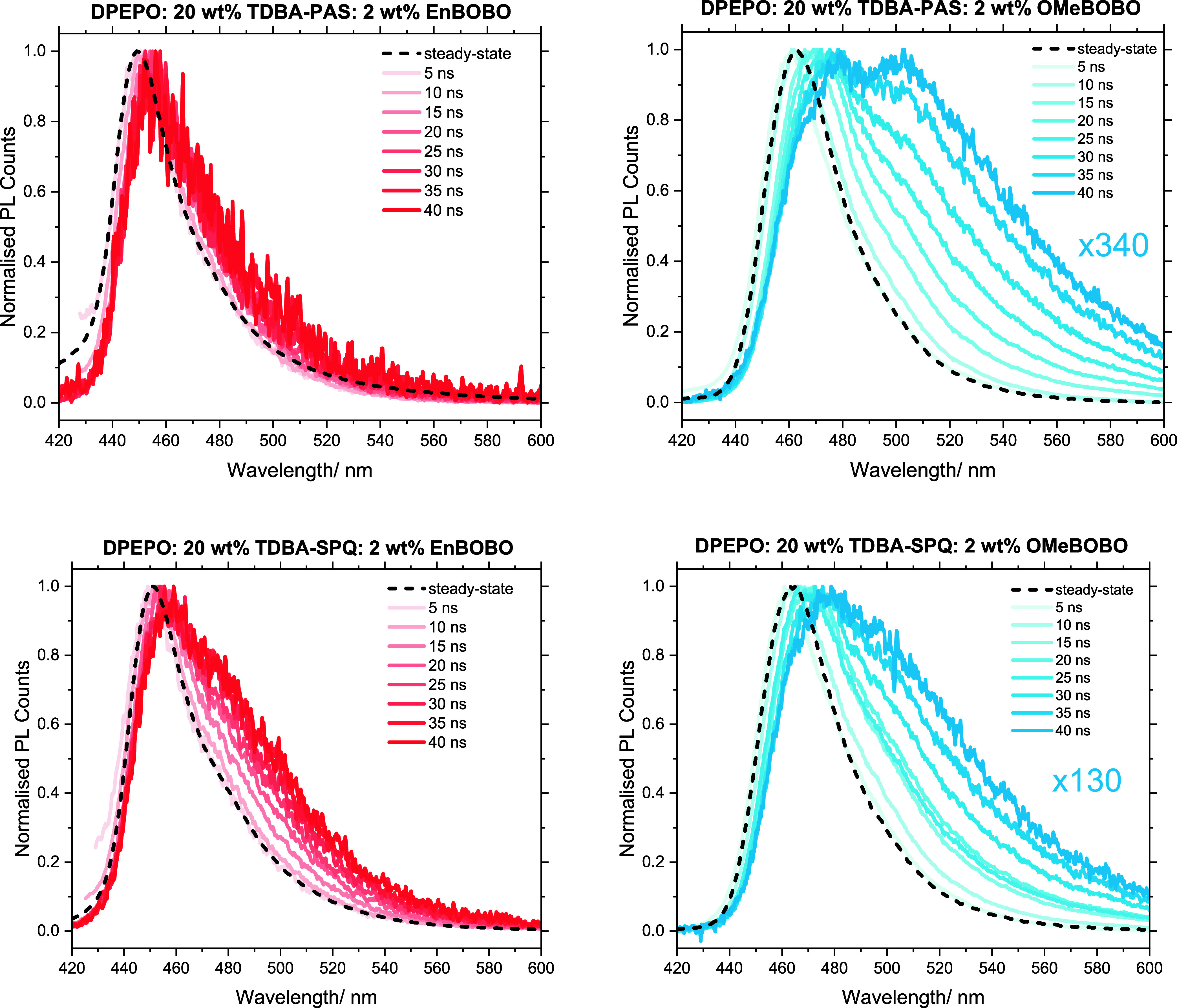
Steady-state and time-gated (5–40 ns) photoluminescence spectra of the 2 wt % BOBO emitter device films. The numerical multiplication factors represent the relative intensity of the photoluminescence at 5 ns and the intensity of the lower energy emissive species at 40 ns.

First, we considered the **TDBA-PAS** films. The time-gated photoluminescence spectra of the **EnBOBO** films show little evolution, suggesting the presence of a single emissive species that inhabits a similar environment. These spectra confirm that the encapsulating macrocycle is effective at suppressing undesirable solid-state interactions such as excimer or exciplex formation. In contrast, when **OMeBOBO** is used, a much more complex picture emerges. From 5 ns we observe a gradual red-shifting of the emission maxima from around 460 to 480 nm. From 15 to 20 ns we also observe the growth of a new and even more red-shifted emissive species centered around ∼500 nm at 40 ns. We attribute these new emissive species to aggregation in the solid state. It is not possible to determine the exact nature of these states, but we tentatively suggest that they are a combination of weakly aggregated (responsible for the gradual red-shift) and excimer-like strongly aggregated (responsible for the emission at ∼500 nm) species, with their growth over time suggesting they originate from the photogenerated S_1_ state. Importantly, these features are only clearly resolved using transient methods and are not visible in the steady-state emission spectrum due to their weak contribution (lower PLQY) to the overall photoluminescence. Thus, we propose that our encapsulation strategy effectively suppresses aggregation-induced quenching and spectral distortion by reducing nonradiative losses as well as red-shifting and broadening of the EL that compromise device efficiency and color purity, respectively.

Now we turn to the use of **TDBA-SPQ** as the sensitizer. With **EnBOBO** we now observe a very slight red-shift in emission maxima from around 450 nm to just below 460 nm between 5 and 40 ns, and we again observe the growth of a contribution from a lower energy emitting species. Turning to **OMeBOBO**, we see similar but substantially more pronounced behavior due to the lack of the shielding macrocycle. Therefore, in both the **TDBA-SPQ** layers the terminal emitters are involved in substantially more intermolecular interactions. Remarkably, despite these clear spectroscopic markers of aggregation, and to varying degrees, both terminal emitters still display highly competitive efficiencies and reasonable color coordinates. This highlights that if aggregate behavior could be further understood and controlled even higher device efficiencies should be possible. Furthermore, we note that photoluminescence techniques can only probe “bright” aggregates, and it is possible that dark aggregates that limit the device performance are also formed.

In terms of excited state dynamics, a reduction in the prompt lifetime upon doping the films with the BOBO emitters confirms that FRET is taking place between the sensitizers and these emitters (Table 3). The prompt lifetime of the **OMeBOBO:TDBA-PAS** device film is shorter than the solution lifetime of **OMeBOBO** (5.4 ns), further suggesting that **OMeBOBO** experiences additional nonradiative decay processes in the solid-state. The delayed portion of the photoluminescence decays of these device films is complex (see Figure S37), and as anticipated, this suggests that both the sensitizer the terminal emitter are contributing to triplet harvesting. The PL at the shorter delayed time scales can be assigned to the sensitizers as they have shorter delayed lifetimes (<10 μs), while the delayed PL at the longest time scales can be assigned to the terminal emitters as they exhibit longer delayed lifetimes (>10 μs).

## Conclusion

We have introduced a molecular design strategy that achieves an impressive electroluminescence efficiency and color purity combination through the incorporation of a covalently linked protective macrocyclic ring. The macrocycle was shown to enforce large intermolecular separations in a crystalline state, which manifests as suppressed aggregation and excimer formation in device films. In addition, we reveal a previously unidentified advantage of macrocyclic encapsulation, namely its ability to shield the emissive core from the immediate environment. This shielding results in materials that exhibit narrower and blue-shifted emission, along with enhanced radiative and reverse intersystem crossing rates. Importantly, all these benefits are realized without compromising thermal stability or vacuum processability, ensuring compatibility with OLED fabrication. Together, these effects deliver significant improvements in device performance when compared with a nonencapsulated analogue material. The enhanced color purity arises from the dual action of aggregation suppression and environmental shielding, while the boosted efficiency is linked to the suppression of nonradiative decay associated with the formation of aggregates and excimers. Notably, spectrally resolved transient photoluminescence measurements were essential for identifying the presence of these weakly emissive species, which appear hidden in their steady-state spectra. These measurements also revealed that terminal emitters with TADF activity can contribute to overall triplet harvesting and further boost the efficiency in hyperfluorescent architectures. Overall, our results establish macrocyclic encapsulation as a uniquely powerful strategy that unites intermolecular interaction suppression with environmental shielding. We envisage that this work further secures this ‘bottom-up’ synthetic approach as a promising new paradigm for the development of next-generation OLED materials.

## Supplementary Material



## Notes

Full synthetic procedures and additional optoelectronic characterization (PDF)
