# Supplementary material for: Macrocyclic Covalent Encapsulation of a Multi-Resonant Emitter: Understanding and Controlling Interactions in Highly Efficient Deep-Blue OLEDs
Source: J Am Chem Soc. 2026 Feb 17;148(8):8163–73. doi: 10.1021/jacs.5c16290 (PMC12964404; doi:10.1021/jacs.5c16290)
Supplement: Supplementary file 1 [file ja5c16290_si_001.pdf]

SUPPORTING INFORMATION

Macrocyclic Covalent Encapsulation of a Multi-resonant Emitter: Understanding and Controlling Interactions in Highly Efficient Deep-blue OLEDs

Erin M. Holdsworth,[a,b] Hwan-Hee Cho,[b,c] Andrew D. Bond,[a] Stephanie Montanaro,[a,d] Seung-Je Woo,[b] Tianyu Huang,[a] Jordan Shaikh,[a] Fathy Hassan,[a,e] Sebastian Gorgon,[b] Víctor Riesgo-Gonzalez,[d] Alexander J. Gillett,[b,f] Daniel G. Congrave,[a,d] Richard H. Friend,[b] and Hugo A. Bronstein.[a,b,*]

[a]*Yusuf Hamied Department of Chemistry, University of Cambridge, Cambridge, CB2 1EW, United Kingdom*

[b]*Cavendish Laboratory, University of Cambridge, Cambridge, CB3 0HE, United Kingdom*

[c]*Department of Materials Science and Engineering, Yonsei University, 50 Yonsei-ro, Seodaemun-gu, Seoul, 03722, Republic of Korea*

[d]*Department of Chemistry, University of Oxford, Mansfield Rd, Oxford, OX1 3TA, United Kingdom*

[e]*Chemistry Department, Faculty of Science, Tanta University, Tanta, El Gharbia, 31527, Egypt*

[f]*Department of Physics, Chemistry and Biology (IFM), Linköping University, 581 83 Linköping, Sweden*

*Email: hab60@cam.ac.uk

Table of Contents

Table of Contents 2
S1. General Methods 3
S2. Synthetic Procedures and Characterisation 6
S3. NMR Spectra 20
S4. X-Ray Crystallography 40
S5. Thermogravimetric Analysis 43
S6. Cyclic Voltammetry 44
S7. Photophysical Characterisation 45
S8. Computational Details 63
S9. OLED Devices 73
Supporting References 79

S1. General Methods

All synthetic procedures were carried out in the Yusuf Hamied Department of Chemistry at the University of Cambridge. All reagents and solvents (≥95% purity) were purchased from commercial sources (Sigma-Aldrich, Acros Organics, Alfa Aesar, Thermo Fisher Scientific, Merck Life Science, Fluorochem, Apollo Scientific) and used as received without further purification, unless stated otherwise. Non-anhydrous solvents *n*-hexane, petroleum ether (40 - 60 °C), dichloromethane (DCM) and toluene were distilled in the Yusuf Hamied Department of Chemistry at the University of Cambridge before use. Where necessary, glassware was dried in an oven set to 215 °C before use. All reactions requiring an inert atmosphere were carried out under an argon atmosphere. All reactions were stirred magnetically, and where necessary, were heated using a silicone oil bath on a hot plate stirrer. For each procedure, analytical thin-layer chromatography (TLC) was carried out using a silica gel 60 matrix on aluminium supports (with 254 nm fluorescent indicator) from Sigma-Aldrich and visualised using UV light (254, 365 nm). Silica and celite 'plugs' refer to the compacted material with a depth of 1 – 2 inches. Column chromatography was carried out using a Biotage® Isolera™ Four System and Biotage® SNAP/Sfär pre-packed 60 μm silica D gel flash columns (sizes: 25 g, 50 g, 100 g, 200 g) with non-anhydrous solvents: *n*-hexane, petroleum ether (40 - 60 °C), dichloromethane (DCM), and tetrahydrofuran (THF). Each crude product was dry loaded onto the column using silica 60 gel. ^{1}H NMR and ^{13}C NMR spectra were recorded at the stated temperature in the stated solvent using a Bruker Avance II+ 700 MHz spectrometer. All NMR spectra were processed using MestReNova v14.2.0-26256 (Mestrelab Research, S.L.), and were baseline corrected, and phase corrected, where necessary. For ^{1}H NMR, the residual protic solvent was used as an internal reference ($CHCl_3$: 7.26 ppm singlet, $CHDCl_2$: 5.32 ppm triplet, DMSO-d_5: 2.50 ppm quintet, C_6HD_5: 7.16 ppm singlet). For ^{13}C NMR, the deuterated solvent was used as an internal reference ($CDCl_3$: 77.2 ppm triplet, CD_2Cl_2: 54.0 ppm quintet,

DMSO-d_6: 39.5 ppm septet, C_6HD_5: 128.1 ppm triplet). ^{1}H NMR chemical shifts are reported in parts per million (ppm) to the nearest 0.01 ppm, while ^{13}C NMR chemical shifts are reported in parts per million (ppm) to the nearest 0.1 ppm. The multiplicity of the ^{1}H NMR spectra peaks are given as : s, singlet, brs, broad singlet, d, doublet, dd, doublet of doublets, ddd, doublet of doublet of doublets, t, triplet, m, multiplet. The proton-proton scalar coupling constants (*J*) are measured in Hertz and reported to the nearest 0.5 Hz. All ambiguous splitting patterns are assigned to multiplets. The number of protons that each resonance in the ^{1}H NMR spectra correspond to are given. ^{1}H-^{1}H-ROESY spectra were measured on a Bruker Avance II+ 700 MHz spectrometer by Dr Pete Gierth from the NMR team in the Yusuf Hamied Department of Chemistry at the University of Cambridge. All accurate mass measurements were performed by the mass spectrometry team in the Yusuf Hamied Department of Chemistry at the University of Cambridge using either an Agilent 1260 Infinity LC System coupled to an Agilent 6230 time-of-flight (TOF) LC/MS System (Agilent Technologies, Santa Clara, CA), a Waters Xevo G2-S, a Waters Synapt G2-Si, a Q Exactive Orbitrap, a Waters Xevo TQD, a Waters SQD2, or a Waters Vion IMS Qtof. The Agilent 1260 LC module was equipped with a variable wavelength (VWD) detector along with a binary solvent pump and an autosampler. Chromatographic separations were performed using a Poroshell 120 EC-C18 reverse phase C18 column 3.0 mm × 50 mm; 2.7 μm (Agilent Technologies, Santa Clara, CA). The solvent pump was connected to a gradient binary solvent system: A, H_2O and B, MeCN, and 0.1 % formic acid (v/v) or 10 mM ammonium formate modifiers were added after column separation for positive and negative polarity runs, respectively. The mobile phase was programmed to run as follows: 0–1.5 min, 95% A and 5% B to 0% A and 100% B; 3-6 min, 100% B; and return to the starting mobile phase composition with re-equilibration. The injection volume was 1 μL for each of the sample solutions, followed by needle wash. The column temperature was maintained at 40 °C. The elution was run at a flow rate of

0.4 mL/min. UV spectra were monitored at 254 nm. The ionization source was Agilent Jet Stream, with electrospray ionization (ESI) in either positive or negative modes for acquisition of mass spectra. In some cases, atmospheric solids analysis probe (ASAP) ionisation in either positive or negative modes was required to acquire mass spectra. Here, solid samples were introduced directly using a probe. Nitrogen was supplied from a centralised reservoir and used as the drying and nebulizer gas. Other MS instrumental conditions are as follows: drying gas temperature and flow rate were 325°C and 10.0 L/min, respectively; nebulizer pressure was 40 psi; sheath gas temperature and flow rate were 400 °C and 12.0 L/min, respectively; capillary, nozzle and fragmentor were set to 4000 V, 500V and 200 V respectively; skimmer was 65.0; Oct 1 RF was 750. The instrument state was set to extended dynamic range mode (2 GHz). Data collection and integration were performed using MassHunter WalkUp Console software (version 4.1 Build 4.1.313). The data was collected in the range of 100 and 3000 m/z. Data were stored in both centroid and profile formats during acquisition. Two independent reference lock-mass ions, purine ($C_5H_4N_4$) at *m/z* 121.0509 and HP-921 (hexakis-(2,2,3,3-tetrafluoropentoxy)phosphazene) ($C_{18}H_{18}O_6N_3P_3F_{24}$) at *m/z* 922.0098, were employed using a low flow of the calibrants solution (calibrant solution A, Agilent Technologies) to ensure mass accuracy and reproducibility. All reported mass values have errors less than 5 ppm with respect to their calculated value. All elemental analyses were conducted by Dr Nigel Howard of the

microanalysis team at the in the Yusuf Hamied Department of Chemistry at the University of Cambridge. Carbon, hydrogen, and nitrogen content were determined by combustion analysis. High-performance liquid chromatography (HPLC) purity analyses were performed using an Agilent 1260 Infinity II system, fitted with a Phenomenex 'Lux 5 µm i-Cellulose-5 LC Column 250 x 21.2 mm, AXIATM Packed' column. All runs were at a flow rate of 15 mL min^{-1}.

S2. Synthetic Procedures and Characterisation

Synthesis of OMeBOBO

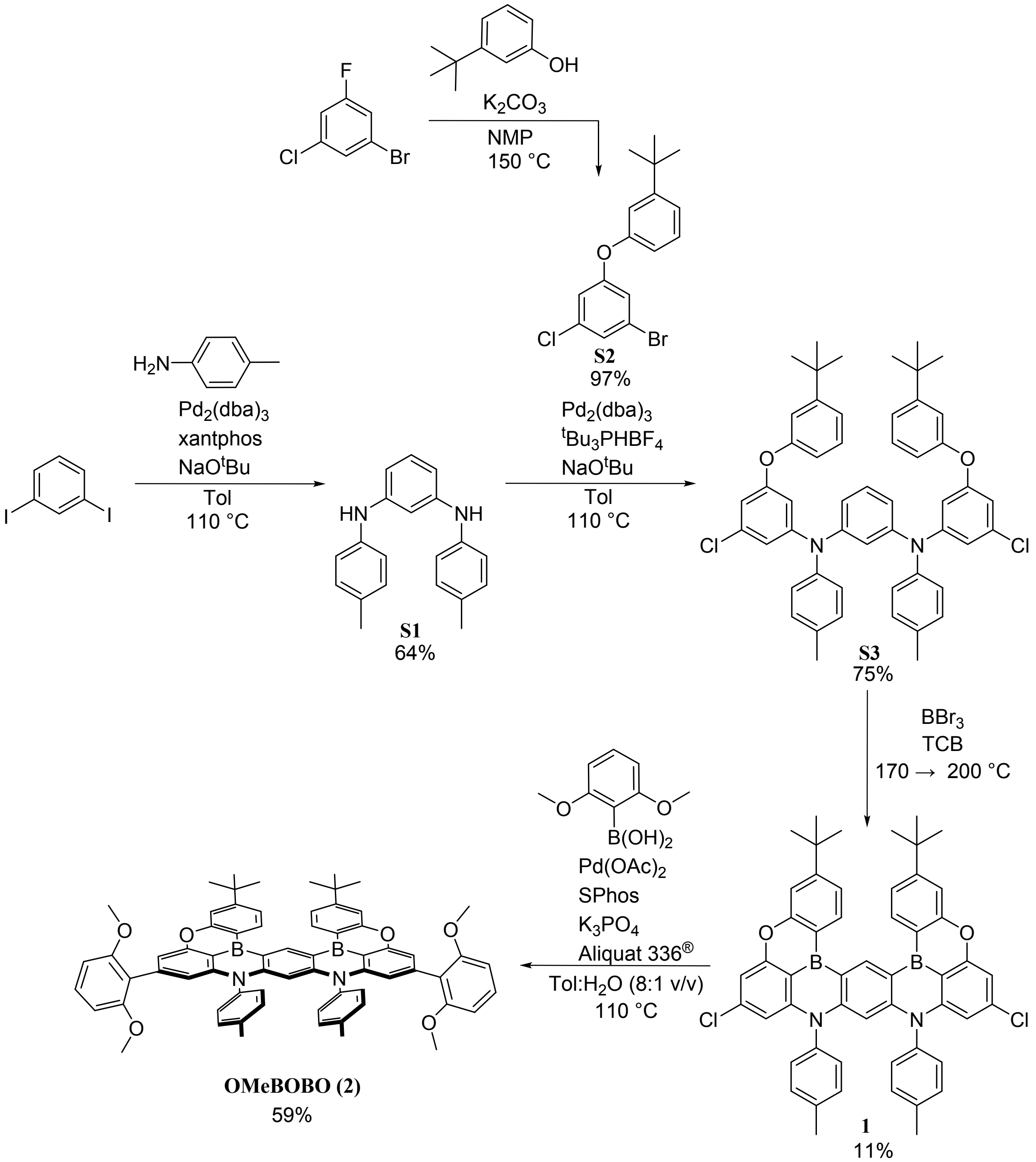

Scheme S1. The synthetic route towards **OMeBOBO.**

N^1,N^3-di-*p*-tolylbenzene-1,3-diamine (Compound S1)

1,3-Diiodobenzene (4.99 g, 15.1 mmol, 1.00 *eq.*), *p*-toluidine (4.89 g, 45.7 mmol, 3.02 *eq.*), tris(dibenzylideneacetone)dipalladium(0) ($Pd_2(dba)_3$) (346 mg, 2.50 mol%), xantphos (881 mg, 10.0 mol%), and sodium *tert*-butoxide (NaO^tBu) (4.37 g, 45.5 mmol, 3.01 *eq.*) were added to an oven-dried 100 mL pressure flask equipped with a stirrer bar under argon. The flask was evacuated and backfilled with argon five times before approximately 35 mL of anhydrous toluene was added. The flask was sealed under argon and the reaction mixture was heated at 110 °C overnight before being allowed to cool to room temperature. The reaction mixture was filtered through a celite plug (eluent: toluene) and 1 M aqueous hydrochloric acid (HCl) solution was added to the filtrate. The resulting aqueous layer was extracted three times with toluene before the combined organic layers were washed with brine, dried over $MgSO_4$, and concentrated *in vacuo*. The resulting crude product was purified *via* column chromatography (silica gel, eluent: gradient, 0 – 30 % DCM in petroleum ether (40 – 60 °C) spiked with Et_3N). The product-containing fractions were combined and concentrated *in vacuo* and then concentrated *in vacuo* from petroleum ether (40 – 60 °C). The resulting solid was filtered under reduced pressure from petroleum ether (40 – 60 °C) to give the final product as white needles (2.79 g, 9.67 mmol, 64% yield).

^{1}H NMR (700 MHz, CD_2Cl_2, 298 K) δ / ppm = 7.10 – 7.05 (m, 5H,), 6.99 (d, J = 8.5 Hz, 4H,), 6.65 (t, J = 2.0 Hz, 1H), 6.51 (dd, J = 8.0, 2.0 Hz, 2H), 5.66 (brs, 2H), 2.29 (s, 6H);

^{13}C NMR (176 MHz, CD_2Cl_2, 298 K) δ / ppm = 145.7, 140.8, 131.5, 130.6, 130.3, 119.5, 109.2, 104.9, 20.9;

HRMS (ESI+): calculated for $C_{20}H_{21}N_2^+$ m/z: 289.1699, found 289.1692 (error = -2.65 ppm).

The data are consistent with those reported in the literature.[1]

1-bromo-3-(3-(*tert*-butyl)phenoxy)-5-chlorobenzene (Compound S2)

3-*tert*-Butylphenol (7.88 g, 52.5 mmol, 1.10 *eq.*), potassium carbonate (K_2CO_3) (9.89 g, 71.6 mmol, 1.50 *eq.*) and approximately 200 mL of anhydrous N-methylpyrrolidone (NMP) were combined in an oven-dried 500 mL triple-neck round-bottomed flask equipped with a stirrer bar and a condenser under argon. This mixture was stirred for 30 minutes before the addition of 1-bromo-3-chloro-5-fluorobenzene (10.0 g, 47.7 mmol, 1.00 *eq.*). The resulting reaction mixture was heated at 150 °C overnight and then cooled to room temperature before being diluted with petroleum ether (40 – 60 °C) and distilled water. The resulting aqueous layer was extracted with petroleum ether (40 – 60 °C) three times and the combined organic layers were washed with distilled water three times before being dried over $MgSO_4$ and concentrated *in vacuo*. The resulting oil was redissolved in petroleum ether (40 – 60 °C) and filtered through a silica plug (eluent: petroleum ether (40 – 60 °C)). The filtrate was concentrated *in vacuo* to yield the title product as a colourless oil (15.8 g, 46.4 mmol, 97% yield).

^{1}H NMR (700 MHz, CD_2Cl_2, 298 K) δ / ppm = 7.32 (t, J = 8.0 Hz, 1H), 7.25 (ddd, J = 8.0, 2.0, 1.0 Hz, 1H), 7.23 (t, J = 2.0 Hz, 1H), 7.10 (t, J = 2.0 Hz, 1H), 7.03 (t, J = 2.0 Hz, 1H), 6.92 (t, J = 2.0 Hz, 1H), 6.83 (ddd, J = 8.0, 2.5, 1.0 Hz, 1H), 1.31 (s, 9H);

^{13}C NMR (176 MHz, CD_2Cl_2, 298 K) δ / ppm = 160.0, 155.7, 154.7, 136.2, 130.1, 126.0, 123.4, 122.5, 119.9, 117.9, 117.5, 117.3, 35.3, 31.5;

HRMS (ASAP+): calculated for $C_{16}H_{17}BrClO^+$ m/z: 339.0151, found 339.0145 (error = - 1.8 ppm).

N^1,N^3-bis(3-(3-(*tert*-butyl)phenoxy)-5-chlorophenyl)-N^1,N^3-di-*p*-tolylbenzene-1,3-diamine (Compound S3)

Compound **S1** (2.75 g, 9.54 mmol, 1.00 *eq.*), tris(dibenzylideneacetone)dipalladium(0) ($Pd_2(dba)_3$) (266 mg, 0.291 mmol, 3.05 mol%), tri-tert-butylphosphonium tetrafluoroborate ($^tBu_3P$•HBF_4) (338 mg, 1.16 mmol, 12.2 mol%), and sodium tert-butoxide (NaO^tBu) (2.23 g, 23.2 mmol, 2.43 *eq.*) were combined in an oven-dried 100 mL pressure flask equipped with a stirrer bar under argon. The flask was evacuated and backfilled with argon five times before approximately 48 mL of anhydrous toluene followed by compound **S2** (10.2 g, 30.0 mmol, 3.15 *eq.*) were injected into the flask. The flask was sealed under argon and heated at 110 °C overnight. The reaction mixture was then allowed to cool to room temperature before being passed through a celite plug (eluent: toluene). The resulting filtrate was washed with distilled water three times before it was dried over $MgSO_4$ and concentrated *in vacuo*. The resulting crude product was purified *via* column chromatography (silica gel, eluent: gradient, 0 – 25 % DCM in petroleum ether (40 – 60 °C)). The product-containing fractions were combined and concentrated *in vacuo* and then concentrated *in vacuo* from petroleum ether (40 – 60 °C)) to yield the product as a white crystalline powder (5.82 g, 7.22 mmol, 75% yield).

^{1}H NMR (700 MHz, CD_2Cl_2, 298 K) δ / ppm = 7.24 (t, J = 8.0 Hz, 2H), 7.18 – 7.10 (m, 3H), 7.09 (d, J = 8.0 Hz, 4H), 7.02 (t, J = 2.0 Hz, 2H), 6.97 (d, J = 8.5 Hz, 4H), 6.80 (t, J = 2.0 Hz, 1H), 6.76 (ddd, J = 8.0, 2.5, 1.0 Hz, 2H), 6.71 (dd, J = 8.0, 2.0 Hz, 2H), 6.65 (t, J = 2.0 Hz, 2H), 6.50 (t, J = 2.0 Hz, 2H), 6.45 (t, J = 2.0 Hz, 2H), 2.29 (s, 6H), 1.27 (s, 18H);

^{13}C NMR (176 MHz, CD_2Cl_2, 298 K) δ / ppm = 159.5, 156.4, 154.3, 150.5, 148.5, 144.4, 135.6, 134.8, 130.7, 130.6, 129.8, 126.1, 121.6, 121.2, 120.0, 117.3, 116.8, 116.6, 111.7, 110.7, 35.2, 31.5, 21.1;

HRMS (ASAP+): calculated for $C_{52}H_{51}Cl_2N_2O_2^+$ m/z: 805.3328, found 805.3321 (error = -0.9 ppm).

Compound 1

Compound **S3** (3.63 g, 4.50 mmol, 1.00 *eq.*) was added to an oven-dried 120 mL pressure flask equipped with a stirrer bar under argon. The flask was evacuated and backfilled with argon three times before approximately 45 mL of anhydrous 1,2,4- trichlorobenzene (TCB) was added to give a solution. Boron tribromide (BBr_3) (6.94 mL, 72.0 mmol, 16.0 *eq.*) was slowly injected into the flask before it was sealed under argon and heated at 170 °C overnight and then heated at 200 °C for a second night. The reaction mixture was allowed to cool to room temperature before being quenched with saturated aqueous sodium hydrogen carbonate ($NaHCO_3$) solution and filtered through a celite plug (eluent: DCM, distilled water). The aqueous layer of the resulting filtrate was extracted with DCM three times, and the combined organic layers were washed with brine before being dried over $MgSO_4$ and concentrated *in vacuo*. The crude product was purified *via* column chromatography (silica gel, eluent: gradient, 0 – 25 % DCM in petroleum ether (40 – 60 °C)). A solid crystallised from some of the product-containing fractions and this was isolated by filtration under reduced pressure to give the final product as a light-yellow powder (185 mg, 0.225 mmol, 5% yield). The filtrate and remaining product-containing fractions were concentrated *in vacuo* and then concentrated *in vacuo* from petroleum ether (40 – 60 °C)). The resulting solid was recrystallised from *n*-hexane and chloroform to give a second portion of the final product (230 mg, 0.280 mmol, 6% yield, 11% total yield).

^{1}H NMR (700 MHz, CD_2Cl_2, 298 K) δ / ppm = 10.41 (s, 1H), 8.98 (d, J = 8.0 Hz, 2H), 7.65 (dd, J = 8.0, 2.0 Hz, 2H), 7.59 (d, J = 2.0 Hz, 2H), 7.29 (d, J = 8.0 Hz, 4H), 7.22 (d, J = 1.5 Hz, 2H), 6.97 (d, J = 8.0 Hz, 4H), 6.43 (d, J = 1.5 Hz, 2H), 5.98 (s, 1H), 2.53 (s, 6H), 1.51 (s, 18H);

^{13}C NMR (176 MHz, CD_2Cl_2, 298 K) δ / ppm = 160.5, 159.1, 157.7, 151.3, 147.8, 145.2, 139.4, 138.5, 134.6, 132.0, 129.7, 127.8, 121.7, 115.4, 111.6, 110.0, 105.3, 35.7, 31.5, 21.8;

HRMS (ESI+): calculated for $C_{52}H_{45}B_2Cl_2N_2O_2^+$ m/z: 821.3044, found 821.3049 (error = 0.6 ppm).

Note: carbon environments directly attached to boron nuclei are not always observable.[2]

OMeBOBO (Compound 2)

Compound **1** (224 mg, 0.273 mmol, 1.00 *eq.*), 2,6-dimethoxyphenylboronic acid (252 mg, 1.39 mmol, 5.09 *eq.*), SPhos (20.0 mg, 17.8 mol%), and palladium acetate ($Pd(OAc)_2$) (5.6 mg, 9.1 mol%) were added to an oven-dried 120 mL pressure flask equipped with a stirrer bar under argon. Potassium phosphate (K_3PO_4) (294 mg, 1.39 mmol, 5.09 *eq.*) was added to a 20 mL microwave vial and both vessels were evacuated and backfilled with argon five times. Anhydrous toluene (*ca.* 46 mL) was added to the pressure flask while distilled water (*ca.* 6 mL) was added to the microwave vial, and the resulting solutions were degassed by argon bubbling for approximately 10 minutes. The basic solution and approximately five drops of Aliquat® 336 was injected into the toluene mixture before the flask was sealed under argon and heated at 110 °C overnight using a pre-heated silicone oil bath. The reaction mixture was allowed to cool to room temperature before being acidified with 1 M aqueous hydrochloric acid (HCl) solution and diluted with toluene. The resulting aqueous layer was extracted with toluene three times, and the combined organic layers were dried over $MgSO_4$ and concentrated *in vacuo*. The resulting solid was subject to column chromatography (silica gel, eluent: gradient, 0 – 50 % DCM in *n*-hexane). The product-containing fractions were combined and concentrated *in vacuo* and then concentrated *in vacuo* from *n*-hexane. The resulting solid was filtered under reduced pressure from methanol to give the crude product as a yellow powder. The final product was recrystallised from a mixture of *n*-hexane and chloroform to give a yellow powder (167 mg, 0.160 mmol, 59% yield).

^{1}H NMR (700 MHz, CD_2Cl_2, 298 K) δ / ppm = 10.46 (s, 1H), 9.04 (d, J = 8.0 Hz, 2H), 7.64 (dd, J = 8.0, 2.0 Hz, 2H), 7.59 (d, J = 2.0 Hz, 2H), 7.25 (t, J = 8.5 Hz, 2H), 7.22 (d, J = 7.5 Hz, 4H), 7.05 – 7.00 (m, 6H, k), 6.61 (d, J = 8.5 Hz, 4H), 6.28 (d, J = 1.0 Hz, 2H), 5.99 (s, 1H), 3.67 (s, 12H), 2.45 (s, 6H), 1.53 (s, 18H);

^{13}C NMR (176 MHz, CD_2Cl_2, 298 K) δ / ppm = 160.9, 158.1, 158.1, 157.2, 151.5, 146.5, 145.2, 140.3, 139.3, 138.7, 134.7, 131.6, 130.0, 129.6, 121.1, 120.0, 115.3, 111.7, 109.2, 104.8, 104.5, 56.4, 35.7, 31.6, 21.7;

HRMS (ESI+): calculated for $C_{69}H_{66}B_2N_2O_6^+$ m/z: 1040.5107, found 1040.5090 (error = -1.6 ppm);

Anal. Cald. for $C_{69}H_{66}B_2N_2O_6 \cdot 0.1CHCl_3$: C – 78.8%, H– 6.33%, N – 2.7%, found: C – 78.6%, H – 5.95%, N – 3.0% (average of two runs)

Note: carbon environments directly attached to boron nuclei are not always observable.[2]

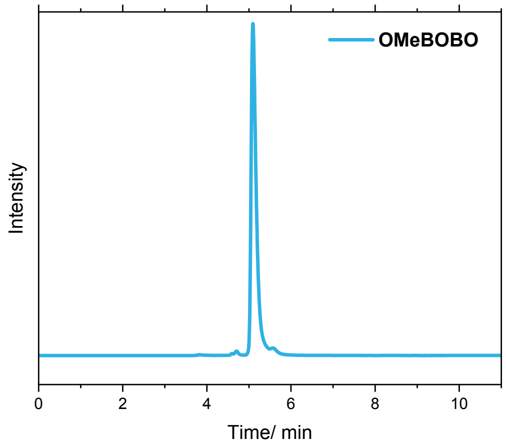

Figure S1. HPLC chromatogram of **OMeBOBO**. 0.1 mL of **OMeBOBO** dissolved in a chloroform and n-hexane mixture (3:2) (~ 4 mg mL^{-1}) was injected. 60% chloroform in n-hexane (HPLC grade) was used as the mobile phase. Retenton time (t_R) = 5.10 min. Purity (440 nm) = 97%

Synthesis of EnBOBO

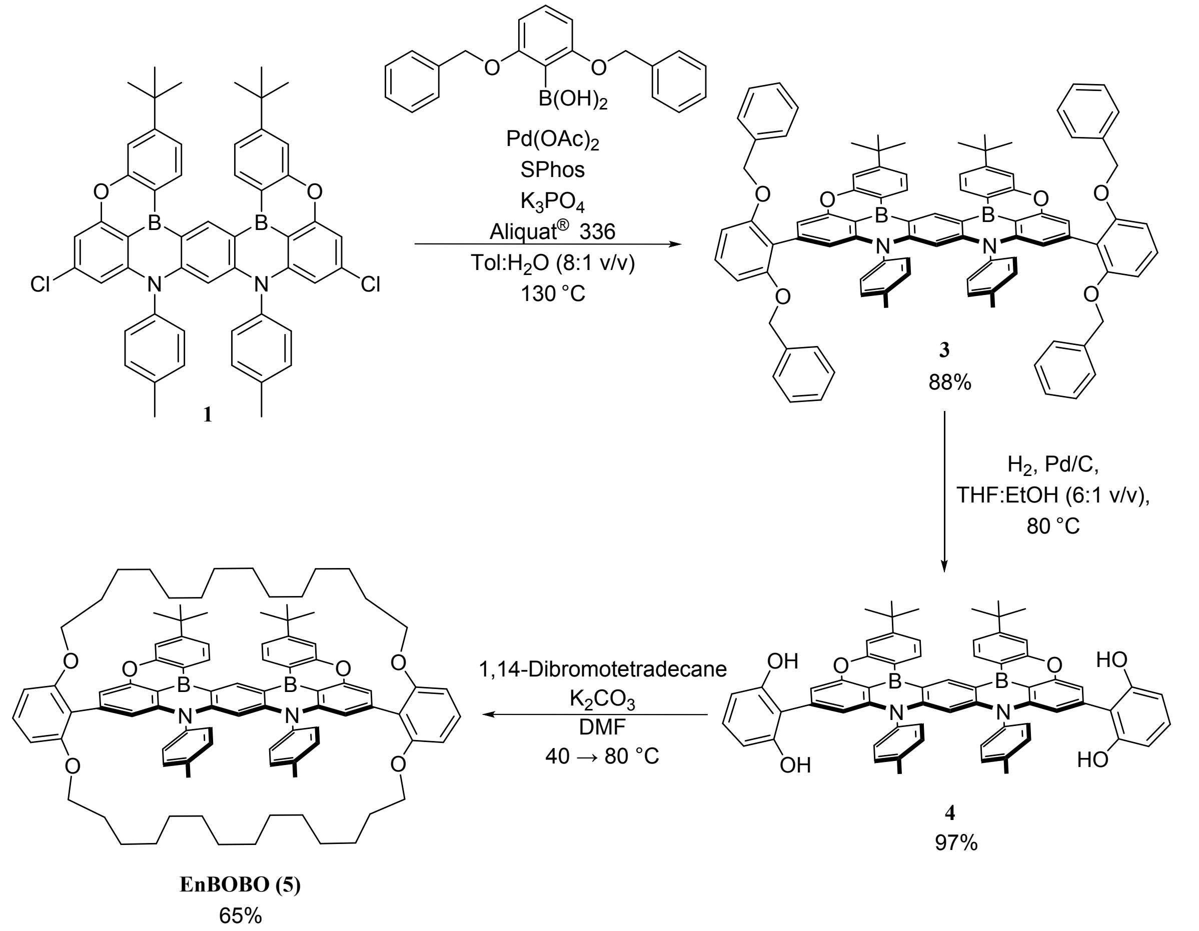

Scheme S2. The synthetic route towards **EnBOBO.**

(2,6-bis(benzyloxy)phenyl)boronic acid was synthesised according to literature procedures.[3]

^{1}H NMR (700 MHz, $CDCl_3$, 298 K) δ / ppm = 7.44 – 7.34 (m, 11H), 7.20 (s, 2H), 6.71 (d, J = 8.5 Hz, 2H), 5.15 (s, 4H);

^{13}C NMR (176 MHz, $CDCl_3$, 298 K) δ / ppm = 164.9, 135.7, 133.1, 129.1, 128.8, 127.9, 106.1, 71.5;

HRMS (ESI+): calculated for $C_{20}H_{20}O_4B^+$ m/z: 335.1449, found 335.1441 (error = -2.4 ppm).

The data are consistent with those reported in the literature.[4]

Compound 3

Compound **1** (175 mg, 0.213 mmol, 1.00 *eq.*), (2,6-bis(benzyloxy)phenyl)boronic acid (363 mg, 1.08 mmol, 5.09 *eq.*), SPhos (16.3 mg, 18.6 mol%), and palladium acetate ($Pd(OAc)_2$) (4.5 mg, 9.4 mol%) were added to an oven-dried 120 mL pressure flask equipped with a stirrer bar under argon. Potassium phosphate (K_3PO_4) (232 mg, 1.09 mmol, 5.13 *eq.*) was added to a 20 mL microwave vial and both vessels were evacuated and backfilled with argon five times. Anhydrous toluene (*ca.* 38 mL) and approximately five drops of Aliquat® 336 were added to the pressure flask while distilled water (*ca.* 5 mL) was added to the microwave vial, and the resulting solutions were degassed by argon bubbling for approximately 10 minutes. The basic solution was injected into the toluene mixture before this flask was sealed under argon and heated at 130 °C overnight using a pre-heated silicone oil bath. The reaction mixture was allowed to cool to room temperature before being acidified with 1 M aqueous hydrochloric acid (HCl) solution and diluted with toluene. The resulting aqueous layer was extracted with toluene two times, and the combined organic layers were dried over $MgSO_4$ and concentrated *in vacuo*. The resulting solid was subject to column chromatography (silica gel, eluent: gradient, 0 – 50 % DCM in *n*-hexane). The product-containing fractions were combined and concentrated *in vacuo* and then concentrated *in vacuo* from *n*-hexane to yield a solid which was filtered from methanol under reduced pressure to yield the final product as a yellow powder (251 mg, 0.189 mmol, 88% yield).

^{1}H NMR (700 MHz, CD_2Cl_2, 298 K) δ / ppm = 10.47 (s, 1H), 9.05 (d, J = 8.0 Hz, 2H), 7.65 (dd, J = 8.0, 2.0 Hz, 2H), 7.62 (d, J = 2.0 Hz, 2H), 7.27 – 7.11 (m, 28H), 6.92 (d, J = 8.0 Hz, 4H), 6.61 (d, J = 8.5 Hz, 4H), 6.41 (d, J = 1.0 Hz, 2H), 5.92 (s, 1H), 4.98 (s, 8H), 2.44 (s, 6H), 1.53 (s, 18H);

^{13}C NMR (176 MHz, CD_2Cl_2, 298 K) δ / ppm = 161.0, 158.2, 157.2, 157.1, 151.5, 146.6, 145.1, 140.4, 139.3, 138.6, 137.9, 134.7, 131.7, 130.1, 129.4, 128.9, 128.1, 127.3, 121.5,

121.1, 115.4, 111.4, 109.5, 106.9, 104.5, 70.9, 35.7, 31.6, 21.8;

HRMS (ESI+): calculated for $C_{92}H_{79}B_2N_2O_6^+$ m/z: 1329.6119, found 1329.6139 (error = 1.6 ppm).

Note: carbon environments directly attached to boron nuclei are not always observable.[2]

Compound 4

Compound **3** (238 mg, 0.179 mmol, 1.00 *eq.*) and 10 wt% palladium on carbon (Pd/C) (77 mg) were added to an oven-dried 120 mL pressure flask equipped with a stirrer bar under argon. Anhydrous THF (*ca.* 24 mL) and ethanol (*ca.* 4 mL) were added, and the resulting mixture was bubbled with hydrogen gas for approximately twenty minutes before the head space of the flask was filled with hydrogen gas. The flask was sealed under this hydrogen atmosphere and heated overnight at 80 °C. The reaction mixture was allowed to cool to room temperature before being filtered through a celite plug (eluent: THF). The resulting filtrate was concentrated *in vacuo* to yield the crude product, which was then subject to column chromatography (silica gel, eluent: gradient, 0 – 50 % THF in *n*-hexane). The product-containing fractions were combined and concentrated *in vacuo* and filtered under reduced pressure from petroleum ether (40 – 60 °C) to obtain the product as a dark yellow powder (175 mg, 0.181 mmol, 97 % yield).

^{1}H NMR (700 MHz, DMSO-d_6, 298 K) δ / ppm = 10.33 (s, 1H), 9.12 (s, 4H), 8.92 (d, J = 8.0 Hz, 2H), 7.74 (dd, J = 8.0, 2.0 Hz, 2H), 7.60 (d, J = 2.0 Hz, 2H), 7.28 (d, J = 8.5 Hz, 4H), 7.06 – 7.00 (m, 6H), 6.89 (t, J = 8.0 Hz, 2H), 6.33 (d, J = 8.0 Hz, 4H), 6.29 (d, J = 1.0 Hz, 2H), 5.93 (s, 1H), 2.45 (s, 6H), 1.49 (s, 18H);

^{13}C NMR (176 MHz, DMSO-d_6, 298 K) δ / ppm = 159.7, 156.7, 156.6, 155.4, 150.5, 145.2, 141.1, 138.0, 137.9, 133.5, 131.2, 129.1, 128.6, 121.0, 120.0, 115.7, 114.7, 111.2, 108.9, 106.6, 103.7, 35.0, 31.0, 20.9;

HRMS (ESI+): calculated for $C_{64}H_{55}B_2N_2O_6^+$ m/z: 969.4246, found 969.4216 (error = - 3.1 ppm).

Note: carbon environments directly attached to boron nuclei are not always observable.[2]

EnBOBO (Compound 5)

1,14-dibromotetradecane (125 mg, 0.351 mmol, 2.05 *eq.*) was dissolved in approximately 43 mL of anhydrous DMF in an oven-dried 100 mL pressure flask equipped with a stirrer bar under argon. Compound **4** (166 mg, 0.171 mmol, 1.00 *eq.*) and potassium carbonate (K_2CO_3) (122 mg, 0.883 mmol, 5.16 *eq.*) were added. The reaction vessel was sealed under argon and heated at 40 °C for approximately 24 hours and then at 80 °C for approximately 48 hours. The reaction mixture was cooled to room temperature before being acidified with 1 M aqueous hydrochloric acid (HCl) solution and diluted with DCM. The aqueous layer was extracted with DCM three times, and the combined organic layers were washed with the 1 M aqueous HCl solution five times before being dried over $MgSO_4$ and concentrated *in vacuo*. The crude product was subject to column chromatography (silica gel, eluent: gradient, 0 – 50 % DCM in petroleum ether (40 – 60 °C)). The product-containing fractions were combined and concentrated *in vacuo* and then concentrated *in vacuo* from petroleum ether (40 – 60 °C) before the resulting solid was filtered under reduced pressure from methanol to give the final product as a yellow powder (153 mg, 0.111 mmol, 65% yield).

^{1}H NMR (700 MHz, C_6D_6, 298 K) δ / ppm = 10.99 (s, 1H), 9.35 (d, J = 8.0 Hz, 2H), 7.65 (d, J = 2.0 Hz, 2H), 7.60 (d, J = 1.0 Hz, 2H), 7.49 (dd, J = 8.0, 2.0 Hz, 2H), 7.29 (d, J = 8.0 Hz, 4H), 7.01 (t, J = 8.5 Hz, 2H), 6.88 (d, J = 8.0 Hz, 4H), 6.72 (d, J = 1.0 Hz, 2H), 6.52 (d, J = 8.5 Hz, 4H), 6.25 (s, 1H), 3.71 – 3.67 (m, 4H), 3.49 – 3.44 (m, 4H), 1.97 (s, 6H), 1.42 – 1.34 (m, 4H), 1.33 – 1.26 (m, 4H), 1.25 (s, 18H), 1.10 – 0.97 (m, 8H), 0.93 – 0.80 (m, 32H);

^{13}C NMR (176 MHz, C_6D_6, 298 K) δ / ppm = 161.2, 158.6, 158.2, 156.3, 151.6, 146.6, 141.6, 139.9, 137.7, 134.9, 131.4, 130.0, 129.1, 128.6, 125.6, 121.0, 115.6, 111.3, 110.1, 109.9, 104.4, 71.2, 35.0, 31.8, 31.6, 31.4, 31.2, 31.1, 30.6, 27.7, 21.2;

HRMS (ESI+): calculated for $C_{92}H_{107}B_2N_2O_6^+$ m/z: 1357.8315, found 1357.8367 (error = 3.8 ppm).

Anal. Cald. for $C_{92}H_{106}B_2N_2O_6$: C – 81.4% H– 7.85% N – 2.1%, found: C – 80.9%, H - 7.95%, N – 2.3% (average of two runs).

Note: carbon environments directly attached to boron nuclei are not always observable.[2]

Synthesis of Sensitisers

TDBA-PAS was synthesised according to literature procedures.[5]

^{1}H NMR (700 MHz, $CDCl_3$, 298 K) δ / ppm = 8.78 (d, J = 2.5 Hz, 2H), 7.78 (dd, J = 8.5, 2.5 Hz, 2H), 7.66 - 7.61 (m, 4H), 7.61 (dd, J = 7.5, 2.0 Hz, 2H), 7.47 (d, J = 8.5 Hz, 2H), 7.42 – 7.35 (m, 6H), 7.21 (ddd, J = 8.5, 7.0, 2.0 Hz, 2H), 7.09 (s, 2H), 7.00 (t, J = 7.0 Hz, 2H), 6.73 (d, J = 8.5 Hz, 2H), 1.51 (s, 18H);

^{13}C NMR (176 MHz, $CDCl_3$, 298 K) δ / ppm = 159.5, 158.9, 149.9, 149.7, 145.4, 136.3, 136.0, 134.9, 131.7, 130.5, 130.4, 129.8, 128.1, 121.1, 119.0, 118.3, 118.1, 109.2, 34.7, 31.7;

HRMS (ESI+): calculated for $C_{50}H_{45}BNO_2Si^+$ m/z: 730.3307, found 730.3295 (error = -1.7 ppm).

Anal. Cald. for $C_{50}H_{44}BNO_2Si{\cdot}0.2CHCl_3$: C – 80.0% H– 5.91% N – 1.9%, found: C – 79.8%, H – 5.93%, N – 1.8% (average of two runs)

The data are consistent with those reported in the literature.[5]

Note: carbon environments directly attached to boron nuclei are not always observable.[2]

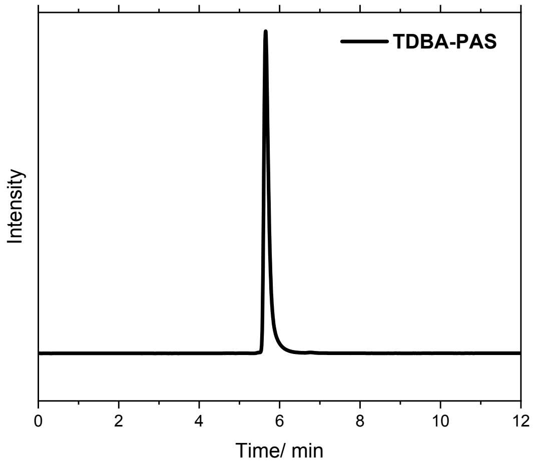

Figure S2. HPLC chromatogram of TDBA-PAS. 0.1 mL of TDBA-PAS dissolved in a chloroform and *n*-hexane mixture (1:4) (~ 4 mg mL^{-1}) was injected. 20% chloroform in *n*-hexane (HPLC grade) was used as the mobile phase. Rention time (t_R) = 5.65 min. Purity (375 nm) = 100%

TDBA-SPQ was synthesised according to literature procedures.[6]

^{1}H NMR (700 MHz, $CDCl_3$, 298 K) δ / ppm = 8.82 (d, J = 2.5 Hz, 2H), 7.83 (dd, J = 8.5, 2.5 Hz, 2H), 7.71 (t, J = 7.5 Hz, 2H), 7.60 – 7.53 (m, 3H), 7.51 – 7.43 (m, 2H), 7.36 (s, 2H), 7.23 – 7.18 (m, 4H), 6.93 – 6.87 (m, 4H), 6.82 – 6.74 (m, 4H), 6.43 (d, J = 8.0 Hz, 2H), 6.29 (d, J = 7.5 Hz, 2H), 1.53 (s, 18H);

^{13}C NMR (176 MHz, $CDCl_3$, 298 K) δ / ppm = 159.7, 158.9, 147.2, 145.6, 141.4, 138.7, 138.2, 133.0, 132.9, 132.1, 131.98, 131.95, 131.5, 131.3, 130.5, 128.6, 126.8, 126.7, 121.2, 121.0, 118.2, 114.3, 114.1, 111.3, 34.8, 31.7;

HRMS (ESI+): calculated for $C_{57}H_{47}BN_2O_2^+$ m/z: 802.3725, found 802.3708 (error = -2.1 ppm).

Anal. Cald. for $C_{57}H_{47}BN_2O_2$: C – 85.3% H– 5.90% N – 3.5%, found: C – 85.2%, H – 5.81%, N – 3.4% (average of two runs).

The data are consistent with those reported in the literature.[6]

Note: carbon environments directly attached to boron nuclei are not always observable.[2]

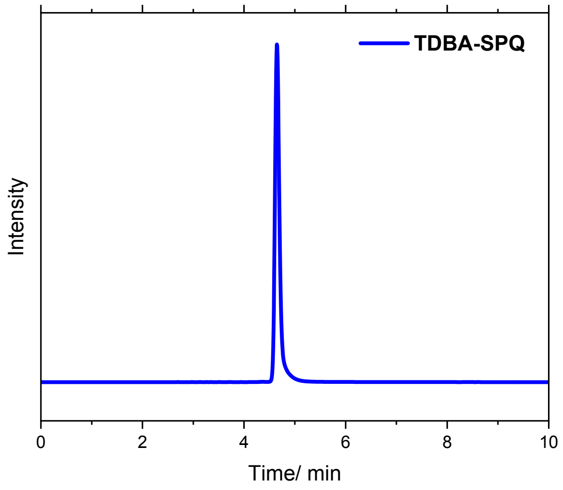

Figure S3. HPLC chromatogram of TDBA-SPQ. 0.1 mL of TDBA-SPQ dissolved in a chloroform and *n*-hexane mixture (3:7) was injected (~ 4 mg mL^{-1}). 30% chloroform in *n*-hexane (HPLC grade) was used as the mobile phase. Retention time (t_R) = 4.65 min. Purity (375 nm) = 100%

S3. NMR Spectra

N[1],*N*[3]-di-*p*-tolylbenzene-1,3-diamine (Compound S1)

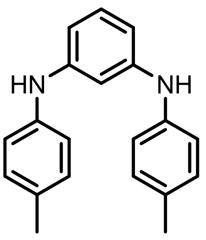

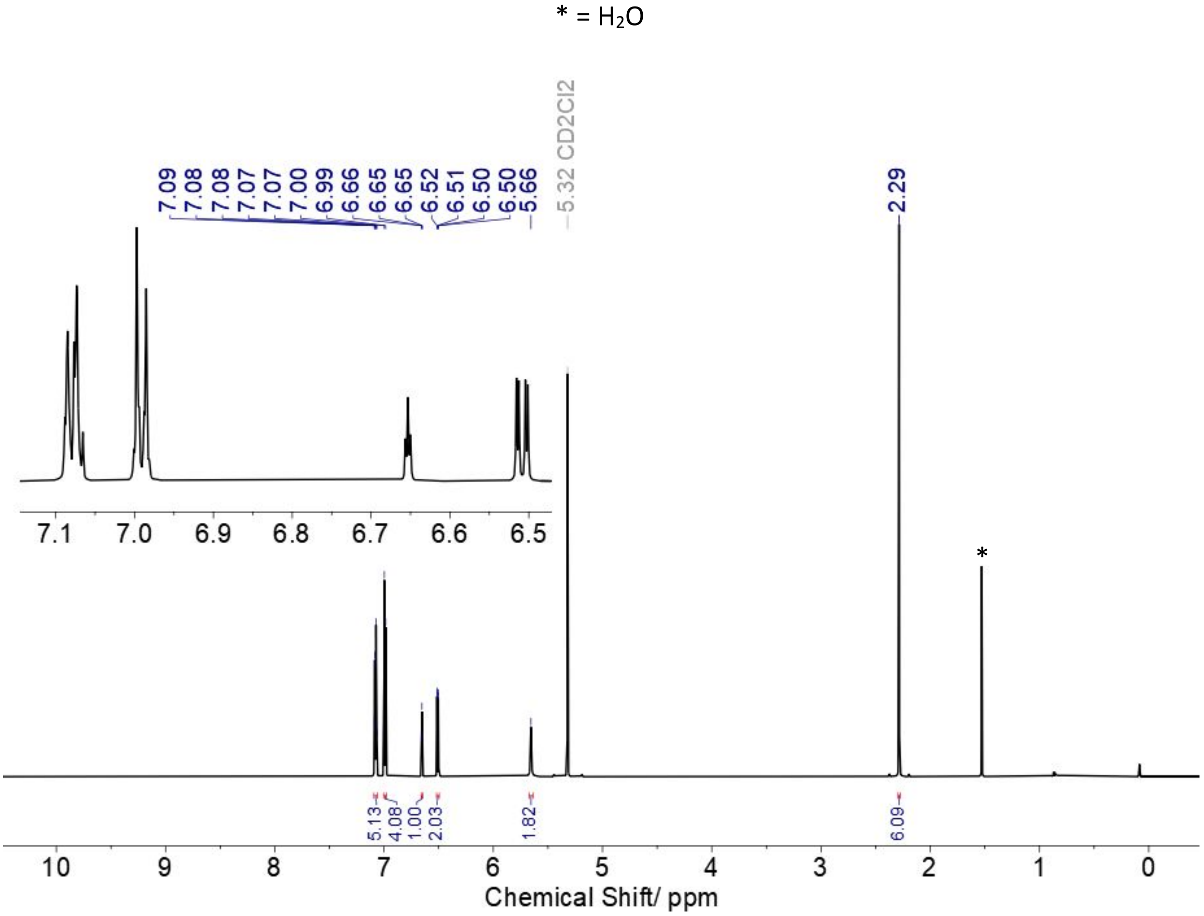

Figure S4. The 700 MHz ^{1}H NMR spectra of ***N*[1],*N*[3]-di-*p*-tolylbenzene-1,3-diamine (Compound S1)** measured at 298 K in CD_2Cl_2.

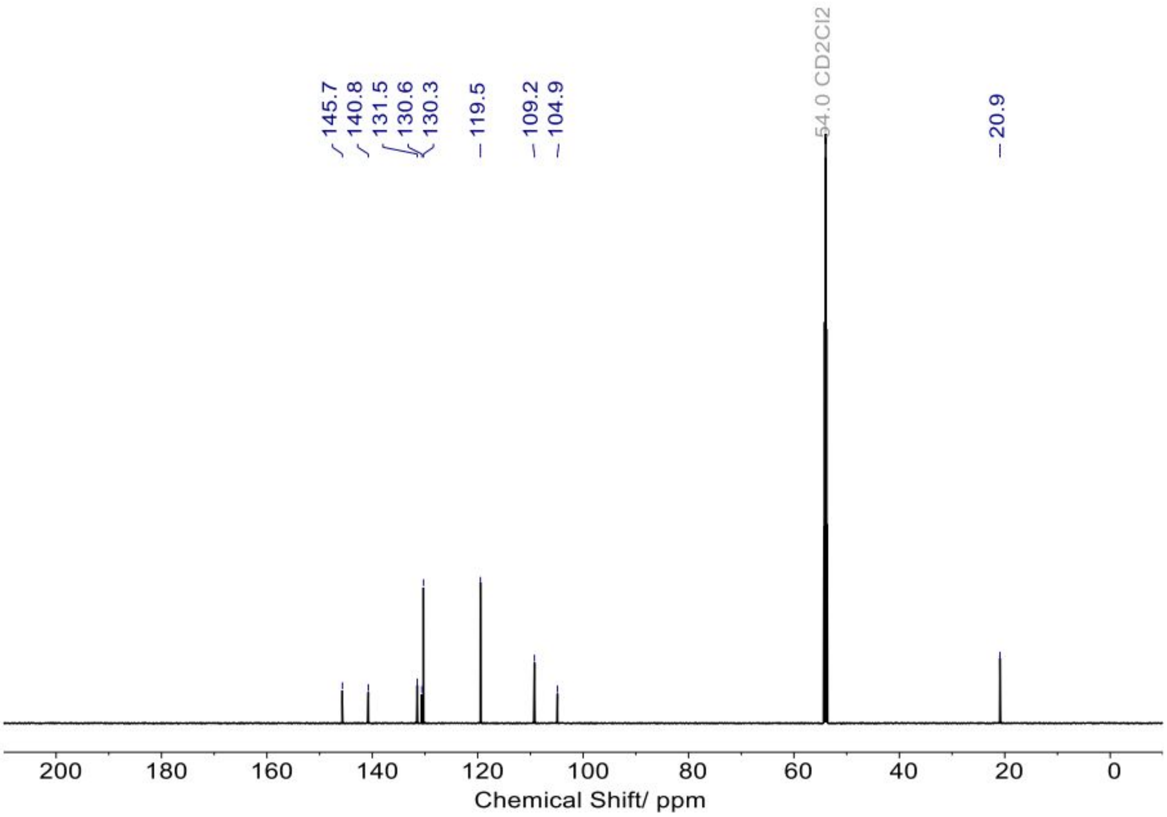

Figure S5. The 176 MHz ^{13}C NMR spectra of ***N*¹,*N*³-di-*p*-tolylbenzene-1,3-diamine (Compound S1)** measured at 298 K in CD_2Cl_2.

1-bromo-3-(3-(*tert*-butyl)phenoxy)-5-chlorobenzene (Compound S2)

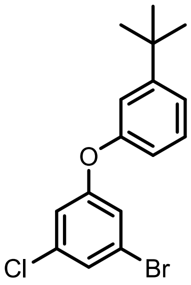

* = H_2O

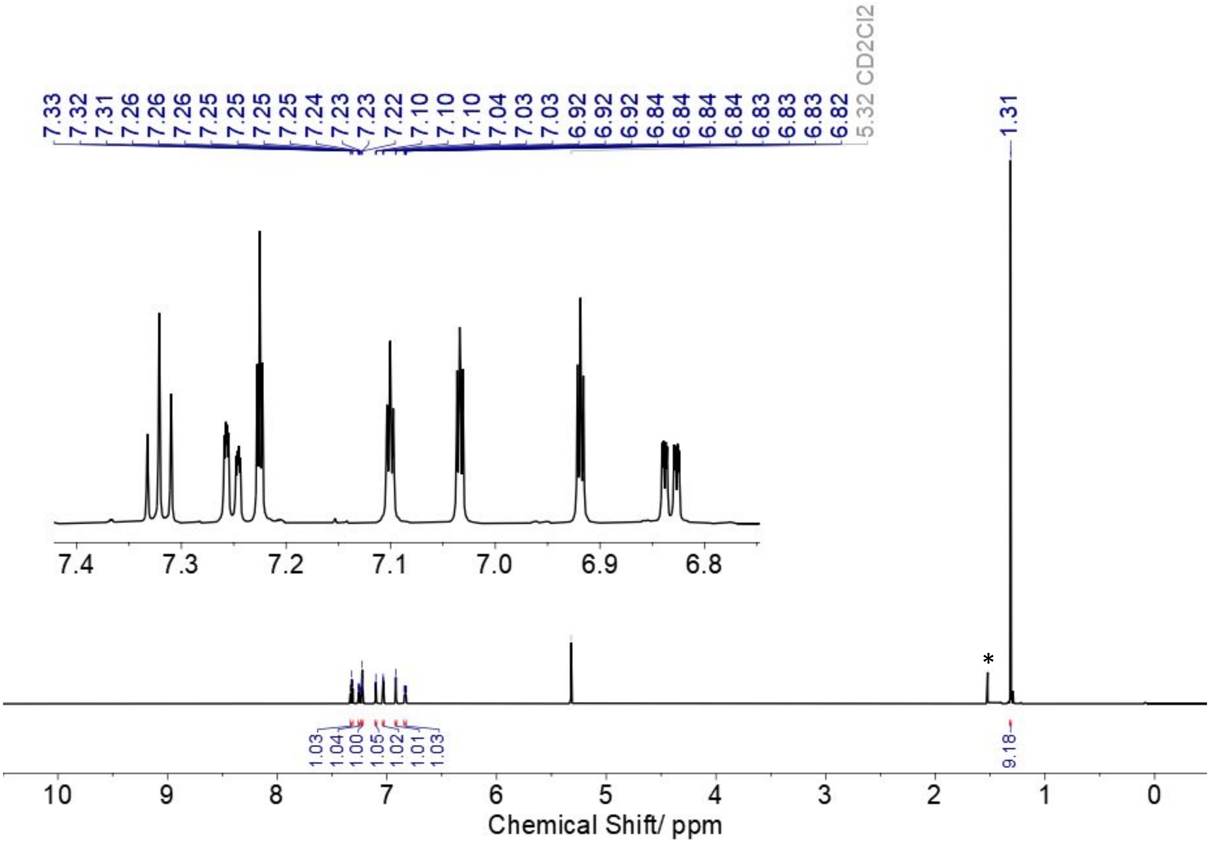

Figure S6. The 700 MHz ^{1}H NMR spectra of **1-bromo-3-(3-(*tert*-butyl)phenoxy)-5-chlorobenzene (Compound S2)** measured at 298 K in CD_2Cl_2.

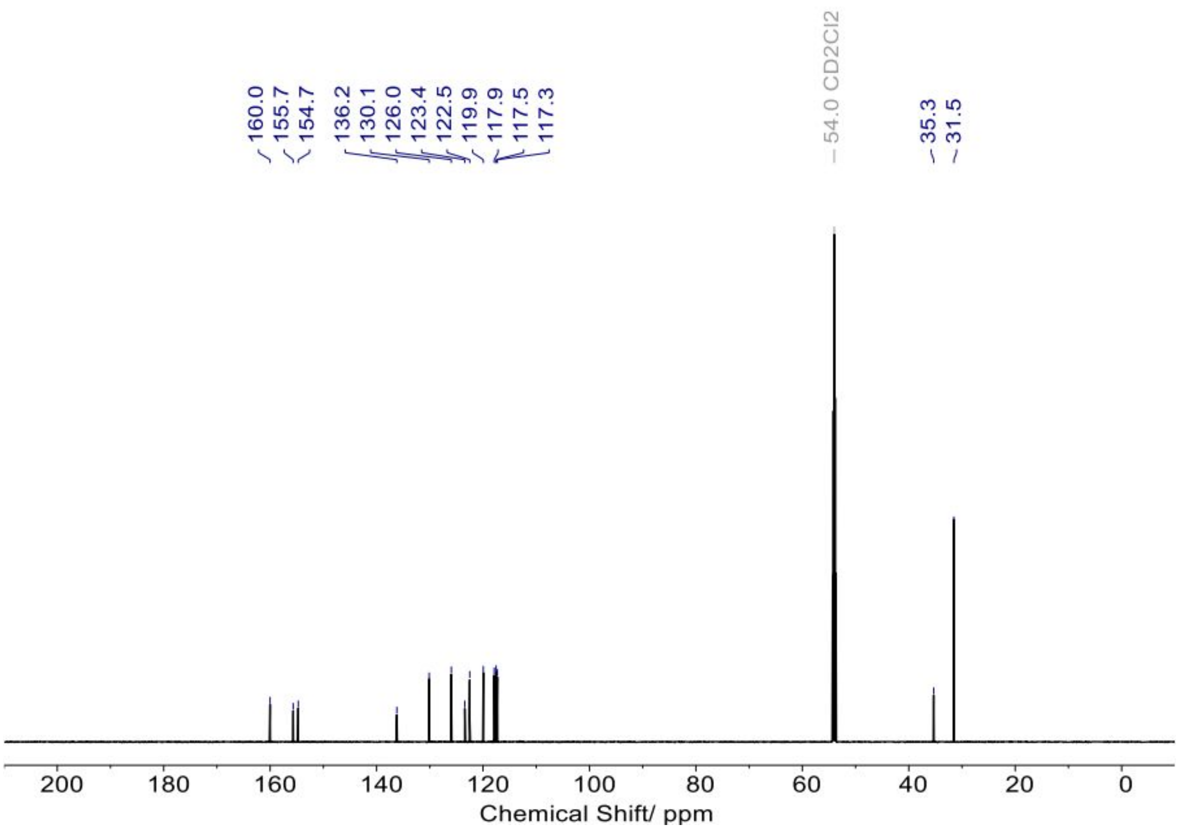

Figure S7. The 176 MHz ^{13}C NMR spectra of **1-bromo-3-(3-(*tert*-butyl)phenoxy)-5-chlorobenzene (Compound S2)** measured at 298 K in CD_2Cl_2.

N^1,N^3-bis(3-(3-(*tert*-butyl)phenoxy)-5-chlorophenyl)-N^1,N^3-di-*p*-tolylbenzene-1,3-diamine (Compound S3)

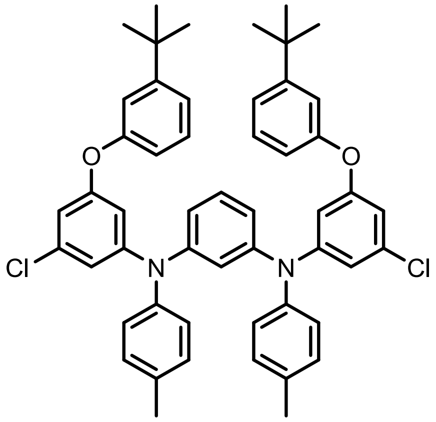

* = H_2O

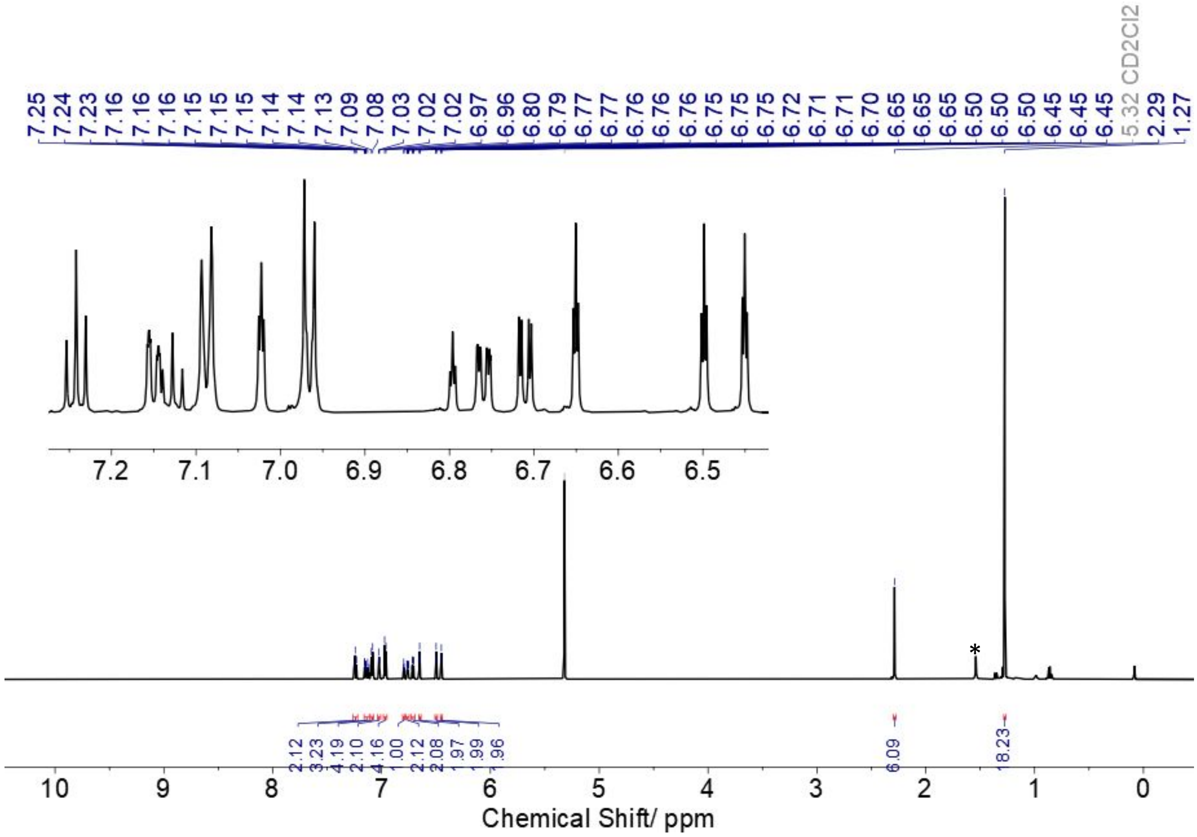

Figure S8. The 700 MHz ^{1}H NMR spectra of ***N*^{1},*N*^{3}-bis(3-(3-(*tert*-butyl)phenoxy)-5-chlorophenyl)-*N*^{1},*N*^{3}-di-*p*-tolylbenzene-1,3-diamine (Compound S3)** measured at 298 K in CD_2Cl_2.

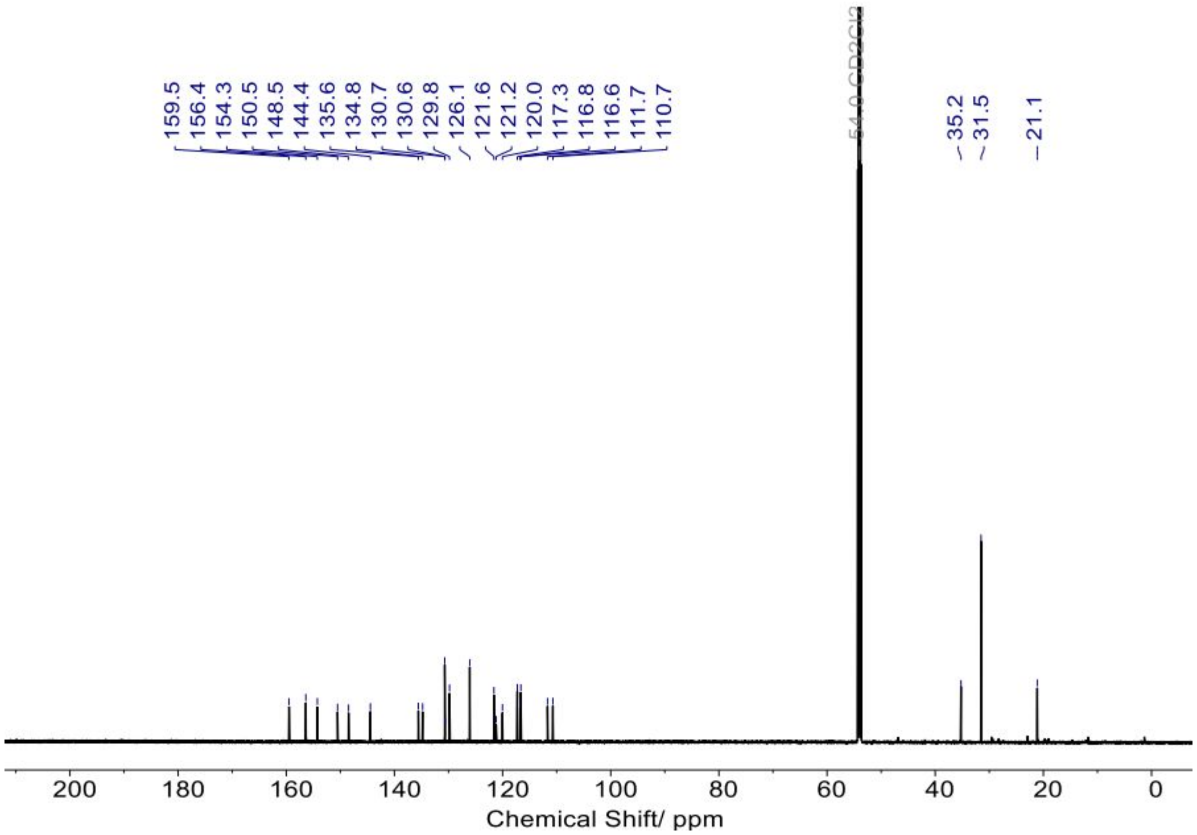

Figure S9. The 175 MHz ^{13}C NMR spectra of ***N*[1],*N*[3]-bis(3-(3-(*tert*-butyl)phenoxy)-5-chlorophenyl)-*N*[1],*N*[3]-di-*p*-tolylbenzene-1,3-diamine (Compound S3)** measured at 298 K in CD_2Cl_2.

Compound 1

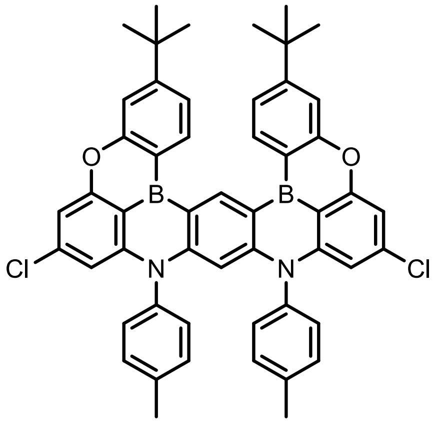

* = H_2O

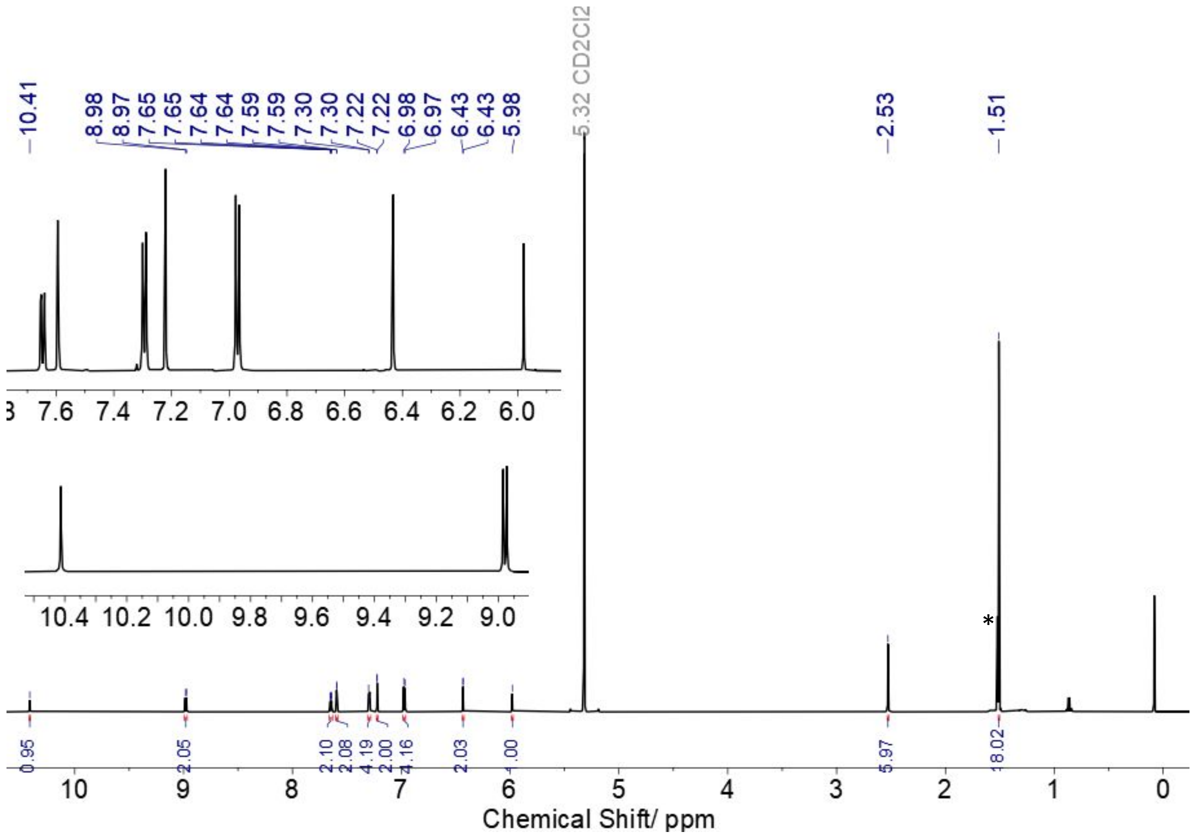

Figure S10. The 700 MHz ^{1}H NMR spectra of **Compound 1** measured at 298 K in CD_2Cl_2.

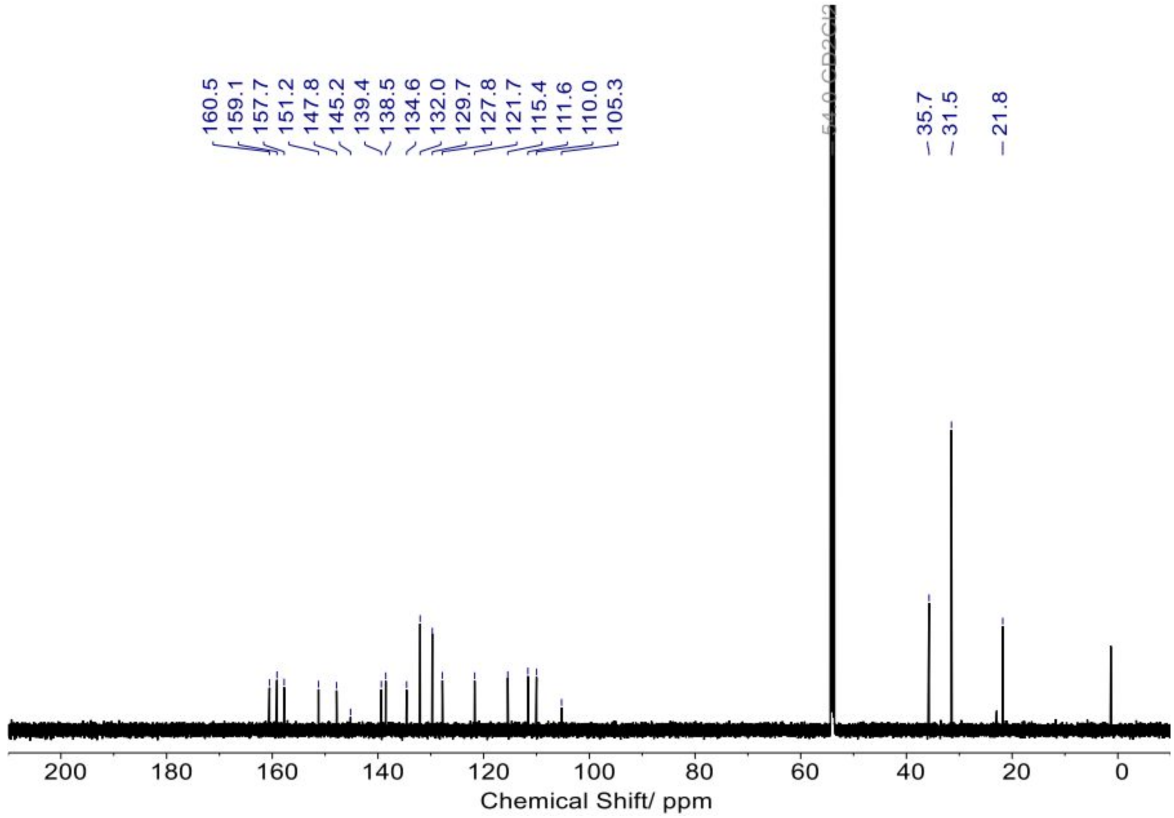

Figure S11. The 176 MHz ^{13}C NMR spectra of **Compound 1** measured at 298 K in CD_2Cl_2.

OMeBOBO (Compound 2)

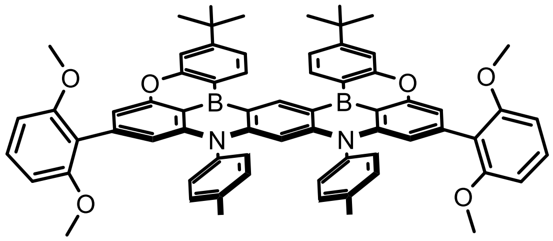

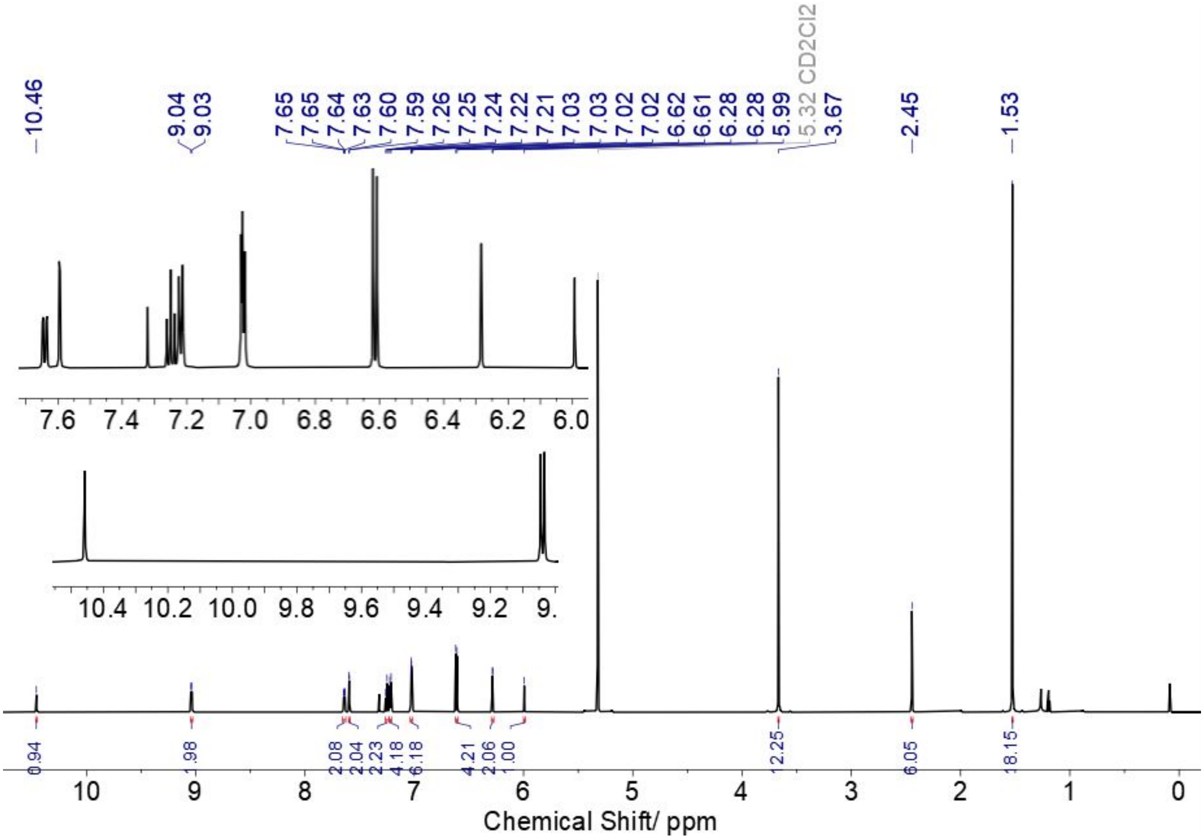

Figure S12. The 700 MHz ^{1}H NMR spectra of **OMeBOBO (Compound 2)** measured at 298 K in CD_2Cl_2.

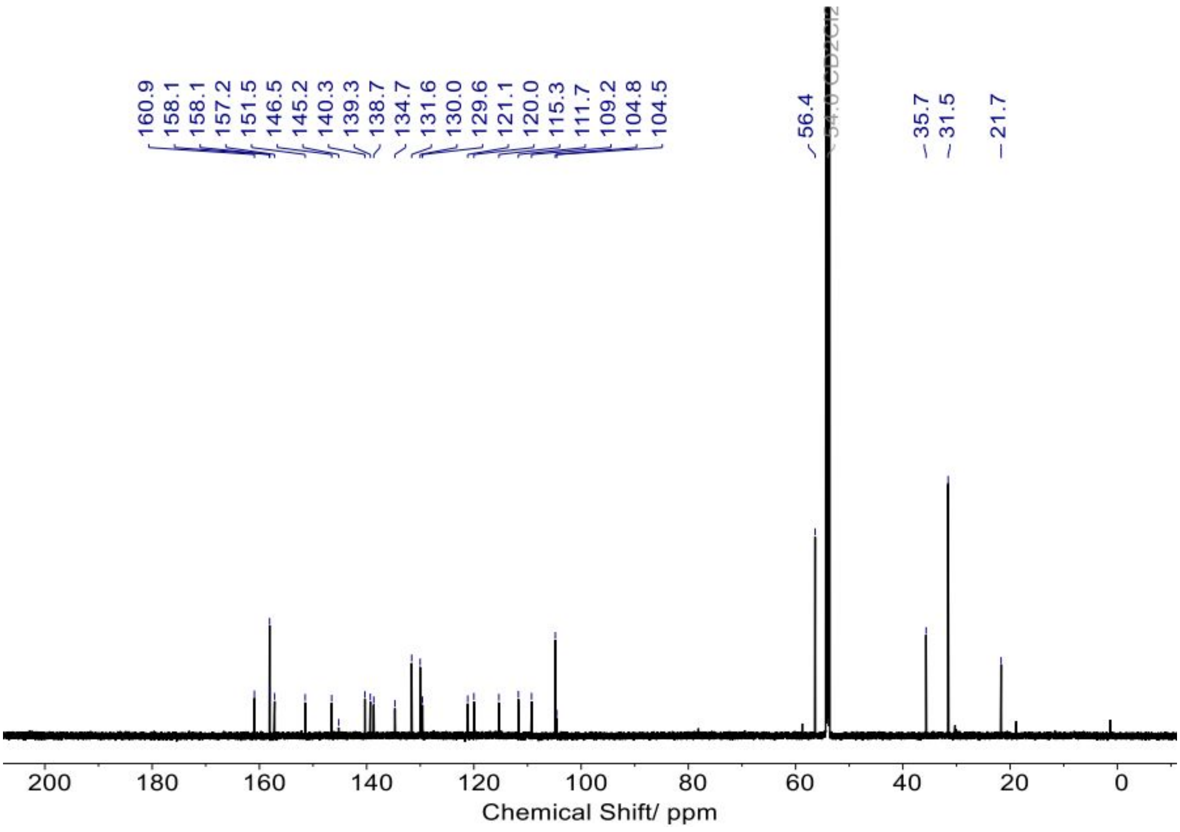

Figure S13. The 176 MHz ^{13}C NMR spectra of **OMeBOBO (Compound 2)** measured at 298 K in CD_2Cl_2.

(2,6-bis(benzyloxy)phenyl)boronic acid

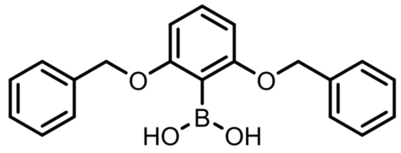

* = H_2O

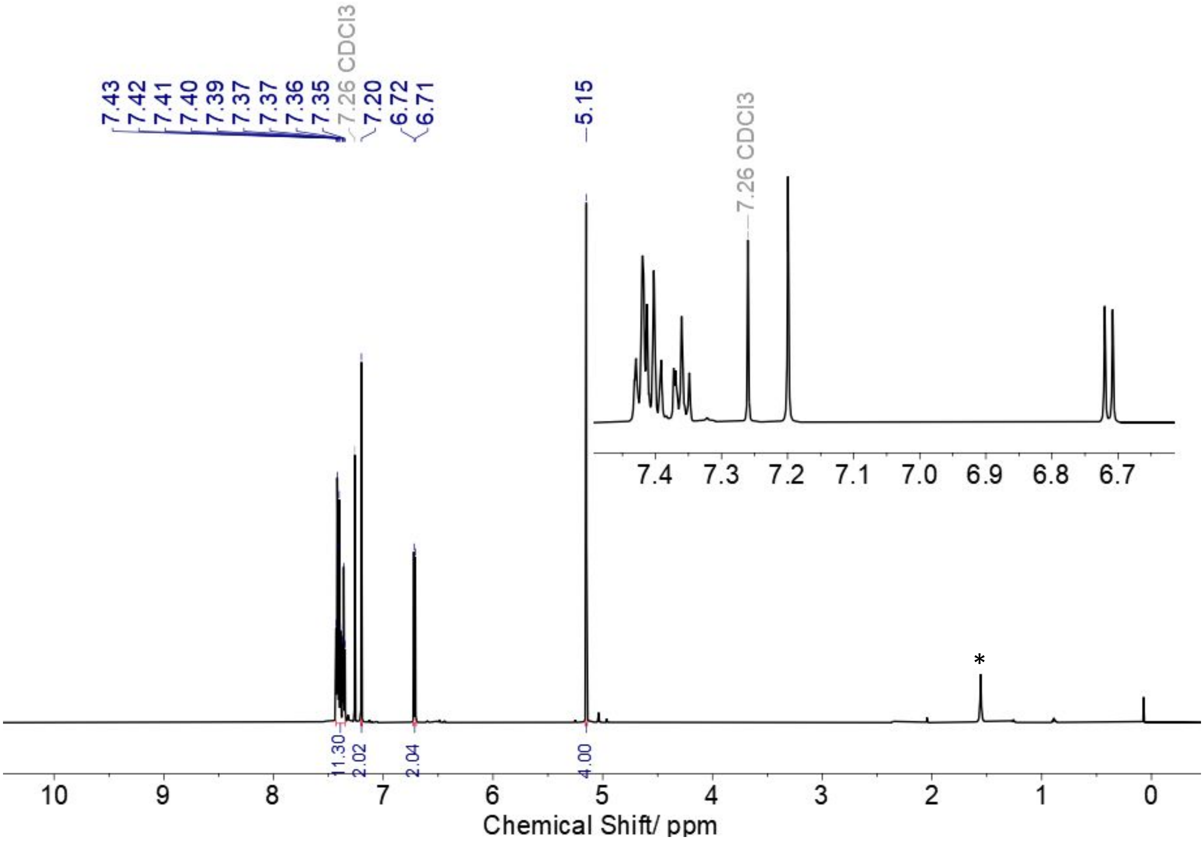

Figure S14. The 700 MHz ^{1}H NMR spectra of **(2,6-bis(benzyloxy)phenyl)boronic acid** measured at 298 K in $CDCl_3$.

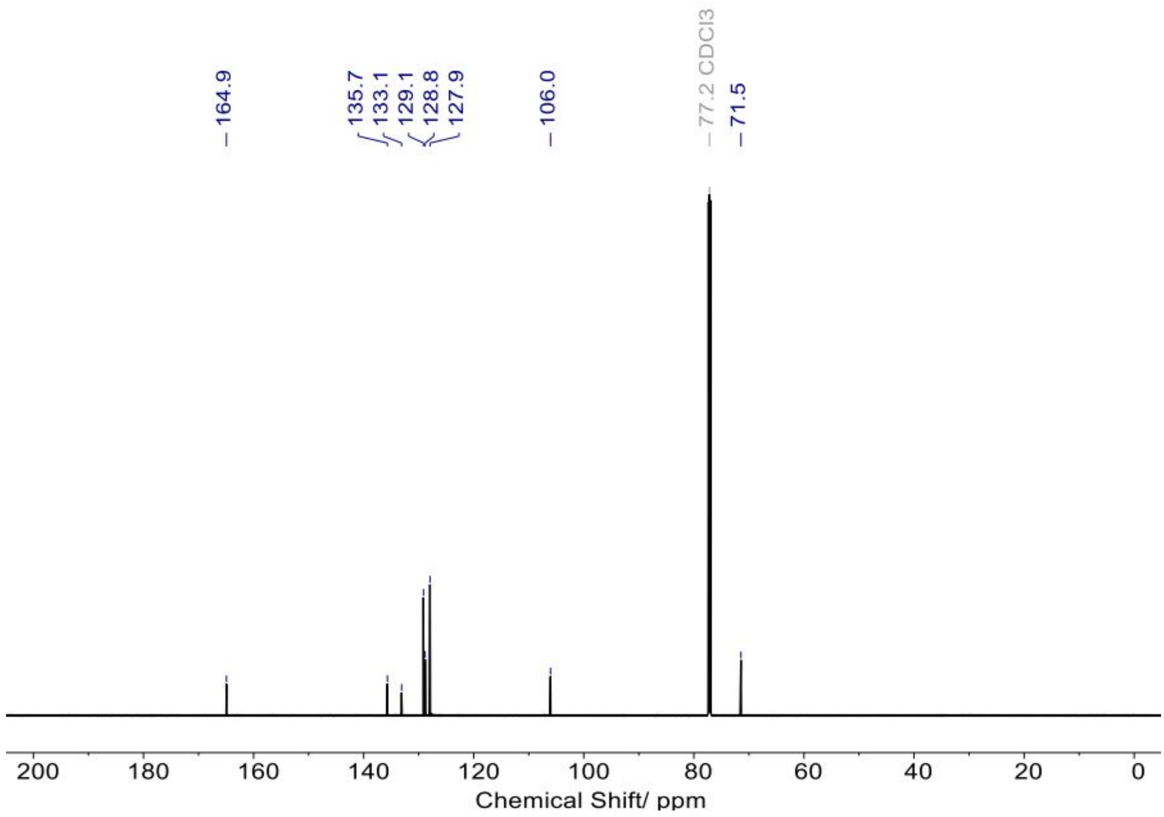

Figure S15. The 176 MHz ^{13}C NMR spectra of **(2,6-bis(benzyloxy)phenyl)boronic acid** measured at 298 K in $CDCl_3$.

Compound 3

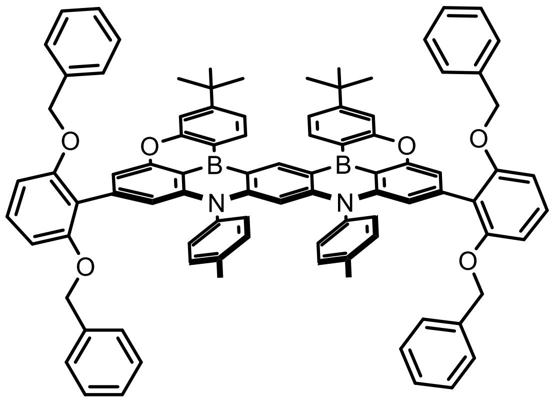

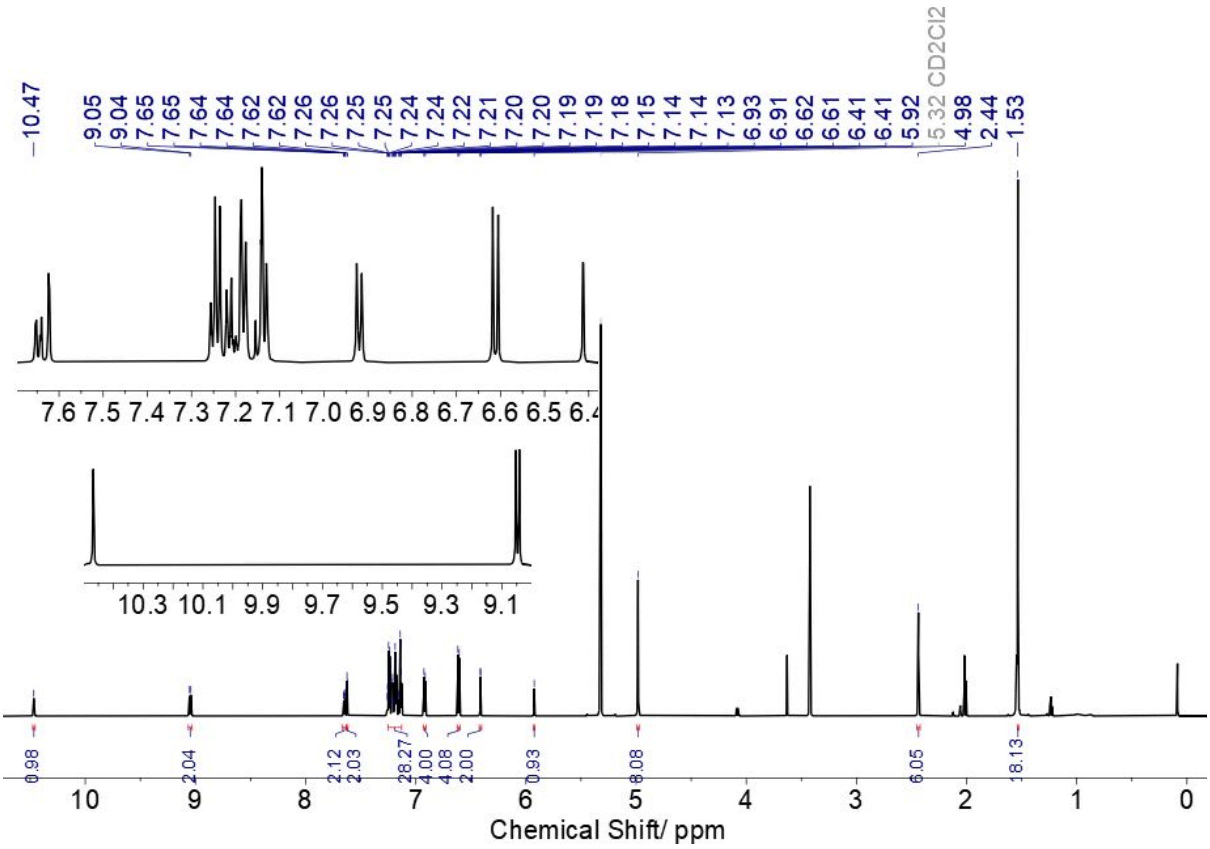

Figure S16. The 700 MHz ^{1}H NMR spectra of **Compound 3** measured at 298 K in CD_2Cl_2.

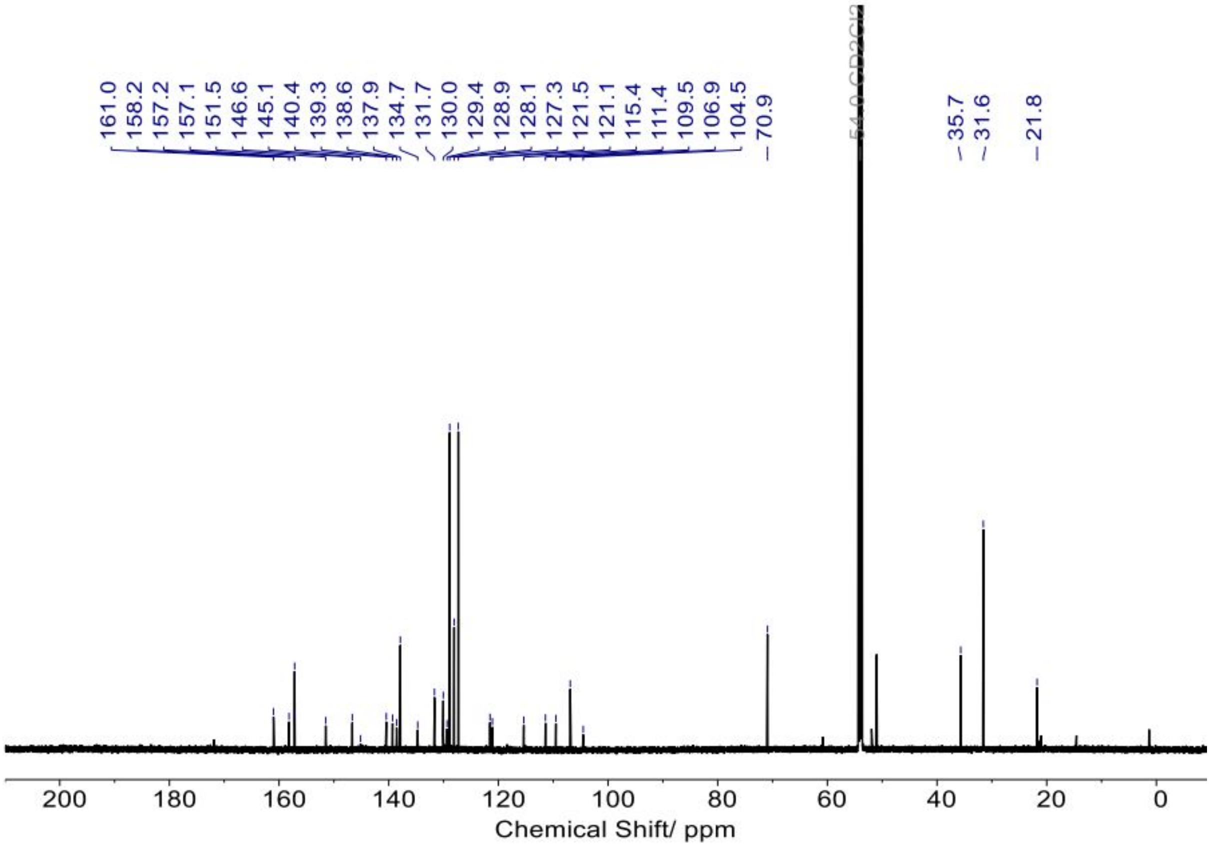

Figure S17. The 176 MHz ^{13}C NMR spectra of **Compound 3** measured at 298 K in CD_2Cl_2.

Compound 4

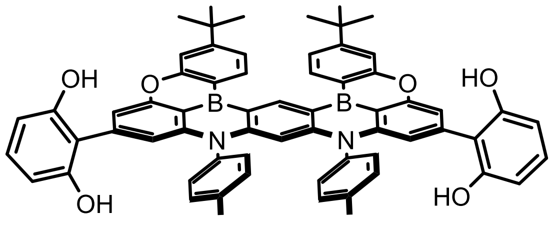

* = H_2O

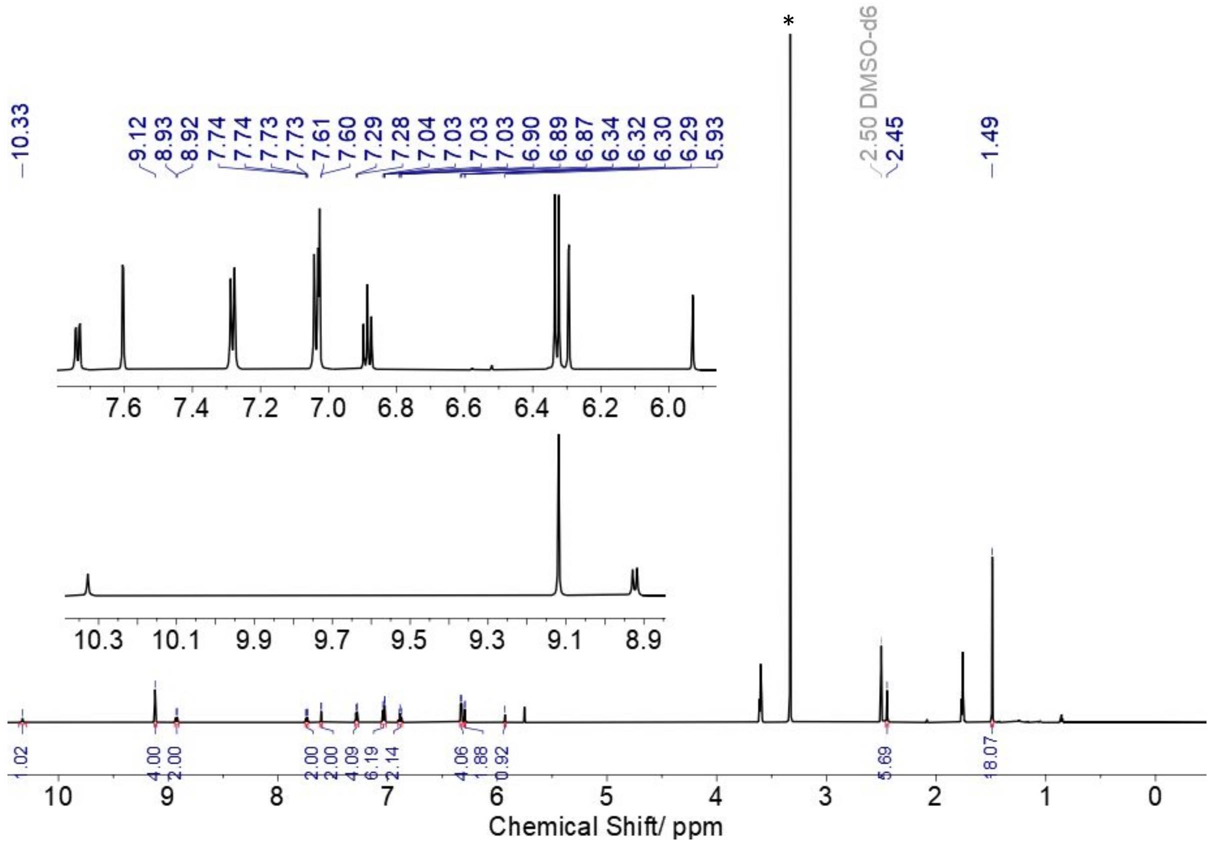

Figure S18. The 700 MHz ^{1}H NMR spectra of **Compound 4** measured at 298 K in DMSO-d_6.

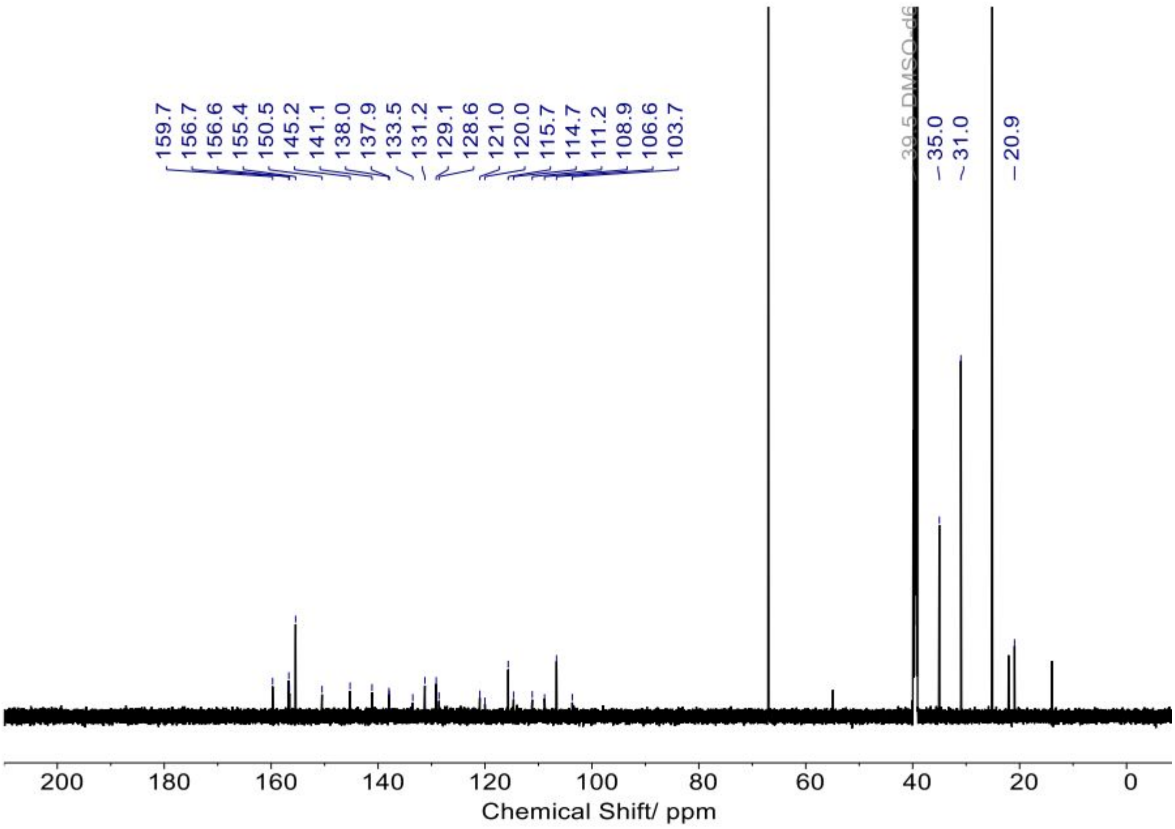

Figure S19. The 176 MHz ^{13}C NMR spectra of **Compound 4** measured at 298 K in DMSO-d_6.

EnBOBO (Compound 5)

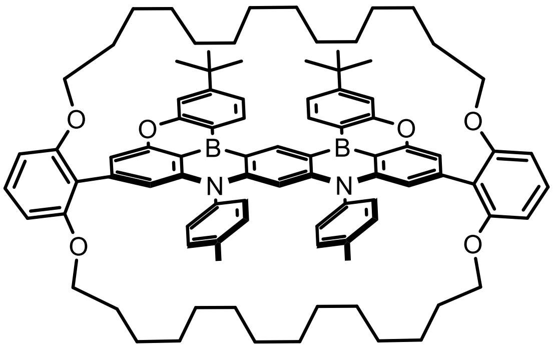

* = H_2O

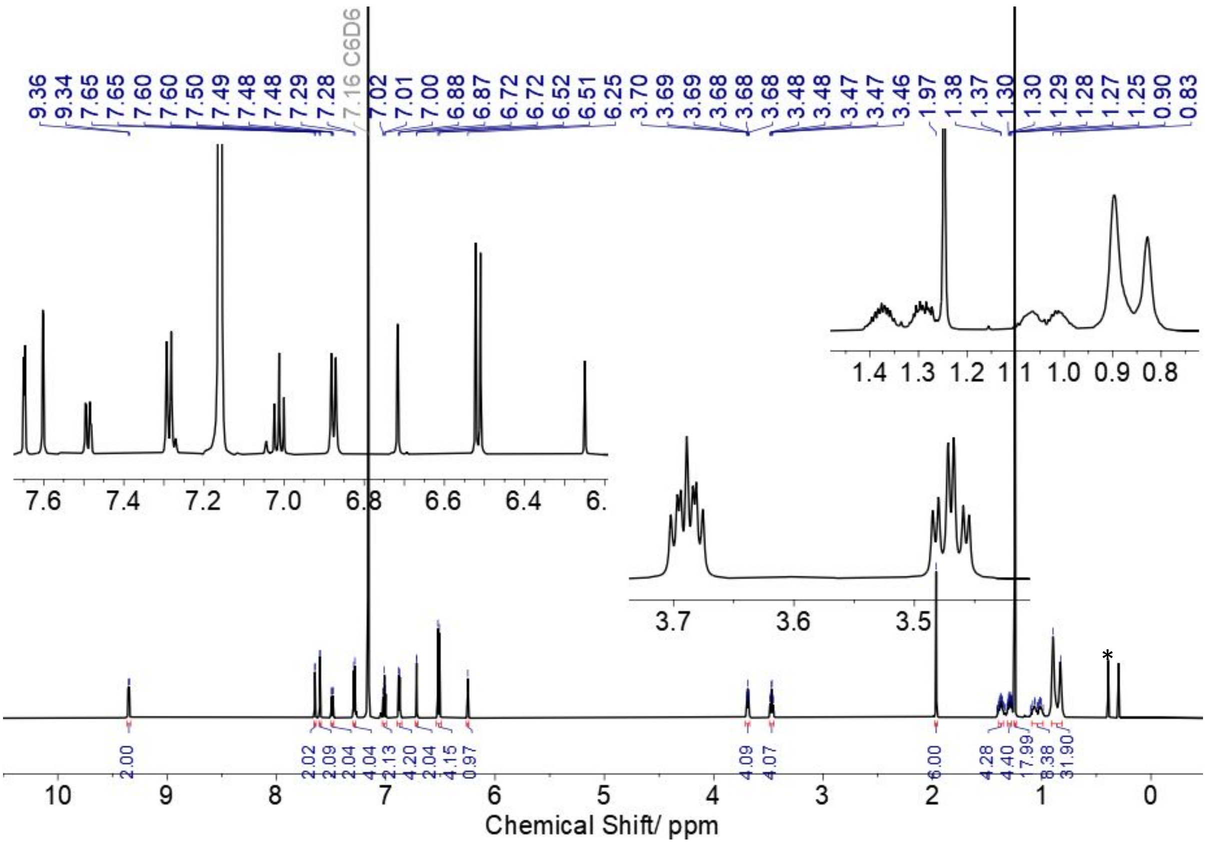

Figure S20. The 700 MHz ^{1}H NMR spectra of **EnBOBO (Compound 5)** measured at 298 K in C_6D_6.

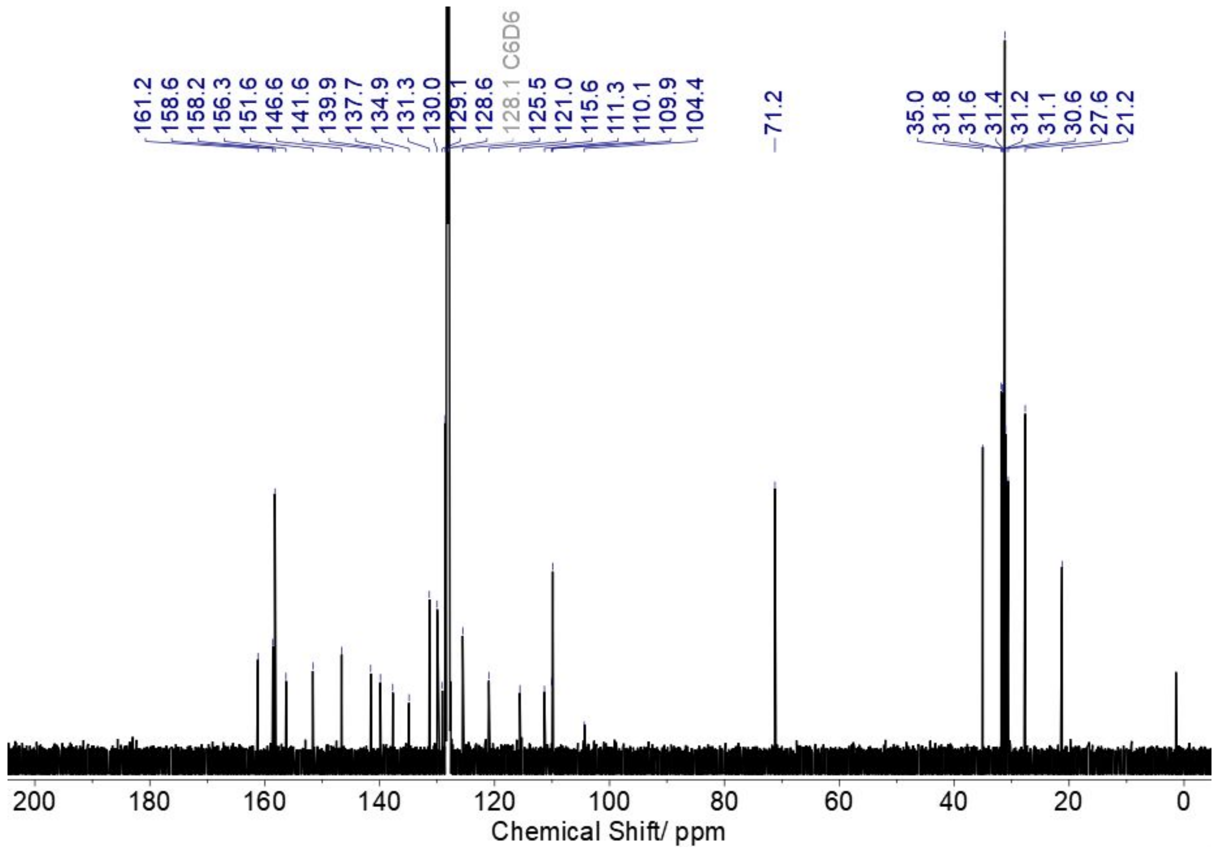

Figure S21. The 176 MHz ^{13}C NMR spectra of **EnBOBO (Compound 5)** measured at 298 K in C_6D_6.

TDBA-PAS

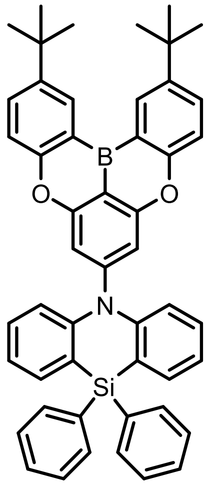

* = H_2O

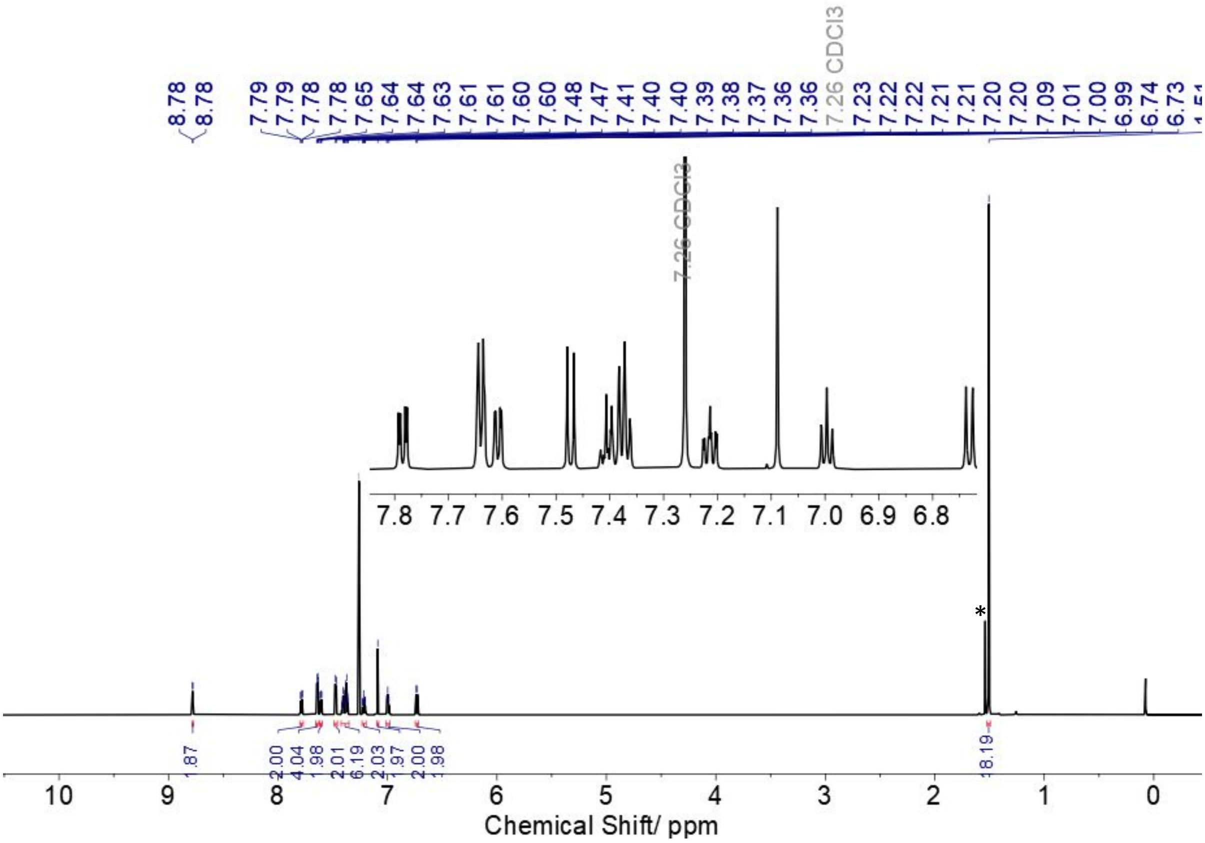

Figure S22. The 700 MHz ^{1}H NMR spectra of **TDBA-PAS** measured at 298 K in $CDCl_3$.

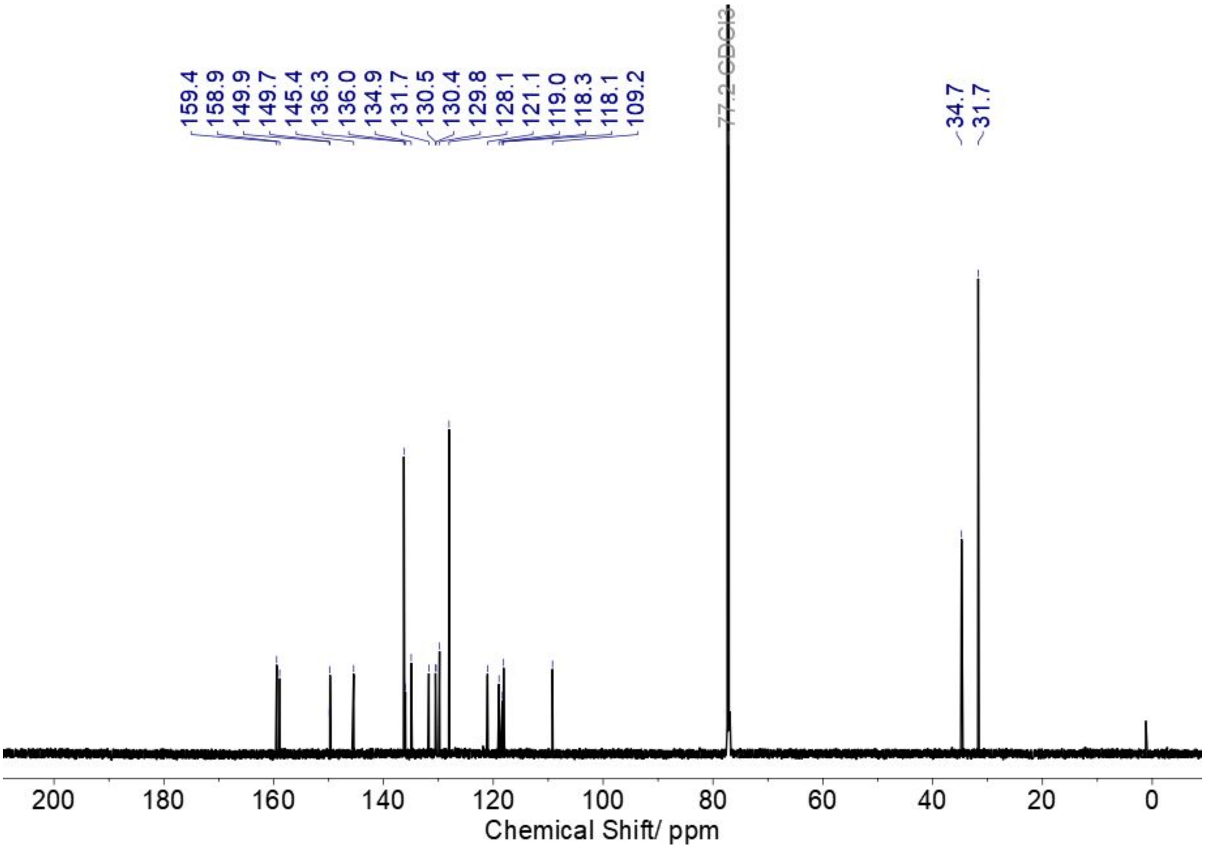

Figure S23. The 176 MHz ^{13}C NMR spectra of **TDBA-PAS** measured at 298 K in $CDCl_3$.

TDBA-SPQ

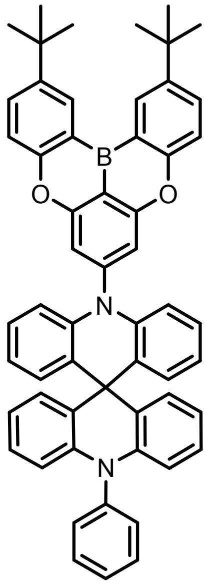

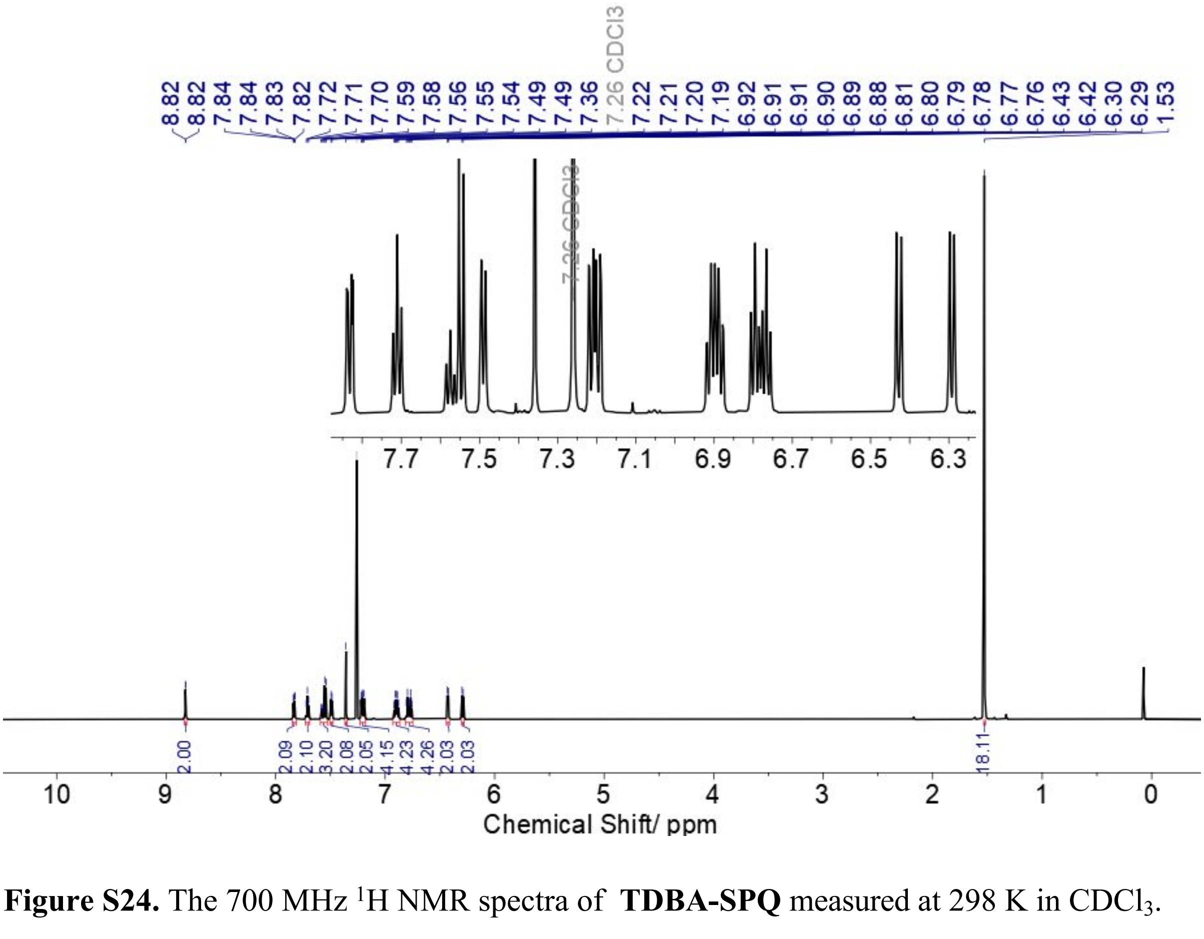

Figure S24. The 700 MHz ^{1}H NMR spectra of **TDBA-SPQ** measured at 298 K in $CDCl_3$.

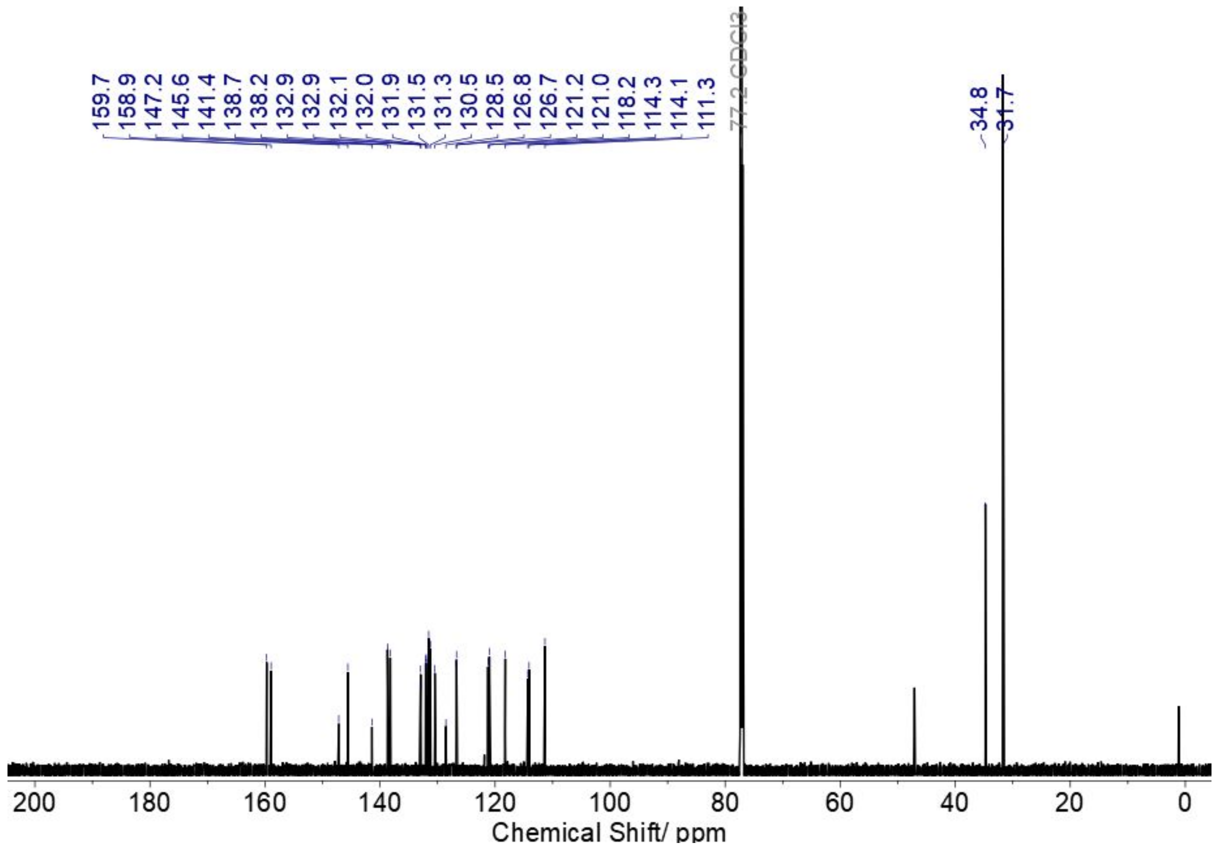

Figure S25. The 176 MHz ^{13}C NMR spectra of **TDBA-SPQ** measured at 298 K in $CDCl_3$.

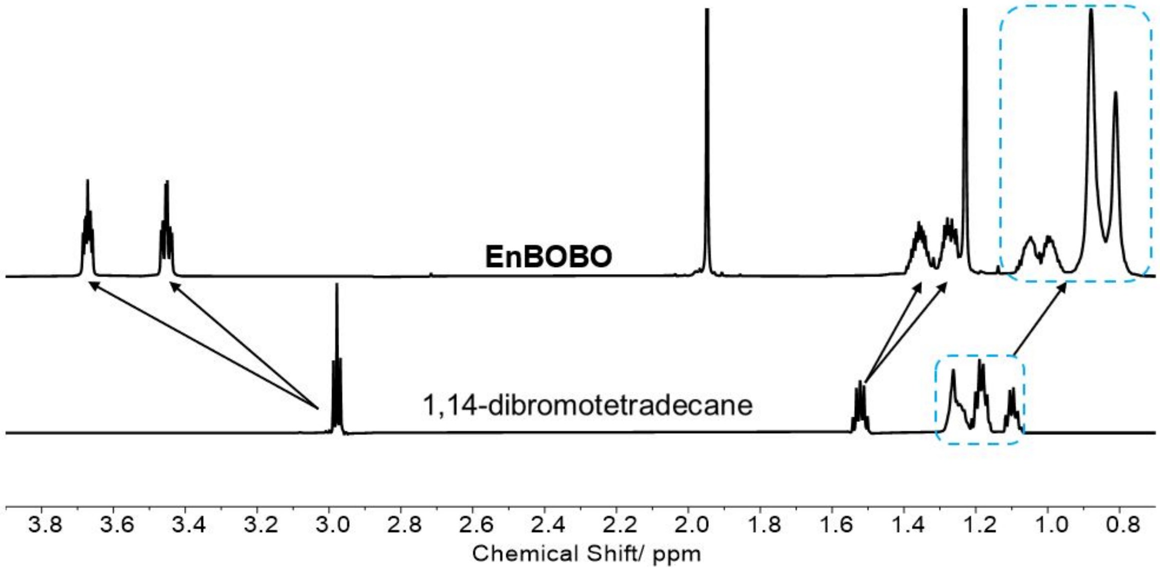

Figure S26. The alkyl region (*ca.* 0.7– 3.8 ppm) of the 700 MHz ^{1}H NMR spectra of **EnBOBO (Compound 5)** and 1,14-dibromotetradecane measured at 298 K in C_6D_6. Key resonance shifts upon encapsulation are indicated with arrows.

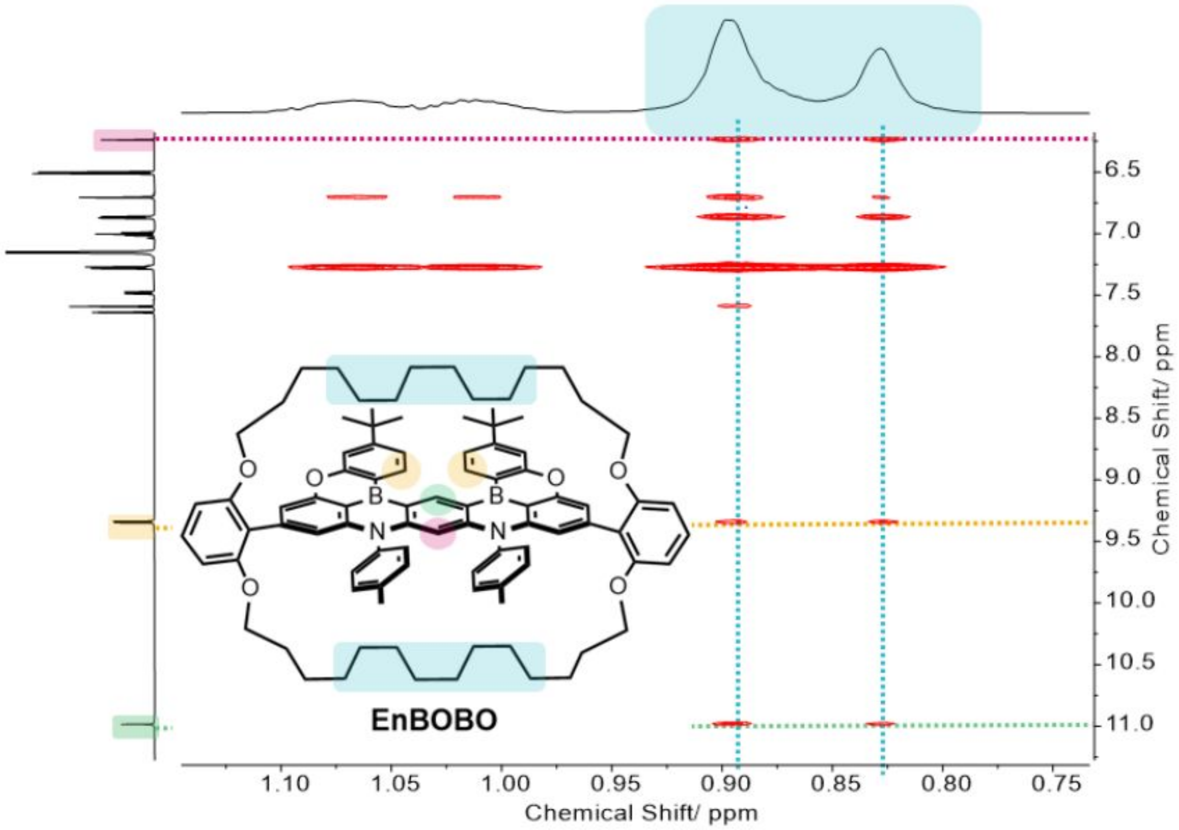

Figure S27. The 700 MHz ^{1}H-^{1}H ROESY spectra of **EnBOBO (Compound 5)** measured at 298 K in C_6D_6. ROEs (red) are observed between the central alkyl chain environments (light blue) and the central aromatic environments (yellow, green, pink).

S4. X-Ray Crystallography

Single crystals were grown by the diffusion of *n*-hexane into a dichloromethane solution of **EnBOBO**. Single-crystal X-ray data for **EnBOBO** were collected on a Bruker D8-QUEST diffractometer, equipped with an Incoatec IμS Cu microsource (λ = 1.5418 Å) and a PHOTON-III detector operating in shutterless mode. The crystal was mounted on a MiTeGen crystal mount using inert polyfluoroether oil and the analysis was carried out under an Oxford Cryosystems open-flow N_2 Cryostream. The control and processing software was Bruker APEX5. Diffraction images were integrated using SAINT in APEX5, and a multi-scan correction was applied using SADABS. The final unit-cell parameters were refined against all reflections. The structure was solved using SHELXT and refined using SHELXL.[7,8]

One alkyl chain (C79-C92) is well ordered and refined without difficulty. The other (C59-C72) shows larger displacement parameters, particularly around the C67–C72 end; this region was split into two components. Restraints were applied to all 1,2 and 1,3 distances of the alkyl chains (one refined distance parameter).

The relatively high R-factors are mainly due to difficulties modelling the dichloromethane solvent molecules. One molecular site was refined as disordered, while another was refined with a single component. Both sites show relatively large ADPs. Omitting the dichloromethane molecules and applying SQUEEZE improves the R-factors to R1 = 0.071, wR2 = 0.236.[9] However: it was preferred to retain the dichloromethane molecules in the final refined model for chemical clarity.

Table S1. A summary of the crystallographic and refinement data for **EnBOBO.**

**CCDC number**	2475574
**Cambridge data number**	HB_B1_0114
**Chemical formula**	$C_{92}H_{106}B_2N_2O_6$, $2CH_2Cl_2$
**Formula weight**	1527.25
**Temperature/ K**	180(2)
**Crystal system**	monoclinic
**Space group**	$P2_1/n$
**a/ Å**	18.5583(6)
**b/ Å**	13.1945(4)
**c/ Å**	34.1308(10)
**α/ °**	90
**β/ °**	97.605(2)
**γ/ °**	90
**Unit-cell volume/ Å^3**	8284.0(4)
**Z**	4
**Calc. density/ g cm^{-3}**	1.225
**F(000)**	3256
**Radiation type**	Cu Kα
**Absorption coefficient/ mm^{-1}**	1.726
**Crystal size/ mm^3**	0.28 x 0.14 x 0.08
**2-Theta range/ degrees**	5.16-137.27
**Completeness to max 2-theta**	0.998
**No. of reflections measured**	157606
**No. of independent reflections**	15247
**R(int)**	0.0907
**No. parameters / restraints**	1022 / 701
**Final R1 values (I > 2σ(I))**	0.0881
**Final wR(F^2) values (all data)**	0.2855
**Goodness-of-fit on F^2**	1.040
**Largest difference peak & hole/ e Å^{-3}**	0.931, -0.829

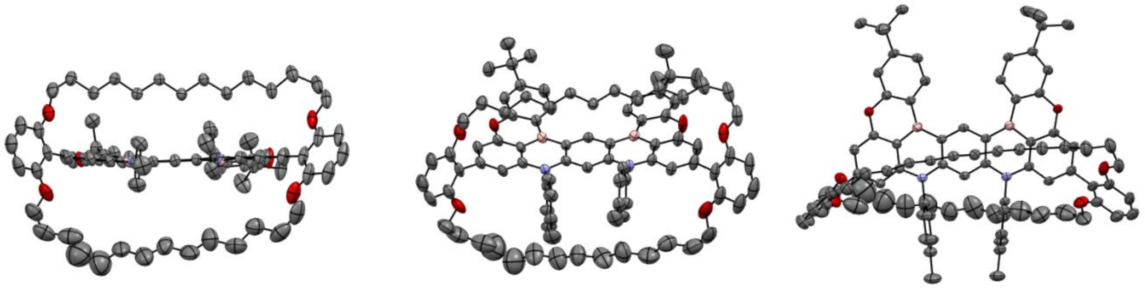

Figure S28. View of the molecular unit of **EnBOBO** from three different perspectives. Displacement ellipsoids are shown at 50% probability. H atoms and minor disorder of one alkyl chain are omitted for clarity.

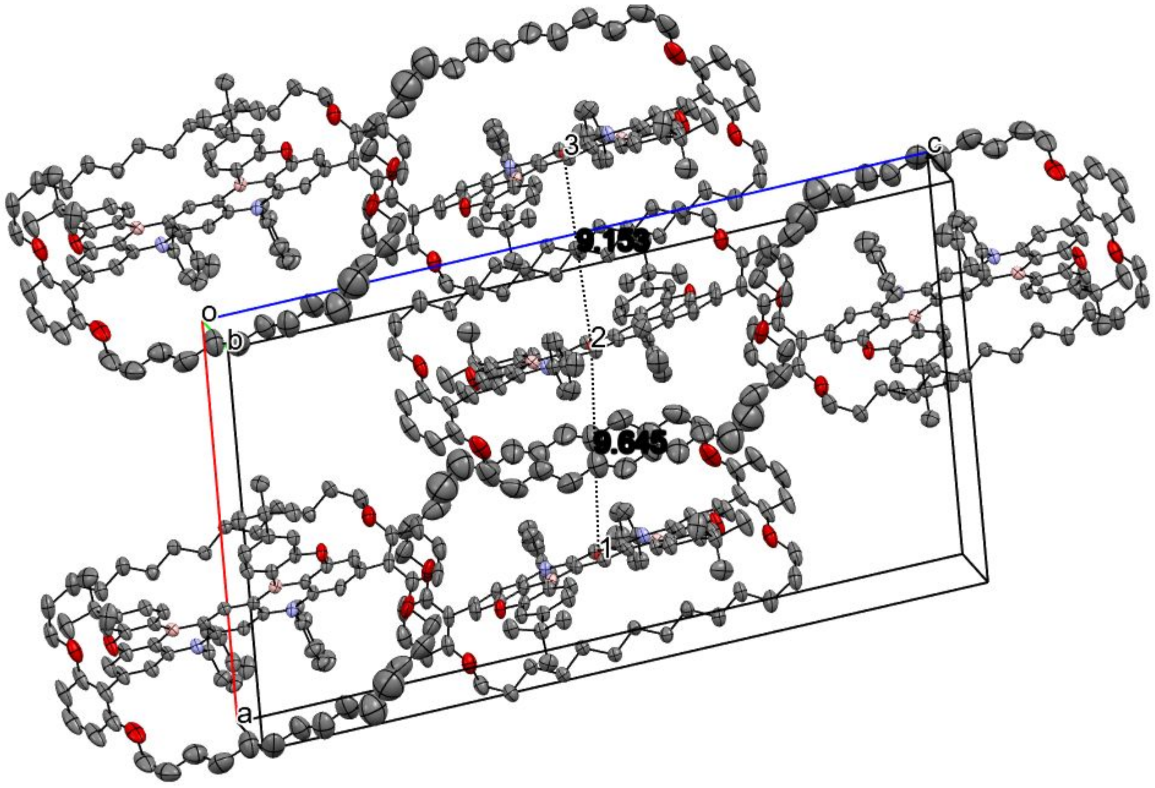

Figure S29. View of the crystal packing and unit cell of **EnBOBO**. Displacement ellipsoids are shown at 50% probability. H atoms and minor disorder of one alkyl chain are omitted for clarity. The shortest co-facial distances between molecular centroids (1,2 and 2,3) along the **a** direction are approximately 9.645 Å (1,2) and 9.153 Å (2,3).

S5. Thermogravimetric Analysis

Thermogravimetric analysis (TGA) was performed on **EnBOBO** and **OMeBOBO** under a nitrogen atmosphere. The data was collected using a TGA5500 system (TA Instruments). The TGA temperature was calibrated against standards of alumel (Curie point = 153.0 °C), nickel (Curie point = 358.2 °C), nickel-83:cobalt-17 (Curie point = 554.4 °C), and nickel-63:cobalt-37 (Curie point = 746.4 °C). The TGA mass was calibrated against standards of 100.0000 mg and 1000.0000 mg. Measurements were performed with a heating rate of 10 °C min^{-1} and with a nitrogen flow rate of 30 mL min^{-1}.

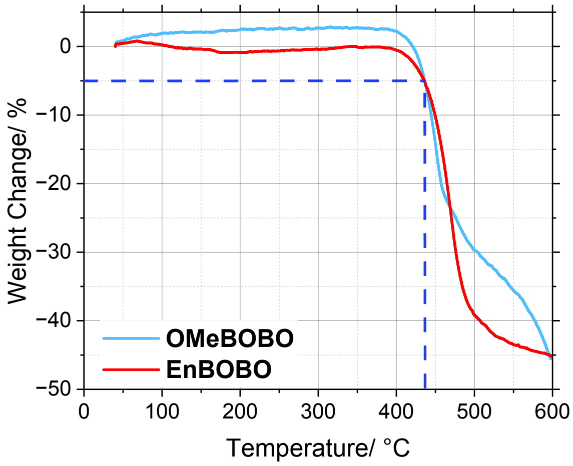

Figure S30. Thermogravimetric analysis traces of **EnBOBO** and **OMeBOBO**. 5% Weight loss occurs at 436 °C in both cases.

S6. Cyclic Voltammetry

Cyclic voltammetry (CV) was performed using a PalmSens EmStat4S. Each cell consisted of a glassy carbon working electrode, a platinum wire counter electrode, and a Ag/AgCl wire as a quasi-reference electrode. Each solution was prepared using an anhydrous solvent (**TDBA-PAS** and **TDBA-SPQ**: DCM, **EnBOBO** and **OMeBOBO**: THF) with 0.1 M tetrabutylammonium hexafluorophosphate (n-Bu_4NPF_6) as the supporting electrolyte and was degassed by argon bubbling prior to each measurement. The voltammograms were recorded under an argon atmosphere with a scan rate of 100 mV s^{-1}. Ferrocene was used as an internal reference where the half-potential ($E_{1/2}$) of the FcH/ FcH^+ redox couple (~0 V) was used to calibrate the measured potential values.

Table S2. Electrochemical properties of **OMeBOBO**, **EnBOBO**, **TDBA-PAS**, and **TDBA-SPQ.**

	$E_{ox}{}^{a}$/ V	HOMO[b]/ eV	$E_g{}^{c}$/ eV	LUMO[d]/ eV
**OMeBOBO**	0.70	-5.50	2.79	-2.71
**EnBOBO**	0.72	-5.52	2.82	-2.70
**TDBA-PAS**	0.71	-5.51	3.16	-2.35
**TDBA-SPQ**	0.52	-5.32	3.02	-2.30

[a]The oxidation potential (E_{ox}) was determined from the onset of the first oxidation process (irreversible). [b]HOMO energies were determined by subtracting the oxidation potential (E_{ox}) from -4.80 eV, which is the energy level of the FcH/FcH^+ redox couple relative to the vacuum level. [c]The band gap (E_g) is estimated from the intersection point of the UV/Visible absorption and photoluminescence spectra of dilute toluene solutions (see **S7**). [d]LUMO energies were determined by adding the band gap (E_g) to the HOMO energies.

S7. Photophysical Characterisation

Steady-state Absorption

The UV/Visible molar extinction (UV/Vis) spectra of **OMeBOBO** and **EnBOBO** dissolved in toluene were measured using a Shimadzu UV-1800 spectrophotometer and a 10 mm pathlength cuvette. The solutions were prepared to give absorbance values in the range where the linear Beer-Lambert law applies (*ca.* 0.1 – 1.0), which corresponded to sample concentrations of approximately 10 μM. A sample preparation method which minimised the potential sources of error in the concentration of each sample was employed. The UV/Visible absorption spectra of dilute toluene solutions of **TDBA-PAS** and **TDBA-SPQ** (the lowest energy electronic transition maximum had an absorbance of approximately 0.1) were also measured.

Table S3. Absorption properties of **OMeBOBO** and **EnBOBO** in toluene.

Emitter	Absorbance @ λ_{max}	FWHM/ nm (eV)	Concentration/ μM	Extinction Coefficient ε @ λ_{max}/ $dm^3\ mol^{-1}\ cm^{-1}$
**OMeBOBO**	0.405	22 (0.14)	9.61	42144
**EnBOBO**	0.372	19 (0.12)	8.73	42580

Steady-state Photoluminescence

The Photoluminescence (PL) spectra of dilute toluene solutions (*ca.* 2.5 μM) of **OMeBOBO** and **EnBOBO** were measured using an Edinburgh Instruments FS5 spectrofluorometer and a 10 mm pathlength cuvette. Similarly, dilute *n*-hexane and dichloromethane (DCM) solutions of **OMeBOBO** and **EnBOBO** (the lowest energy electronic transition maximum had an absorbance of approximately 0.1) were also measured.

Table S4. Photoluminescence properties of **OMeBOBO** and **EnBOBO** in *n*-hexane and DCM.

Emitter	λ_{PL}/ nm	FWHM/ nm (eV)	CIE_{xy}
		***n*-hexane**	
**OMeBOBO**	444	16 (0.10)	(0.154, 0.032)
**EnBOBO**	440	16 (0.10)	(0.156, 0.025)
		**DCM**	
**OMeBOBO**	461	36 (0.19)	(0.134, 0.089)
**EnBOBO**	452	32 (0.18)	(0.142, 0.059)

20 nm-thick device films were thermally evaporated in a vacuum chamber (Angstrom Engineering) under high vacuum (~10^{-7} torr) on glass substrates. Then, the films were encapsulated in a nitrogen glove box to prevent degradation by water and oxygen. The doping concentrations applied in the films are weight percentages and are detailed in **Figure S32** and **Table S5** below.

Toluene stock solutions of ZEONEX® and the emitters (**EnBOBO** and **OMeBOBO**) were prepared at concentrations of 10 mg mL^{-1} and 1 mg mL^{-1}, respectively. These stock solutions were combined in an appropriate volume ratio to provide the solutions with an emitter doping concentration of 1 wt%. A vacuum-free spin-coater from Ossila was equipped with a custom 3D-printed circular substrate holder. 30 µL of each solution mixture was spin-coated onto quartz substrates, which were cleaned by sonication in acetone followed by isopropanol for 15 minutes each, at 1600 rpm for 90 seconds in air. Subsequently, these films were transferred to the antechamber of a nitrogen glovebox (0.1 ppm H_2O, <1.0 ppm O_2) and subject to three vacuum-backfill cycles to eliminate any oxygen trapped in the film. The substrates were then

encapsulated under the nitrogen atmosphere of the glovebox using glass substrates and heat-cured polyisobutylene.

The PL spectra of the evaporated and spin-coated films were measured using an Edinburgh Instruments FLS1000 photoluminescence spectrometer. The evaporated films were excited using a wavelength of 365 nm to selectively excite the sensitiser component of the films.

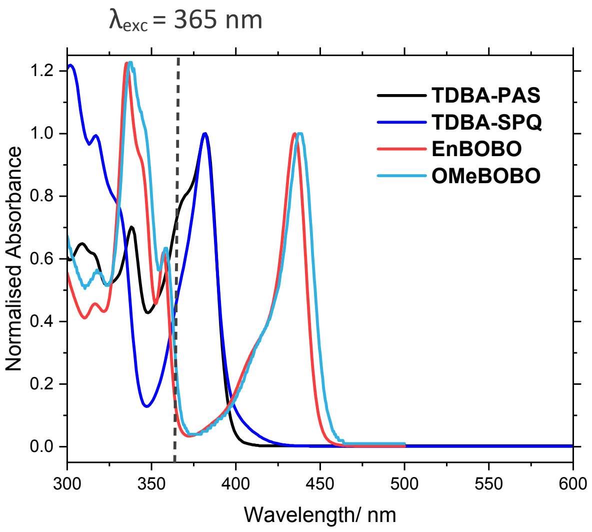

Figure S31. Normalised absorption spectra of dilute toluene solutions of **TDBA-PAS**, **TDBA-SPQ**, **EnBOBO** and **OMeBOBO.**

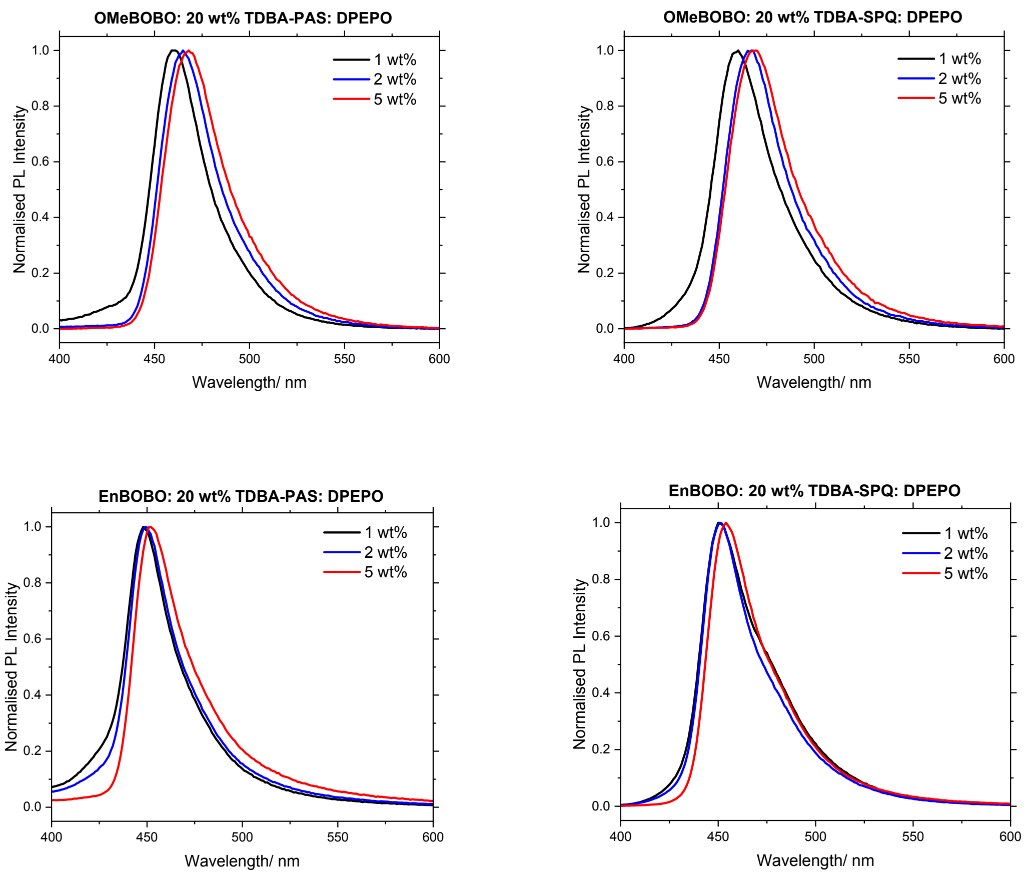

Figure S32. Photoluminescence spectra of the evaporated device films. λ_{exc} = 365 nm.

Table S5. Photoluminescence properties of the evaporated device films.

Film	λ_{PL}/ nm	FWHM/nm (eV)
**DPEPO: 20wt %TDBA-PAS**	**441**	**57 (0.32)**
1 wt% **OMeBOBO**	459	33 (0.18)
1 wt% **EnBOBO**	448	31 (0.18)
2 wt% **OMeBOBO**	465	35 (0.19)
2 wt% **EnBOBO**	449	30 (0.17)
5 wt% **OMeBOBO**	468	37 (0.19)
5 wt% **EnBOBO**	452	32 (0.18)

**DPEPO: 20 wt% TDBA-SPQ**	**459**	**60 (0.31)**
1 wt% **OMeBOBO**	460	37 (0.20)
1 wt% **EnBOBO**	450	38 (0.21)
2 wt% **OMeBOBO**	467	36 (0.19)
2 wt% **EnBOBO**	451	32 (0.18)
5 wt% **OMeBOBO**	469	39 (0.20)
5 wt% **EnBOBO**	455	33 (0.18)

The spectral overlap integrals (*J*) for the sensitisers and terminal emitters of these device films were calculated as follows:

$$J = \int F(\lambda)_D \cdot \varepsilon(\lambda)_A \cdot \lambda^4 d\lambda$$

Where $F(\lambda)_D$ is the area normalised photoluminescence spectrum of the 20 wt% sensitiser-doped DPEPO films ($\int F(\lambda)_D = 1$), and $\varepsilon(\lambda)_A$ is the molar extinction spectrum of the terminal emitters (**OMeBOBO**, **EnBOBO**) in toluene solution.

Table S6. The spectral overlap integrals (*J*) of the evaporated device films.

Sensitiser	Terminal Emitter	J / 10^{-14} nm^4 M^{-1} cm^{-1}
**TDBA-PAS**	**EnBOBO**	4.05
**TDBA-PAS**	**OMeBOBO**	4.87
**TDBA-SPQ**	**EnBOBO**	2.15
**TDBA-SPQ**	**OMeBOBO**	2.99

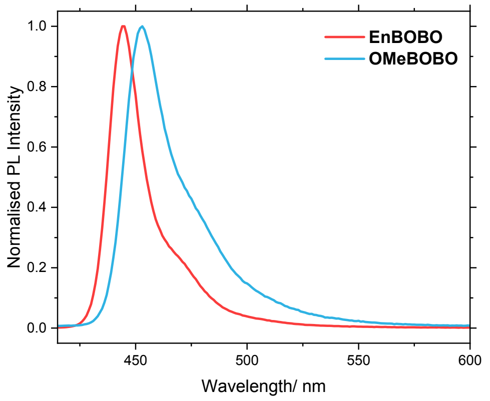

Figure S33. Photoluminescence spectra of the spin-coated ZEONEX® films. λ_{exc} = 405 nm.

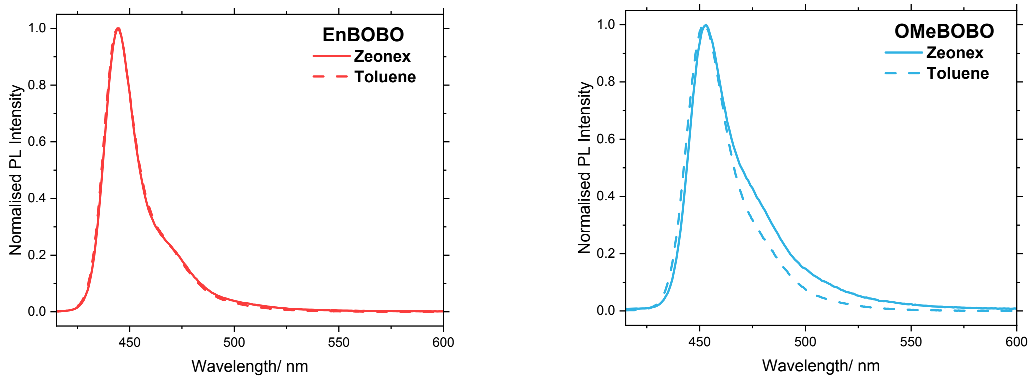

Figure S34. A comparison of photoluminescence spectra of **EnBOBO** and **OMeBOBO** when in a dilute toluene solution and when spin-coated into a ZEONEX® film at 1 wt%.

Table S7. Photoluminescence properties of the spin-coated ZEONEX® films.

	λ_{PL}/ nm	FWHM/ nm	CIExy
**1 wt% OMeBOBO**	453	26 (0.15)	(0.144, 0.088)
**1 wt% EnBOBO**	445	18 (0.11)	(0.153, 0.039)

Photoluminescence Quantum Yield (PLQY)

Toluene solutions of **OMeBOBO** and **EnBOBO** were prepared with an absorbance of no less than approximately 0.1 at the excitation wavelength (λ_{exc} = 420 nm) used for these measurements (*ca.* 5.75 μM). An Edinburgh Instruments FS5 spectrofluorometer equipped with an integrating sphere module (SC-30) and a 10 mm cuvette were employed to measure two spectra which were used to determine the PLQY (ϕ) of these solutions. Firstly, the intensity of the excitation source when passing through a cuvette of toluene in the sphere is measured. Then, both the intensity of the excitation wavelength source passing through a cuvette of the emitter solutions in the sphere and the resulting photoluminescence are recorded in the same spectrum. The quantum yield wizard of the Fluoracle® software was used to compare the change in the intensity of the excitation source as the emitter is introduced to the sphere (photons absorbed) with the resulting photoluminescence (photons emitted) to determine the raw value of ϕ. Subsequently, the raw ϕ was then corrected to account for self-reabsorption effects that occur due to the small Stokes shift of the emitters and the long pathlength created by the integrating sphere,[10] and exciton-quenching (mainly triplet excitons) effects which occur under air, as follows:

$$\phi = \frac{\phi_{raw} \cdot O_2}{1 - \alpha}$$

Where O_2 is the ratio between the total integrated photoluminescence intensity when a dilute toluene solution of the emitter is degassed by argon bubbling and when it is under air, and α is the self-reabsorption factor, which represents the probability of an emitted photon being reabsorbed by the same sample.[10] When the tail of the red-edge of the photoluminescence spectrum of the solution measured in the sphere is fitted to the photoluminescence spectrum of a dilute solution measured in a linear configuration (**Figure S35**), the ratio between the total integrated photoluminescence intensity of these two spectra is equal to $1/(1 - \alpha)$.[10]

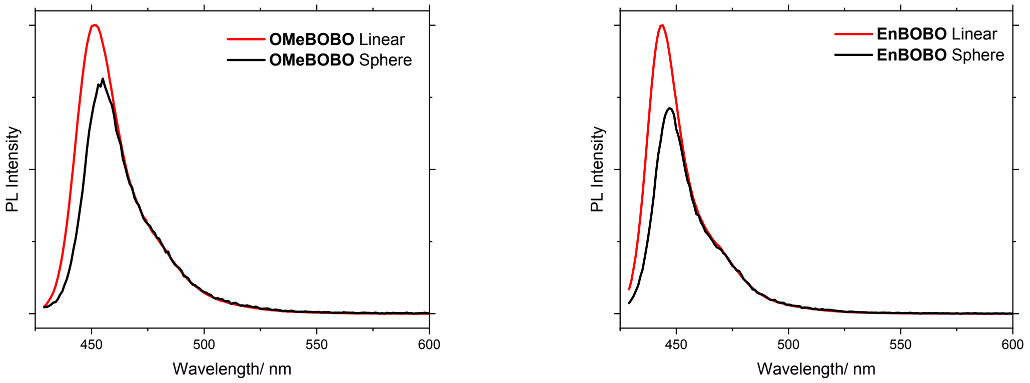

Figure S35. Photoluminescence spectra of dilute toluene solutions **OMeBOBO** and **EnBOBO** measured in a linear configuration and tail-fitted photoluminescence spectra of **OMeBOBO** and **EnBOBO** measured in an integrating sphere as part of the PLQY determination measurement.

Table S8. Summary of solution-state PLQY measurements and corrections.

Emitter	ϕ_{raw}/ %	α	ϕ^{a} / %	O_2	ϕ^{b} / %
**OMeBOBO**	54	0.187	66	1.17	78
**EnBOBO**	60	0.232	79	1.16	91

[a]Raw ϕ corrected for self-reabsorption effects. [b]Raw ϕ corrected for both self-reabsorption effects and exciton-quenching effects that occur under air.

Photoluminescence quantum yields (PLQYs) of the BOBO:ZEONEX® films were measured using a custom-built setup in the Yusuf Hamied Department of Chemistry at the University of Cambridge. These films were drop-casted, rather than spin-coated, to provide a thicker film with enough absorbance for an accurate PLQY measurement. Otherwise, all sample fabrication details are as described above.

Table S9. Summary of ZEONEX® film PLQY measurements and corrections.

Film	ϕ_{raw}/ %	ϕ^{a} / %
**1 wt% OMeBOBO**	57 ± 6	81 ± 9
**1 wt% EnBOBO**	56 ± 4	93 ± 7

[a]Raw ϕ corrected for both self-reabsorption effects. The percentage error is propagated.

Transient Photoluminescence

Quartz cuvettes with a 1 mm pathlength and graded seals (quartz to glass) were purchased from Starna® Scientific and were subsequently customised in Yusuf Hamied Department of Chemistry at the University of Cambridge by Keith Parmenter. A J Youngs tap, a glass side arm, and a glass degassing chamber were installed. In-situ sample degassing was performed on dilute solutions of **OMeBOBO** and **EnBOBO** (*ca.* 2.5 µM) using the degassing chamber of these cuvettes and five cycles of freeze-pump-thaw before they were sealed with the J Youngs tap under vacuum. This sample preparation ensures that oxygen is sufficiently excluded to prevent exciton-quenching (mainly long-lived triplets), which allows for accurate determination of photoluminescence lifetimes.

An Edinburgh Instruments FLS1000 photoluminescence spectrometer equipped with a 405 nm HPL pulsed laser diode (10 MHz) and a 405 nm EPL pulsed laser diode (10 kHz) was used to measure the photoluminescence decay of these solutions in TCSPC and MCS mode, respectively. The emission intensity was monitored at the wavelength of maximum photoluminescence intensity (λ_{PL}) observed in the steady-state spectra.

Mono-exponential decay functions were fitted to the resulting photoluminescence decays, with the TCSPC measurements giving the prompt photoluminescence decay lifetimes (τ_p) and the MCS measurements giving the delayed photoluminescence decay lifetimes (τ_d). For the latter, a significant instrument response function (IRF) was convoluted with the initial

decay of the sample. As a result, the mono-exponential function was only fitted in the time region where the measured signal was attributed to the sample (t > 4 μs). Subsequently, the resulting pre-exponential factor (I_d) was scaled to its value at t = 0 μs by extrapolating the fitted decay function. Each of the decays was numerically integrated to give the area (A) under the curve. Again, the delayed area (A_D) was scaled to its value at t = 0 μs by extrapolating the fitted decay function.

Table S10. Summary of the photoluminescence decay fitting parameters.

Emitter	τ_p/ ns	I_p	$\tau_p I_p$/ ns	A_P/ ns	τ_d/ μs	$I_d \times 10^{-4}$	$\tau_d I_d$ / μs	A_D / μs
**OMeBOBO**	5.4	1.02	5.51	5.29	41	2.8	11.28	18.74
**EnBOBO**	5.3	1.01	5.35	5.19	12	2.1	2.48	8.19

The entire photoluminescence decay curves (prompt and delayed decay components) of these solutions were also measured using a custom-built setup in the Cavendish Laboratory at the University of Cambridge, which is described as follows:

An 800 nm 1 kHz fundamental laser (Solstice® Ace) was frequency doubled using a barium borate (BBO) crystal and then passed through a 400 nm band pass filter to provide the setup with a 400 nm excitation pump. The pump power was modulated with a variable neutral density filter before being focussed with a lens onto each sample. The resulting photoluminescence was collected using a collimating lens and focussed through a 425 nm long pass filter onto the slit of an Andor iStar electrically-gated intensified charge-coupled device (ICCD) detector. The data were acquired and visualised using the Andor SOLIS for spectroscopy software with the grating of the detector centred at 450 nm. Time zero is defined

as the time-gating that gives the maximum photoluminescence intensity. The photoluminescence spectrum is accumulated at different time delays with respect to time zero over five separate overlapping time windows with twenty 5 ns, 50 ns, 500 ns, 5 us, 50 us steps between – 10 – 90 ns, 40 ns – 1 μs, 500 ns – 10 μs, 5.75 μs -105.75 μs, and 49.25 μs– 999.25 μs. Where necessary, the spectra were manually corrected for any baseline drifts and detector artefacts by subtracting the spectrum of the detector response measured in the absence of the excitation pump. The resulting spectra were integrated to yield their total photoluminescence intensity. The photoluminescence intensities measured in the separate windows were scaled appropriately using overlapping data points to stitch the windows together and give the entire photoluminescence decay curve.

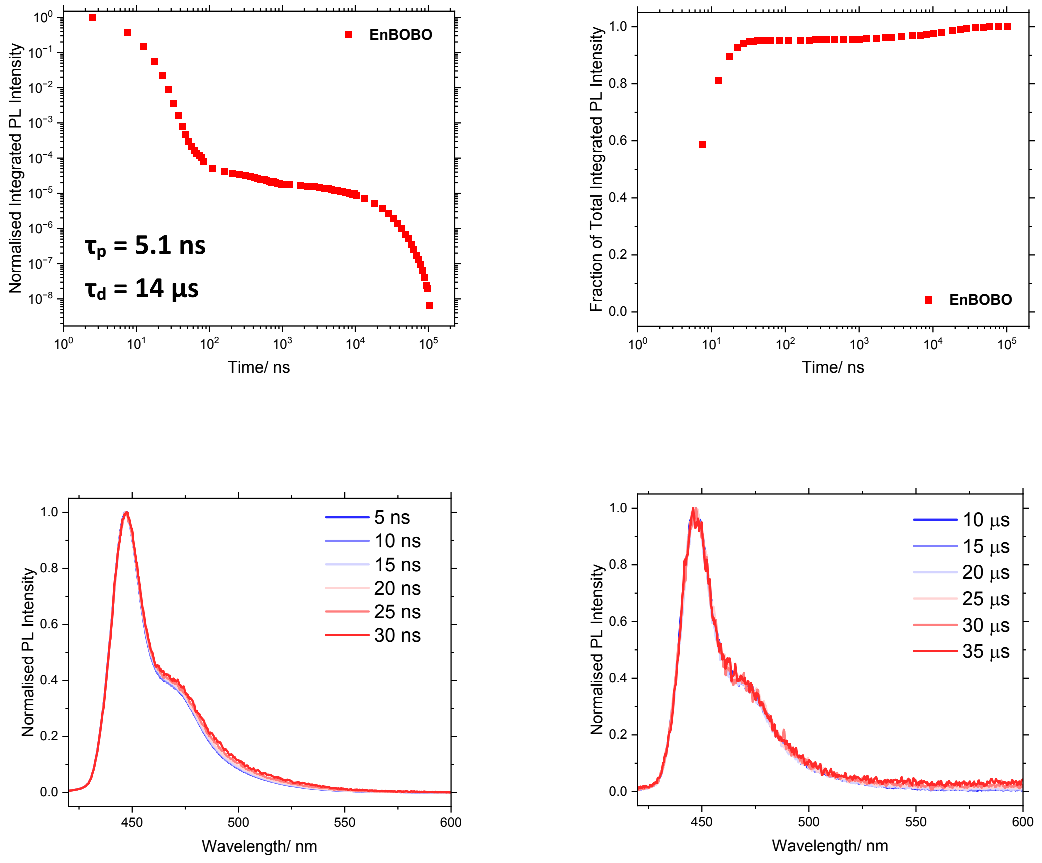

Figure S36. The photoluminescence decay behavior of a dilute toluene solution of **EnBOBO.**

The prompt and delayed portions of the photoluminescence decay curve were numerically

integrated and from their ratio the delayed:prompt photoluminescence ratio (ϕ_d/ϕ_p) was determined.

Key photophysical rate constants for these thermally-activated delayed fluorescence emitters were determined according to a previously reported method,[11] which assumes there is negligible non-radiative decay from the first singlet excited state (S_1) to the singlet ground state (S_0) and negligible radiative decay from the first triplet excited state (T_1) to the singlet ground state (S_0):

OMeBOBO

τ_p	5.4 ns
τ_d	41 μs
ϕ_p	0.70
$\frac{\phi_d}{\phi_p}$	0.12
$k_p = \frac{1}{\tau_p}$	1.9×10^8 s^{-1}
$k_d = \frac{1}{\tau_d}$	2.4×10^4 s^{-1}
$k_r = \frac{\phi_P}{\tau_p}$	1.3×10^8 s^{-1}
$k_{ISC} = k_p(1 - \phi_p)$	5.7×10^7 s^{-1}
$k_{RISC} = \frac{k_p k_d}{k_{ISC}} \left(\frac{\phi_d}{\phi_p}\right)$	9.6×10^3 s^{-1}

EnBOBO

τ_p	5.3 ns
τ_d	12 µs
ϕ_p	0.87
$\frac{\phi_d}{\phi_p}$	0.05
$k_p = \frac{1}{\tau_p}$	1.9×10^8 s^{-1}
$k_d = \frac{1}{\tau_d}$	8.3×10^4 s^{-1}
$k_r = \frac{\phi_P}{\tau_p}$	1.6×10^8 s^{-1}
$k_{ISC} = k_p(1 - \phi_p)$	2.5×10^7 s^{-1}
$k_{RISC} = \frac{k_p k_d}{k_{ISC}} \left(\frac{\phi_d}{\phi_p}\right)$	3.2×10^4 s^{-1}

The entire photoluminescence decay curves (prompt and delayed decay components) of the device films were also measured using a custom-built setup in the Cavendish Laboratory at the University of Cambridge, which is as previously described apart from the following:

A 1030 nm 10 kHz fundamental laser (Light Conversion Pharos) was inputted into a tuneable collinear optical parametric amplifier (Light Conversion Orpheus) to provide the setup with a 365 nm excitation pump wavelength. This wavelength was chosen to selectively excite the sensitiser component of the films. The resulting photoluminescence was passed through a 375 nm long pass filter. The grating of the detector was centred at 450 nm. The photoluminescence spectra were measured over six separate overlapping time windows with twenty 5 ns, 30 ns, 150 ns, 500 ns, 3 µs steps and seven 10 µs steps between –10 – 90 ns, -10 – 590 ns, 140 ns – 3.14 µs, 1.64 – 11.64 µs, 2.64 µs – 62,64 µs, and 29.99 µs – 99.99 µs with respect to time

zero.

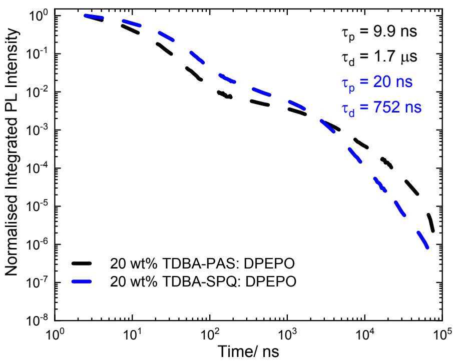

τ_p = 9.9 ns
τ_d = 1.7 μs
τ_p = 20 ns
τ_d = 752 ns
20 wt% TDBA-PAS: DPEPO
20 wt% TDBA-SPQ: DPEPO
Normalised Integrated PL Intensity
Time/ ns

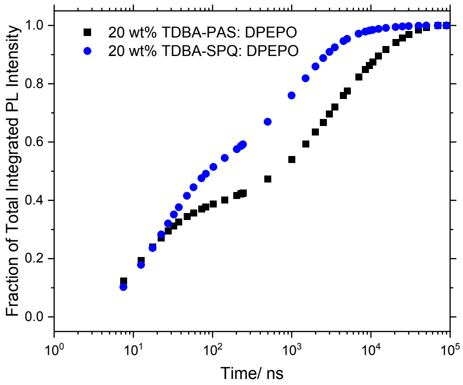

20 wt% TDBA-PAS: DPEPO
20 wt% TDBA-SPQ: DPEPO
Fraction of Total Integrated PL Intensity
Time/ ns

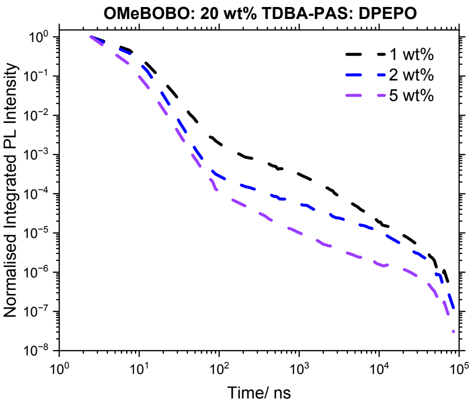

OMeBOBO: 20 wt% TDBA-PAS: DPEPO
1 wt%
2 wt%
5 wt%
Normalised Integrated PL Intensity
Time/ ns

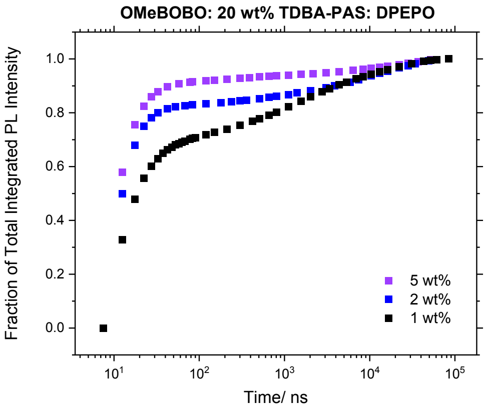

OMeBOBO: 20 wt% TDBA-PAS: DPEPO
5 wt%
2 wt%
1 wt%
Fraction of Total Integrated PL Intensity
Time/ ns

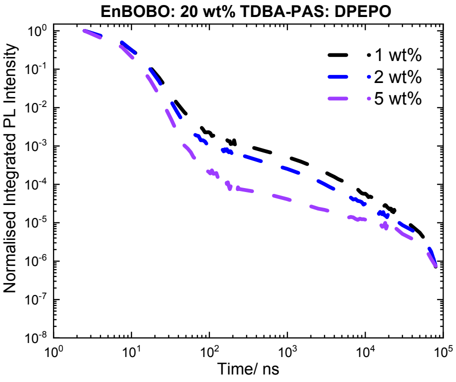

EnBOBO: 20 wt% TDBA-PAS: DPEPO
1 wt%
2 wt%
5 wt%
Normalised Integrated PL Intensity
Time/ ns

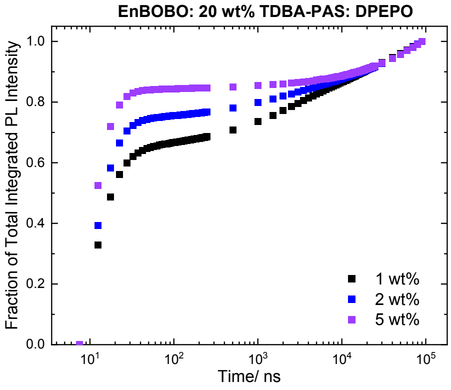

EnBOBO: 20 wt% TDBA-PAS: DPEPO
1 wt%
2 wt%
5 wt%
Fraction of Total Integrated PL Intensity
Time/ ns

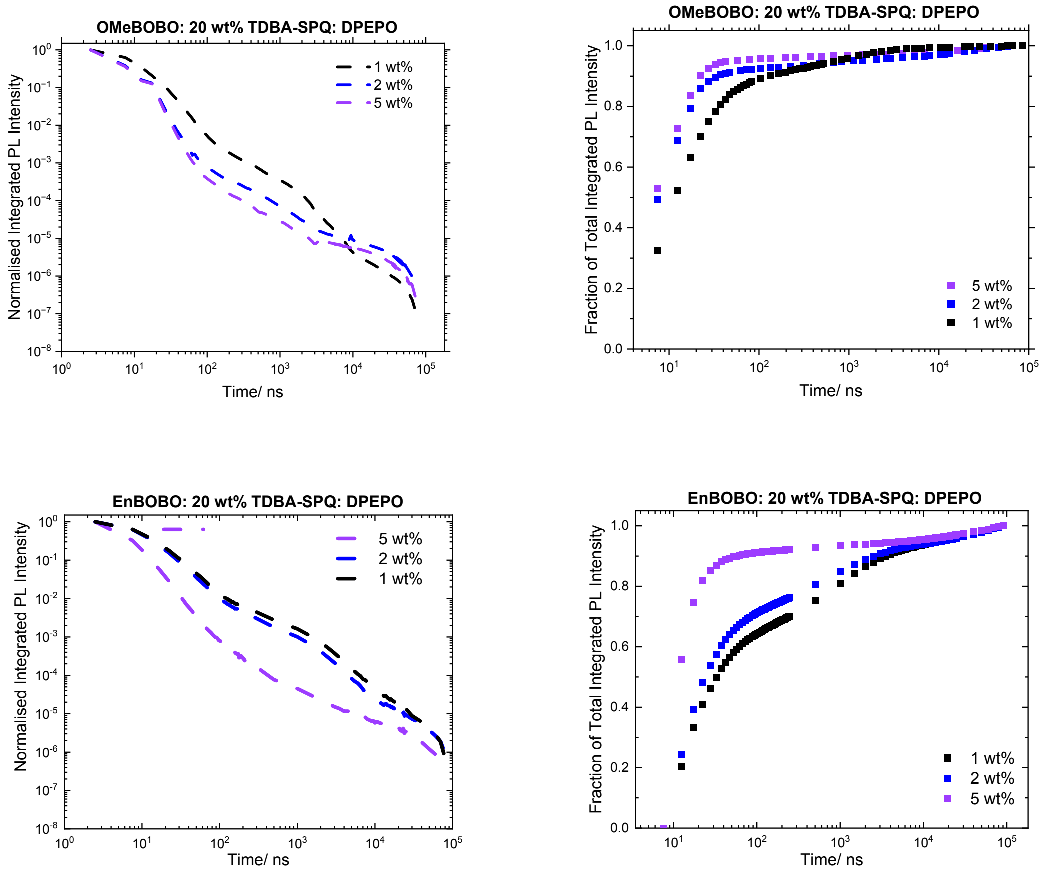

Figure S37. The photoluminescence decay behavior of the device films.

Table S11. Summary of the photoluminescence decay fitting parameters. *For non-doped device films: τ_d = fitted mono-exponential lifetime. For doped device films: $\tau_d = \frac{\sum A_i \tau_i^2}{\sum A_i \tau_i}$ which is determined from a tri-exponential fit of the delayed component.

Film	τ_p/ ns	τ_d*/ µs	ϕ_d/ϕ_p
**20 wt% TDBA-PAS: DPEPO**	9.9	1.7	1.4
1 wt% OMeBOBO: 20 wt% TDBA-PAS: DPEPO	6.3	2.5	0.19
2 wt% OMeBOBO: 20 wt% TDBA-PAS: DPEPO	5.1	8.1	0.076
5 wt% OMeBOBO: 20 wt% TDBA-PAS: DPEPO	3.1	0.14	0.021
1 wt% EnBOBO: 20 wt% TDBA-PAS: DPEPO	6.8	4.6	0.38
2 wt% EnBOBO: 20 wt% TDBA-PAS: DPEPO	6.8	5.9	0.21
5 wt% EnBOBO: 20 wt% TDBA-PAS: DPEPO	5.1	1.2	0.091
**20 wt% TDBA-SPQ: DPEPO**	20	0.75	0.82
1 wt% OMeBOBO: 20 wt% TDBA-SPQ: DPEPO	9.5	0.66	0.11
2 wt% OMeBOBO: 20 wt% TDBA-SPQ: DPEPO	5.6	9.5	0.078
5 wt% OMeBOBO: 20 wt% TDBA-SPQ: DPEPO	4.5	0.011	0.042
1 wt% EnBOBO: 20 wt% TDBA-SPQ: DPEPO	11	0.13	0.44
2 wt% EnBOBO: 20 wt% TDBA-SPQ: DPEPO	10	0.62	0.31
5 wt% EnBOBO: 20 wt% TDBA-SPQ: DPEPO	4.5	0.55	0.06

The photoluminescence decay curves of the spin-coated BOBO:ZEONEX® films were measured using an Edinburgh Instruments FLS1000 photoluminescence spectrometer equipped with a 405 nm HPL pulsed laser diode (20 MHz) and a 400 nm VPL pulsed laser diode (5 kHz), in TCSPC and MCS mode, respectively. The emission intensity was monitored at the wavelength of maximum photoluminescence intensity (λ_{PL}) observed in the steady-state spectra.

Table S12. Summary of the photoluminescence decay fitting parameters for the ZEONEX® films.

	τ_p / ns	τ_d / µs
**1 wt% OMeBOBO**	5.0	76
**1 wt% EnBOBO**	5.1	87

Low-temperature Measurements

A film where 1 wt% of **EnBOBO** is doped into ZEONEX® was prepared by drop-casting the appropriate toluene solution of **EnBOBO** and ZEONEX® (20 mg mL^{-1}).

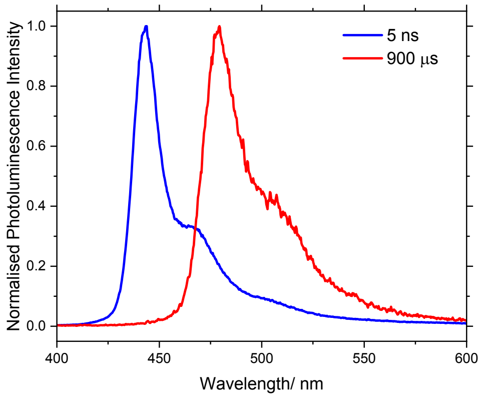

Figure S38. The low-temperature (10 K) normalized photoluminescence spectra at 5 ns and 900 µs. λ_{exc} = 375 nm. From the onset of these spectra the energy gap between the first singlet excited state (S_1) and the first triplet excited state (T_1) (ΔE_{ST}) is 0.19 eV.

Angle-dependent Photoluminescence Measurements

30 nm-thick device films were thermally evaporated for these measurements. Each film was photoexcited using 325 nm. The photoluminescence intensity refers to the intensity at peak maximum of the photoluminescence spectrum. This intensity is normalised to the value measured at 0°.

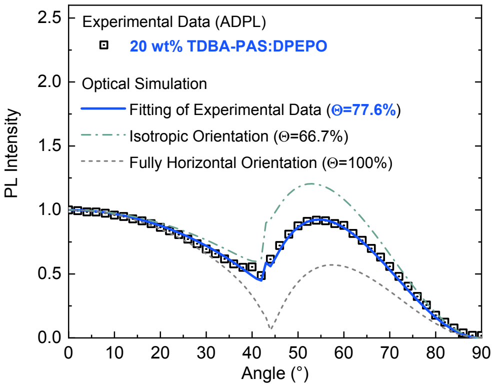

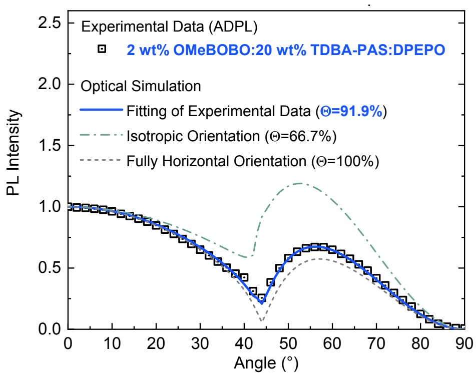

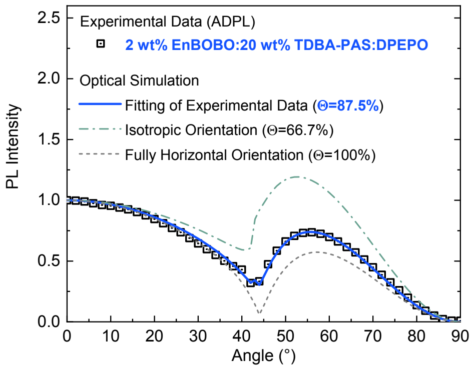

Figure S39. Angle-dependent photoluminescence (ADPL) plots for the TDBA-PAS device films. The simulated ADPL for an emitter with an isotroptic and fully-horizontal orientation are included. The experimental data is fitted to give the orientation, θ, as shown.

S8. Computational Details

Theoretical calculations were performed using the Gaussian 16 package.[12] The geometry of **OMeBOBO** and **EnBOBO** were optimized using the density functional theory (DFT) method in the gas phase using the B3LYP functional,[13,14] and the 6-31G(d,p) basis set.[15,16] Dispersion interactions were accounted for using Grimme's D3 method with the BJ damping function.[17] Calculation of the vibrational frequencies of this optimised structure using the same level of theory didn't reveal any imaginary or negative frequencies, which confirmed that the structure represents an energy minima. Time-dependent DFT (TD-DFT) calculations were performed using this optimised geometry and the same level of theory to investigate the first five singlet excited states and the first five triplet excited states. Multiwfn (Version 3.8) was used for wavefunction analysis,[18] including hole-electron density analysis,[19,20] according to the program manual. Visual Molecular Dynamics (VMD) 1.9.3 software was used to visualise and create plots of the geometry optimised structures superimposed with wavefunction information.[21] Spin-orbit coupling (SOC) matrix elements between the singlet and triplet excited states were calculated from the geometry optimised T_1 state at the same level of theory using ORCA 5.0.1.

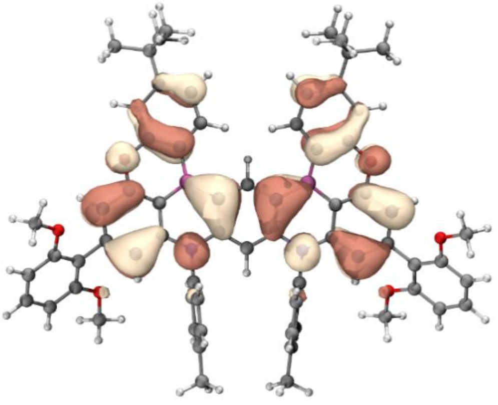

HOMO

-4.70 eV

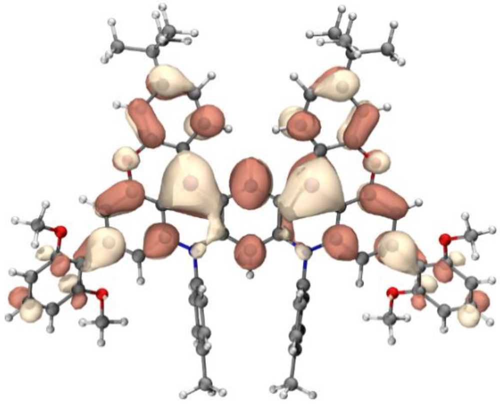

LUMO

-1.28 eV

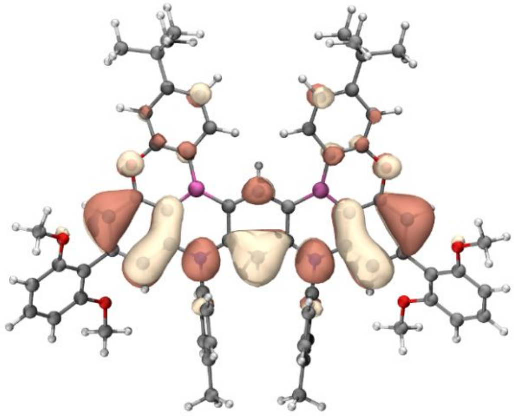

HOMO-1

-4.77 eV

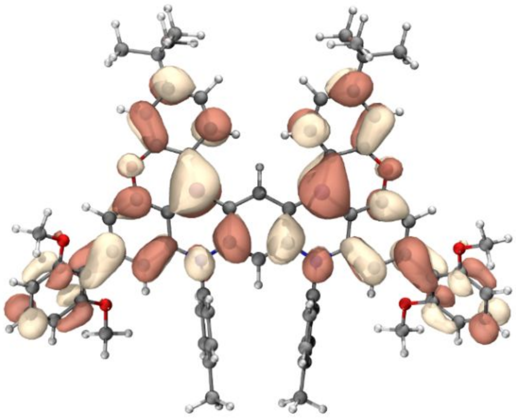

LUMO+1

-0.76 eV

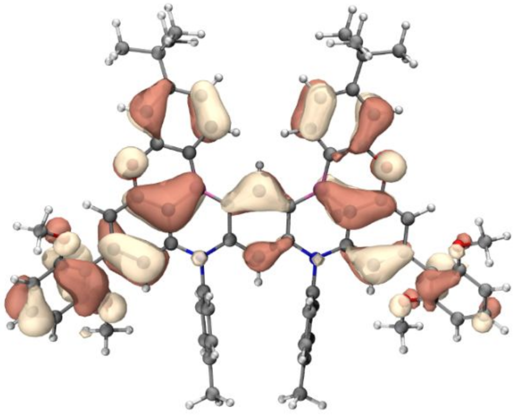

HOMO-2

-5.50 eV

Figure S40. Kohn-Sham molecular orbitals of **OMeBOBO** and their associated energies.

Table S13. A summary of results from the TD-DFT calculation for **OMeBOBO** and **EnBOBO.**

	**OMeBOBO**		**EnBOBO**		
State	Energy/ eV (nm)	Oscillator Strength	Energy/ eV (nm)	Oscillator Strength	Character (**OMeBOBO**)
T_1	2.57	0	2.59	0	HOMO ─ LUMO: 90%<br>HOMO-1 ─ LUMO+1: 7%
T_2	2.61	0	2.61	0	HOMO-1 ─ LUMO: 87%
S_1	2.95 (421)	0.423	2.96 (420)	0.308	HOMO ─ LUMO: 95%
T_3	2.98	0	3.00	0	HOMO ─ LUMO+1: 57%<br>HOMO-2 ─ LUMO: 20%
S_2	2.98 (416)	0.085	2.98 (416)	0.125	HOMO-1 ─ LUMO: 98%
T_4	3.13	0	3.18	0	HOMO-3 ─ LUMO: 34%<br>HOMO-1 ─ LUMO+1: 19%<br>HOMO-2 ─ LUMO+1: 10%
T_5	3.21	0	3.20	0	HOMO-1 ─ LUMO+1: 60%<br>HOMO-1 ─ LUMO+8: 6%

S_3	3.49 (355)	0.169	3.51 (354)	0.158	HOMO – LUMO+1: 98%
S_4	3.57 (347)	0.239	3.57 (347)	0.215	HOMO-1 – LUMO+1: 95%
S_5	3.69 (336)	0.009	3.75 (330)	0.100	HOMO-1 – LUMO+2: 58%<br>HOMO – LUMO+2: 15%<br>HOMO-1 – LUMO+3: 11%<br>HOMO – LUMO+3: 7%

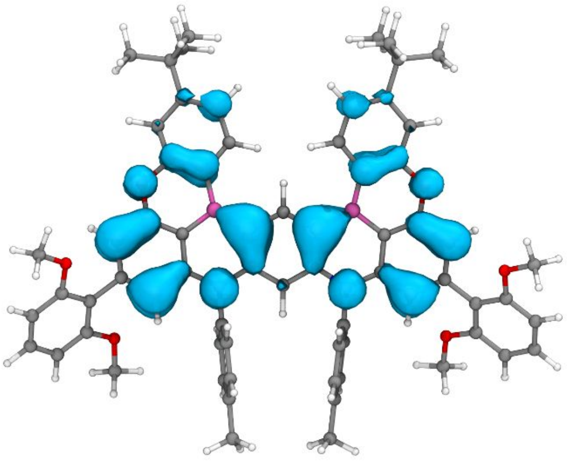

T_1 hole

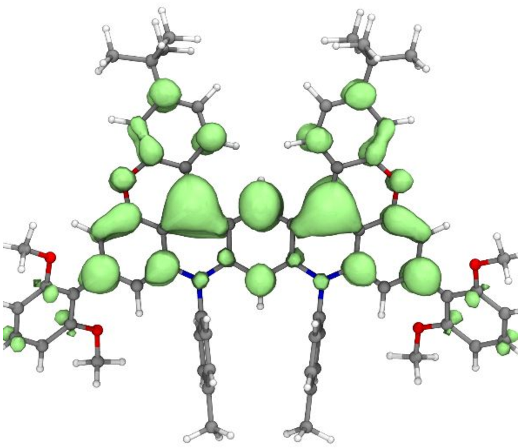

T_1 electron

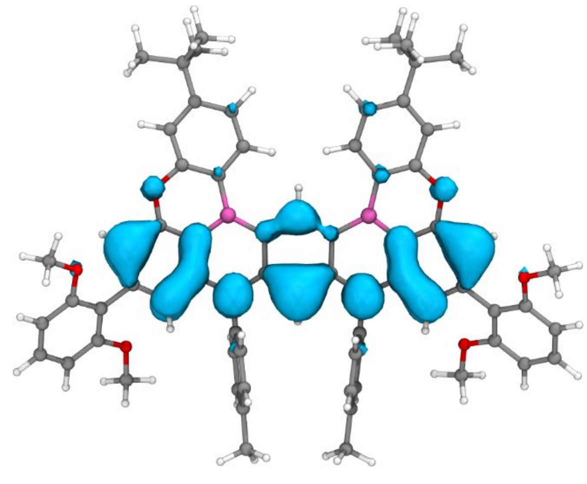

T_2 hole

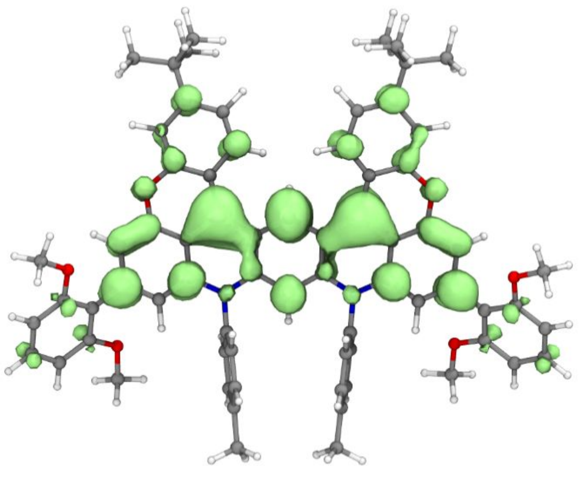

T_2 electron

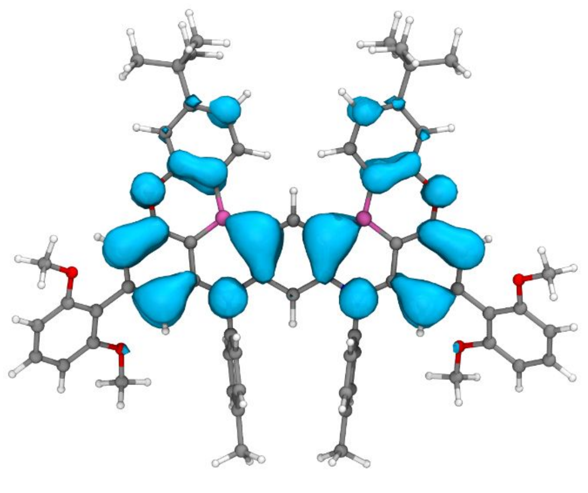

S_1 hole

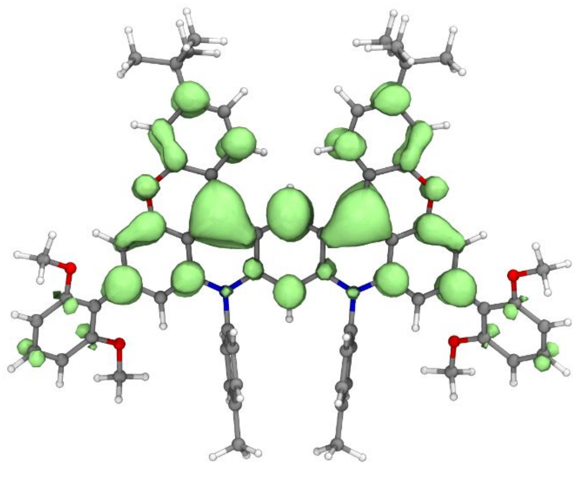

S_1 electron

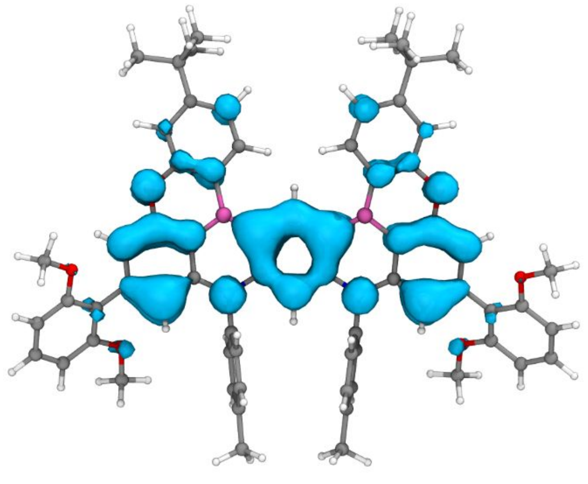

T_3 hole

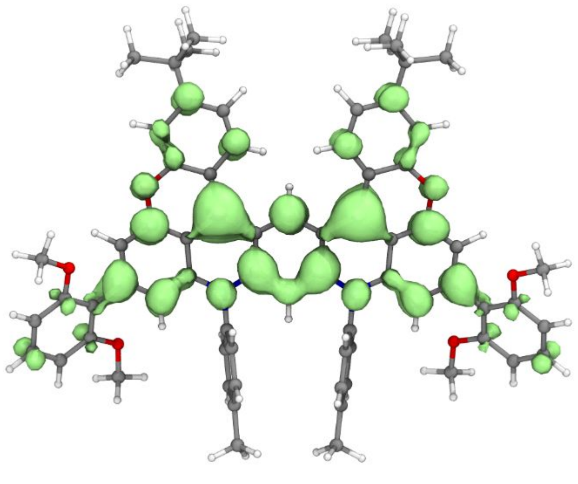

T_3 electron

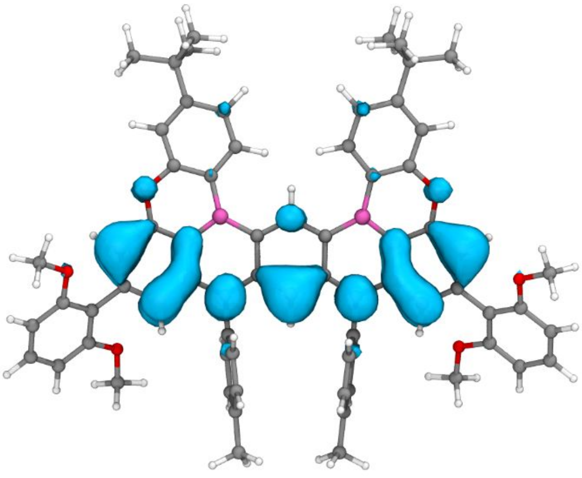

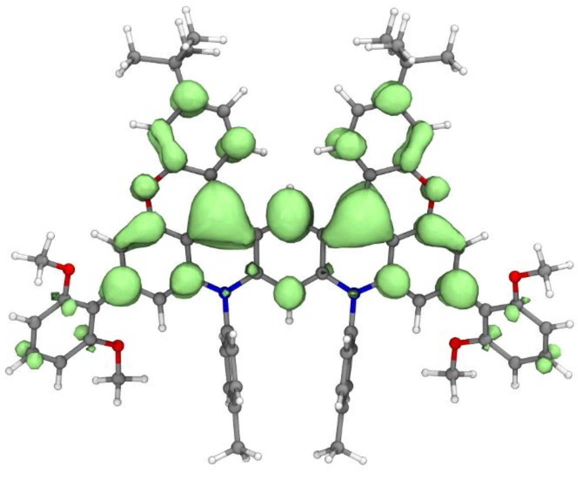

S_2 hole **S_2 electron**

Figure S41. The hole and electron density for the first five excited states of **OMeBOBO.**

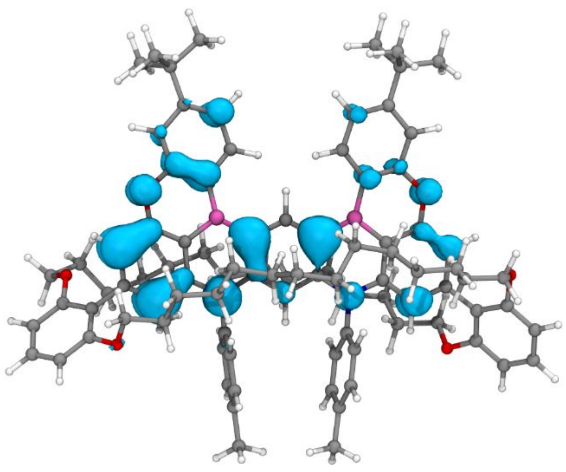

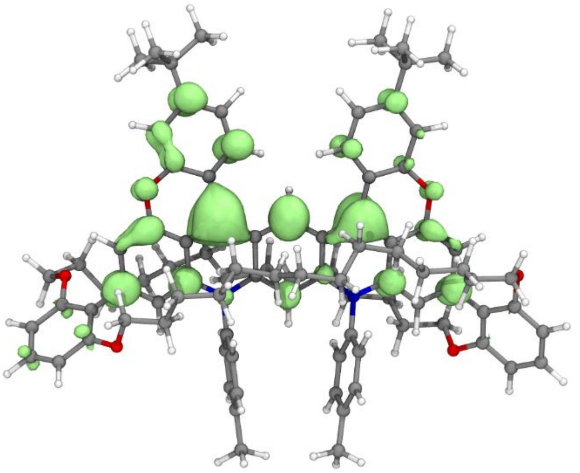

T_1 hole **T_1 electron**

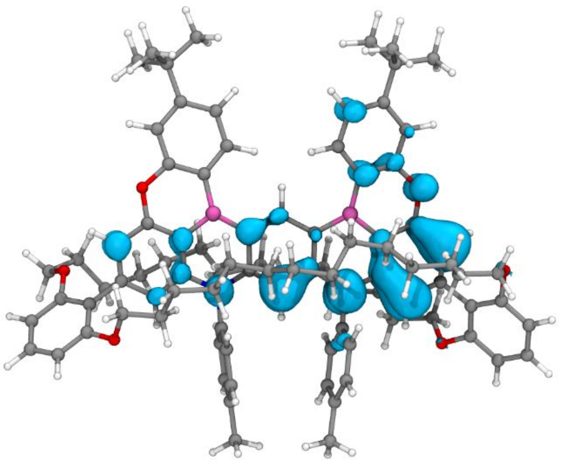

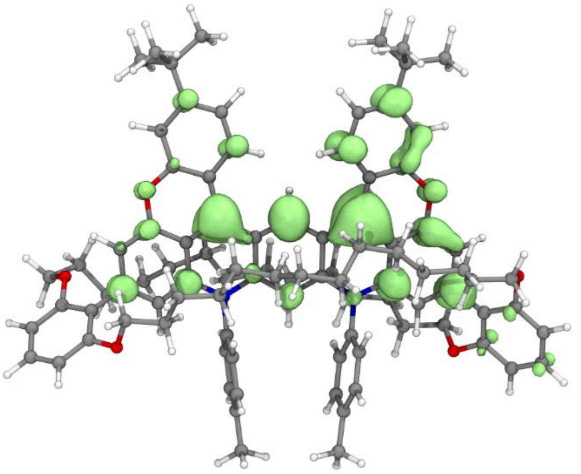

T_2 hole **T_2 electron**

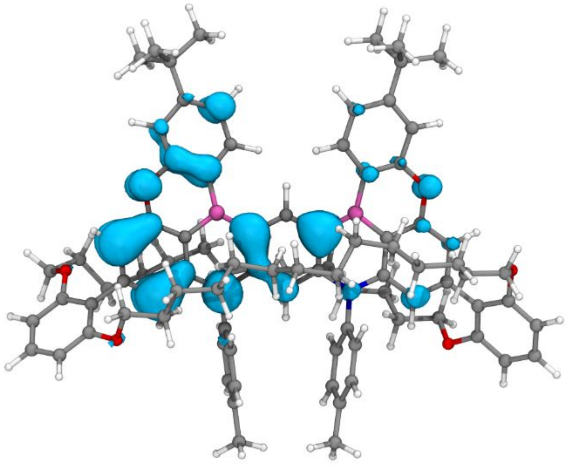
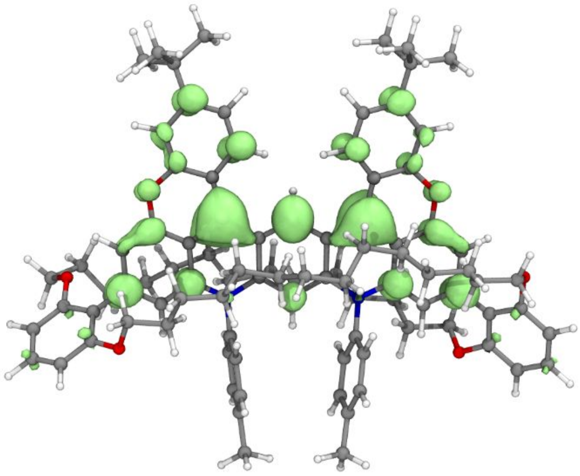

S_1 hole **S_1 electron**

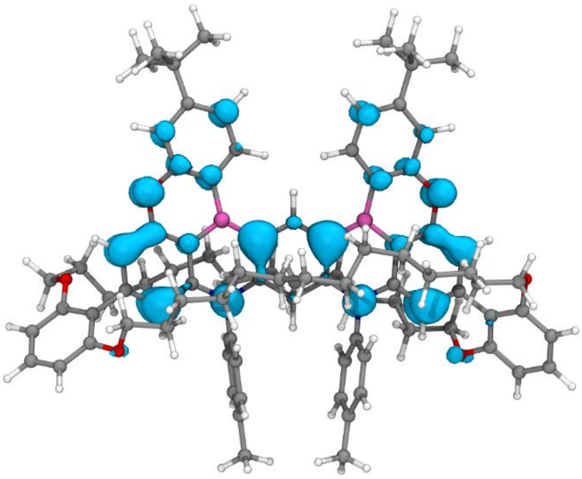
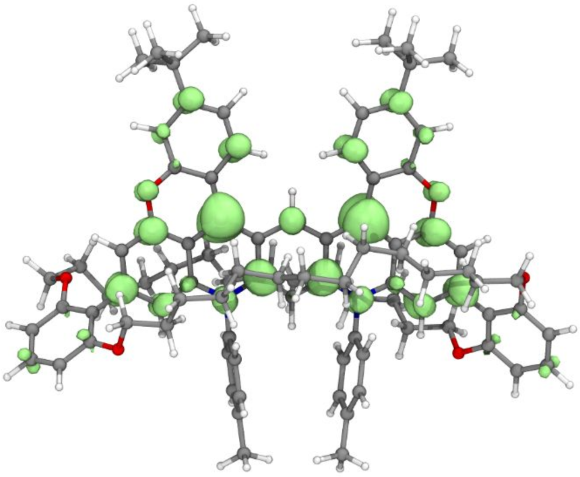

T_3 hole **T_3 electron**

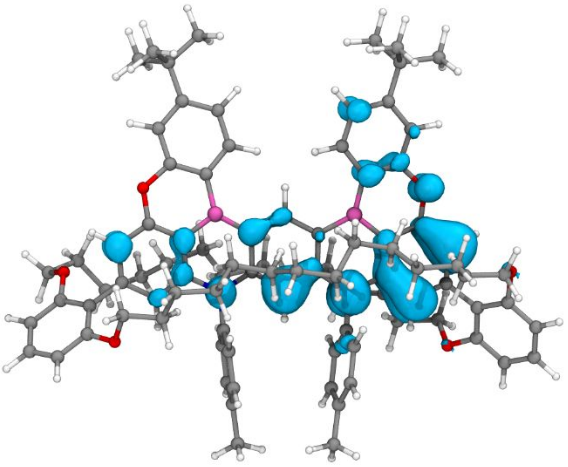
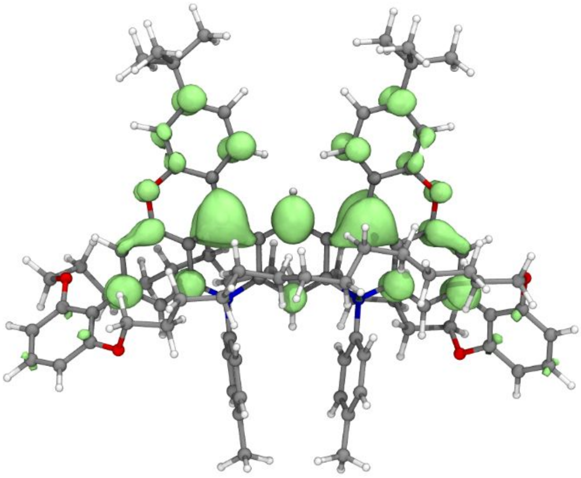

S_2 hole **S_2 electron**

Figure S42. The hole and electron density for the first five excited states of **EnBOBO.**

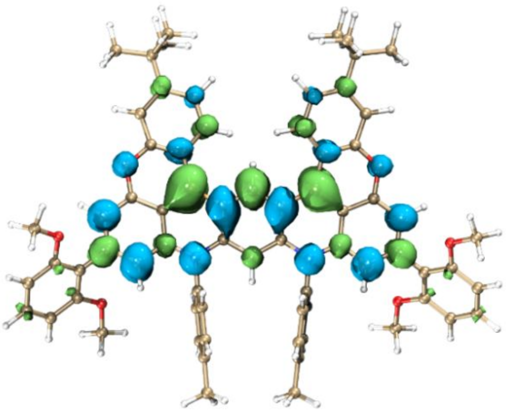

T_1

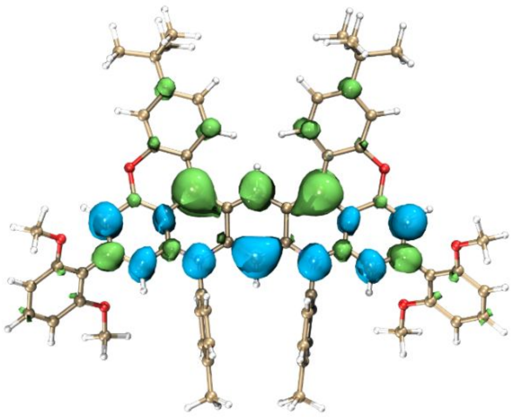

T_2

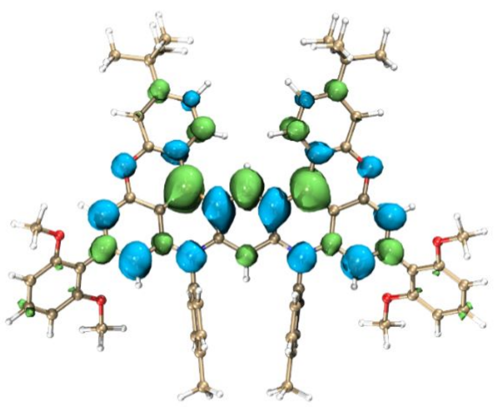

S_1

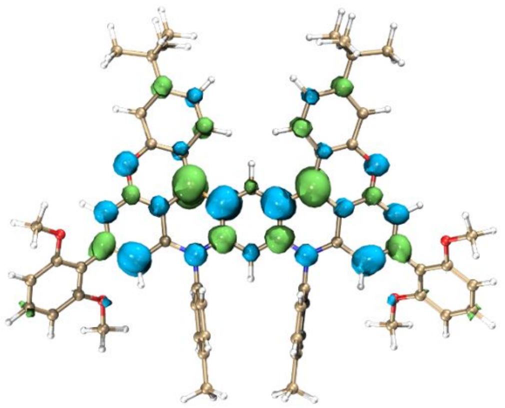

T_3

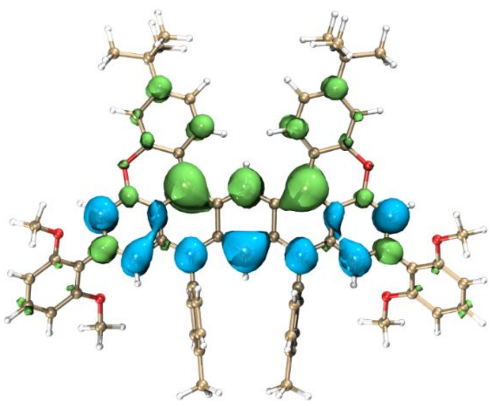

S_2

Figure S43. The charge-difference density plots of the first five excited states of

OMeBOBO.

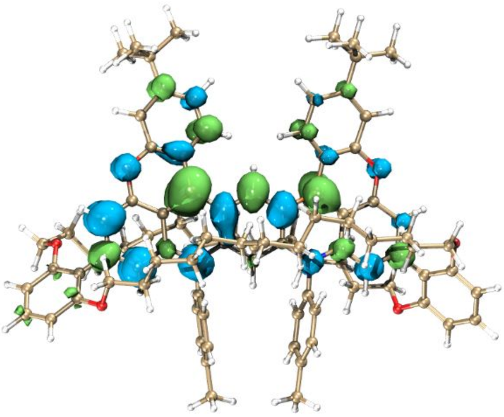

$\mathbf{T_1}$

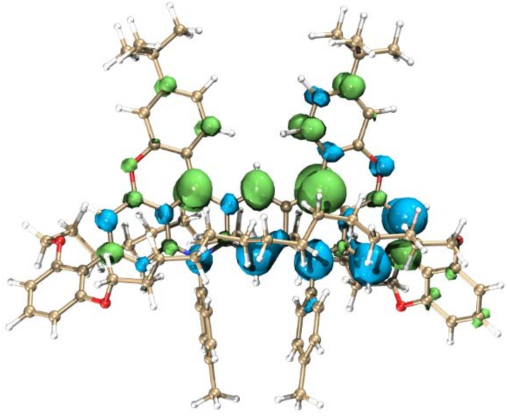

$\mathbf{T_2}$

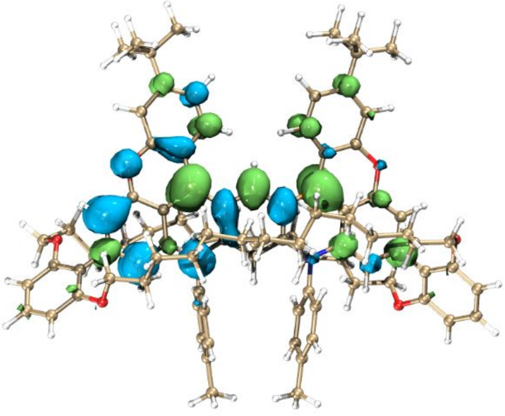

$\mathbf{S_1}$

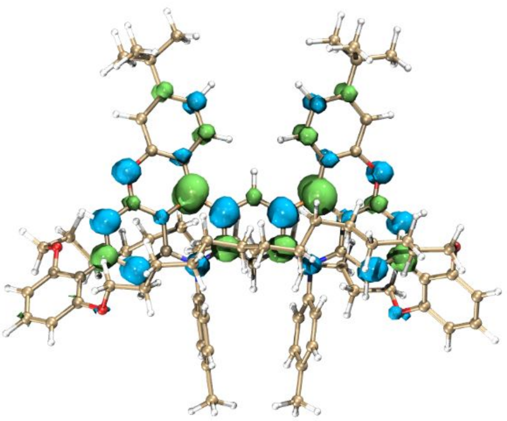

$\mathbf{T_3}$

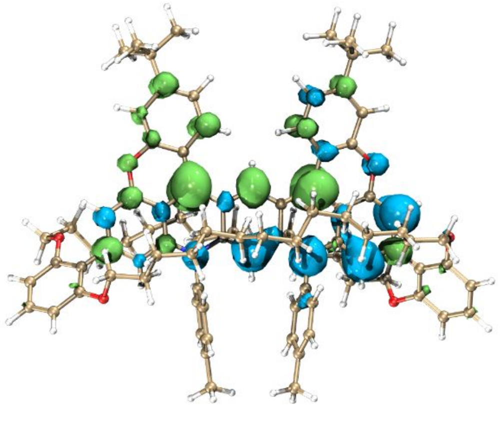

$\mathbf{S_2}$

Figure S44. The charge-difference density plots of the first five excited states of **EnBOBO.**

Table S14. A summary of parameters from the hole-electron analysis of the TD-DFT calculation of **OMeBOBO** and **EnBOBO**

State	D_{index}[a]/ Å	S_r[b]/ a.u.	t_{index}[c]/ Å
T_1 (OMeBOBO)	0.586	0.613	-1.696
T_2(OMeBOBO)	1.521	0.543	-0.544
S_1(OMeBOBO)	0.556	0.601	-1.725
T_3(OMeBOBO)	0.418	0.618	-3.078
S_2(OMeBOBO)	1.599	0.545	-0.431
T_1 (EnBOBO)	0.957	0.595	-2.133
T_2(EnBOBO)	1.770	0.541	-0.934
S_1(EnBOBO)	2.200	0.591	-1.335
S_2(EnBOBO)	2.597	0.536	-0.673
T_3(EnBOBO)	0.180	0.629	-4.051

[a]$D_{index} = \sqrt{(D_x)^2 + (D_y)^2 + (D_z)^2}$ which is the total magnitude of the charge-transfer length. D_x, D_y and D_z are the distance between the hole and electron centroids along the x,y,z directions, respectively. D_{index} values increase as the charge-transfer character of the excited state increases. [b]$S_r = \int \sqrt{\rho_{hole}(\boldsymbol{r})\rho_{electron}(\boldsymbol{r})}d\boldsymbol{r}$ which represents the hole-electron density overlap. S_r values approach zero as the charge-transfer character of the excited state increases. cSeparation of hole-electron in the direction of charge-transfer. [c]$t_{index} = D_{index} - H_{CT}$ where H_{CT} is the average spatial extension degree of the hole-electron densities in the charge-transfer direction. Negative t_{index} values represent hole-electron densities which are not substantially separated by charge-transfer.

Table S15. The spin-orbit coupling matrix elements between S_1 and T_n states for **EnBOBO** and **OMeBOBO**.

	**EnBOBO**	**OMeBOBO**
S_1-T_1	0.045	0.020
S_1-T_2	0.244	0.200
S_1-T_3	0.194	0.155
S_1-T_4	0.294	0.205
S_1-T_5	0.203	0.108

S9. OLED Devices

For the fabrication of OLED devices, indium tin oxide (ITO) coated substrates (~15 Ω/cm^2) were cleaned with acetone and isopropyl alcohol and then O_2 plasma treatment was applied to align their energy level with the hole-transporting layer. All layers, including organic layers and a LiF/aluminium cathode, were thermally deposited under high vacuum (~10^{-7} torr). The anode was ITO modified with molybdenum oxide (MoO_3) at the layer interface to facilitate injection into a hole-injection layer (HIL) of 1,1-bis[(di-4-tolylamino)phenyl]cyclohexane (TAPC). Holes were transported through the HIL into a hole-transporting layer (HTL) composed of a layer of tris(4-carbazoyl-9-ylphenyl)amine (TCTA) followed by a layer of 1,3-bis(*N*-carbazolyl)benzene (mCP). Electrons were injected from an aluminium cathode, modified with lithium fluoride (LiF), into an electron-injection layer (EIL) of 2,2',2''-(1,3,5-benzinetriyl)-tris(1-phenyl-1-*H*-benzimidazole) (TPBi). Electrons were transported through the EIL into an electron-transporting layer (ETL) of bis[2-(diphenylphosphino)phenyl]ether oxide (DPEPO).

The doping concentrations applied in the devices are weight percentages. The performance of the OLED devices was measured using a Keithley 2635 source-meter and a calibrated silicon photodiode. The electroluminescence spectra were recorded using an Ocean Optics Flame spectrometer.

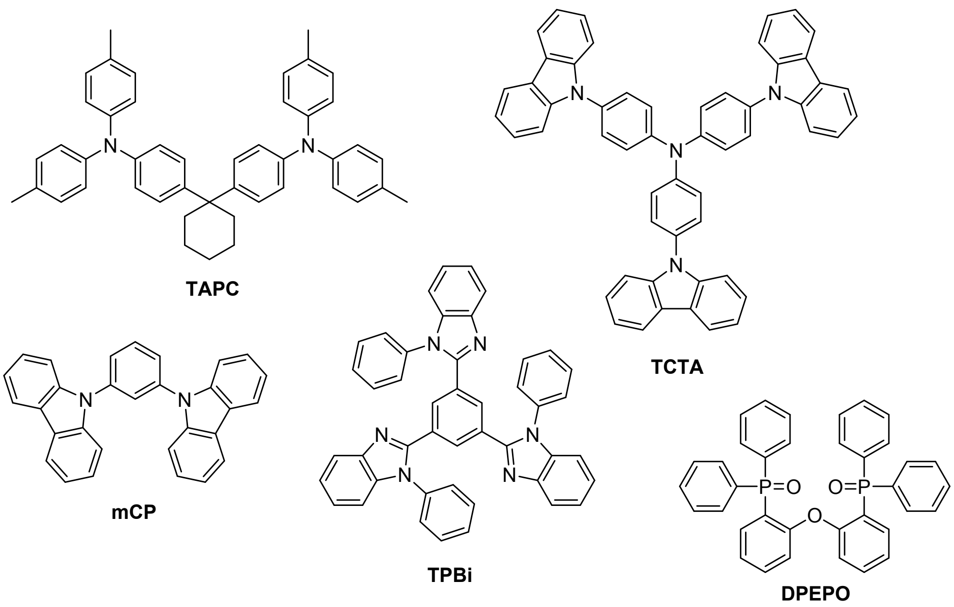

Figure S45. The chemical structures of the materials used to fabricate the different layers of the OLED devices.

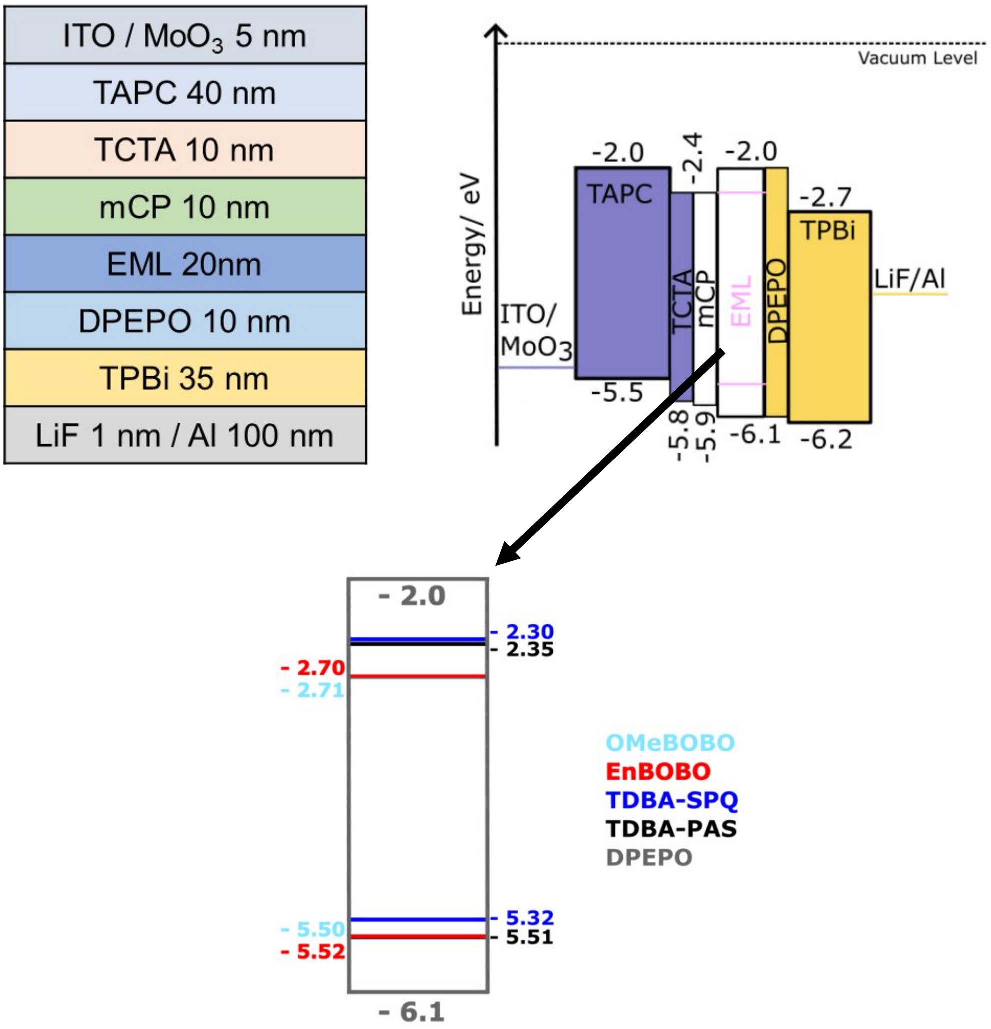

Figure S46. The architecture of the OLED devices and the energy levels of each layer and the energy levels within the emissive layer.

Table S16. A summary of OLED device performance metrics.

Device	V_{ON}[a]/ V	EQE_{max}[b]/ %	EQE_{100}[c]/ %	λ_{EL}[d]/ nm	FWHM[e]/ nm (eV)	CIE_{xy}[f]
**20 wt% TDBA-PAS: DPEPO**						
1 wt% **EnBOBO**	3.8	27.2	19	447	32	0.151, 0.042
5 wt% **EnBOBO**	3.7	27.8	14	455	29	0.142, 0.056
1 wt% **OMeBOBO**	3.9	18.5	13	457	33	0.144, 0.071
5 wt% **OMeBOBO**	3.6	21.2	13	465	34	0.129, 0.120
**20 wt% TDBA-SPQ: DPEPO**						
1 wt% **EnBOBO**	3.4	34.2	29	452	37	0.144, 0.077
5 wt% **EnBOBO**	3.4	28.8	22	454	34	0.143, 0.070
1 wt% **OMeBOBO**	3.3	34.8	29	459	35	0.138, 0.089
5 wt% **OMeBOBO**	3.3	29.5	22	467	36	0.129, 0.131

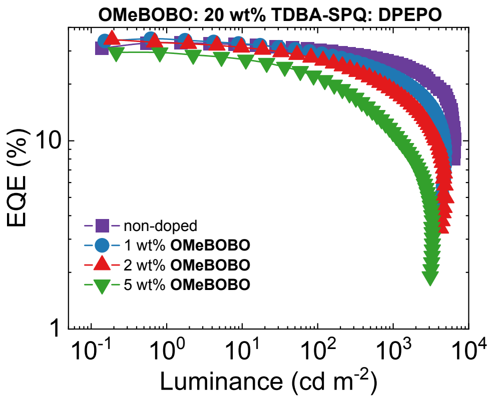
OMeBOBO: 20 wt% TDBA-SPQ: DPEPO
EQE (%)
Luminance (cd m-2)
non-doped
1 wt% OMeBOBO
2 wt% OMeBOBO
5 wt% OMeBOBO

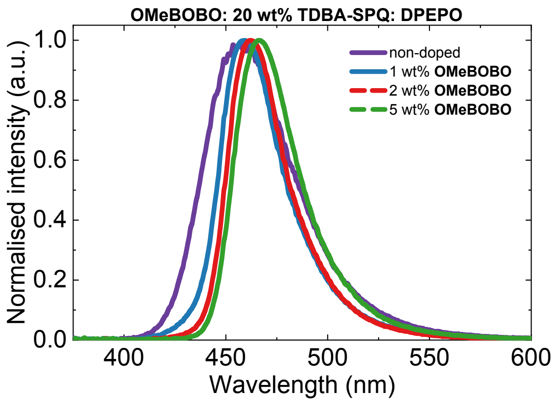
OMeBOBO: 20 wt% TDBA-SPQ: DPEPO
Normalised intensity (a.u.)
Wavelength (nm)
non-doped
1 wt% OMeBOBO
2 wt% OMeBOBO
5 wt% OMeBOBO

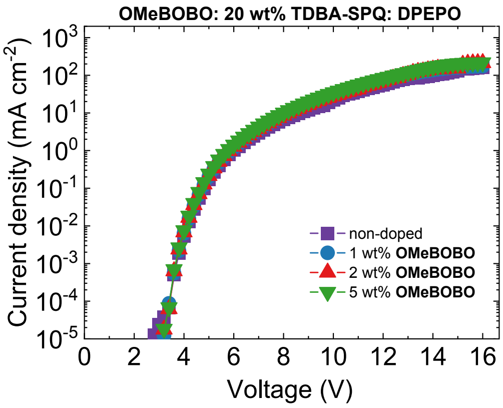
OMeBOBO: 20 wt% TDBA-SPQ: DPEPO
Current density (mA cm-2)
Voltage (V)
non-doped
1 wt% OMeBOBO
2 wt% OMeBOBO
5 wt% OMeBOBO

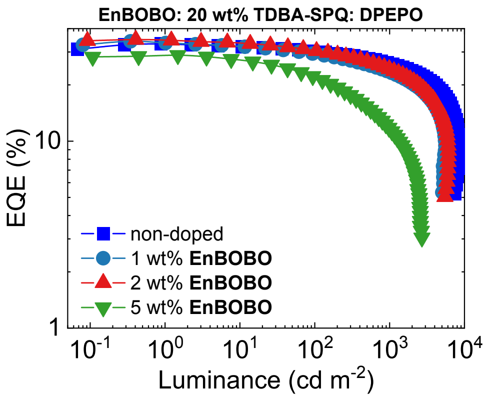
EnBOBO: 20 wt% TDBA-SPQ: DPEPO
EQE (%)
Luminance (cd m-2)
non-doped
1 wt% EnBOBO
2 wt% EnBOBO
5 wt% EnBOBO

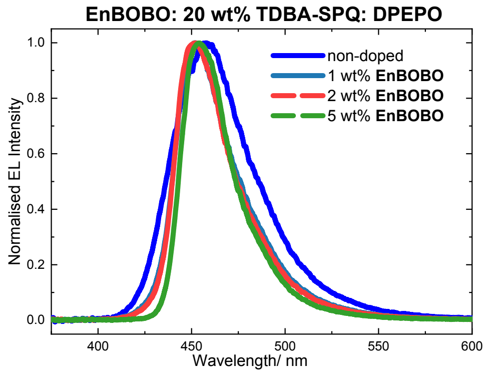
EnBOBO: 20 wt% TDBA-SPQ: DPEPO
Normalised EL Intensity
Wavelength/ nm
non-doped
1 wt% EnBOBO
2 wt% EnBOBO
5 wt% EnBOBO

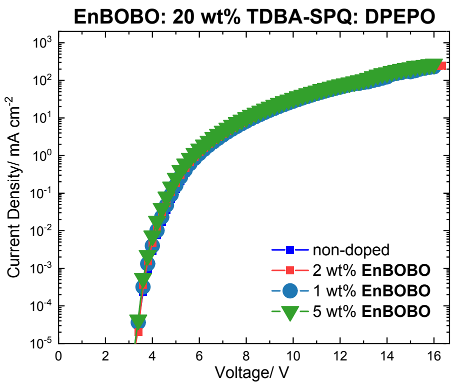
EnBOBO: 20 wt% TDBA-SPQ: DPEPO
Current Density/ mA cm-2
Voltage/ V
non-doped
2 wt% EnBOBO
1 wt% EnBOBO
5 wt% EnBOBO

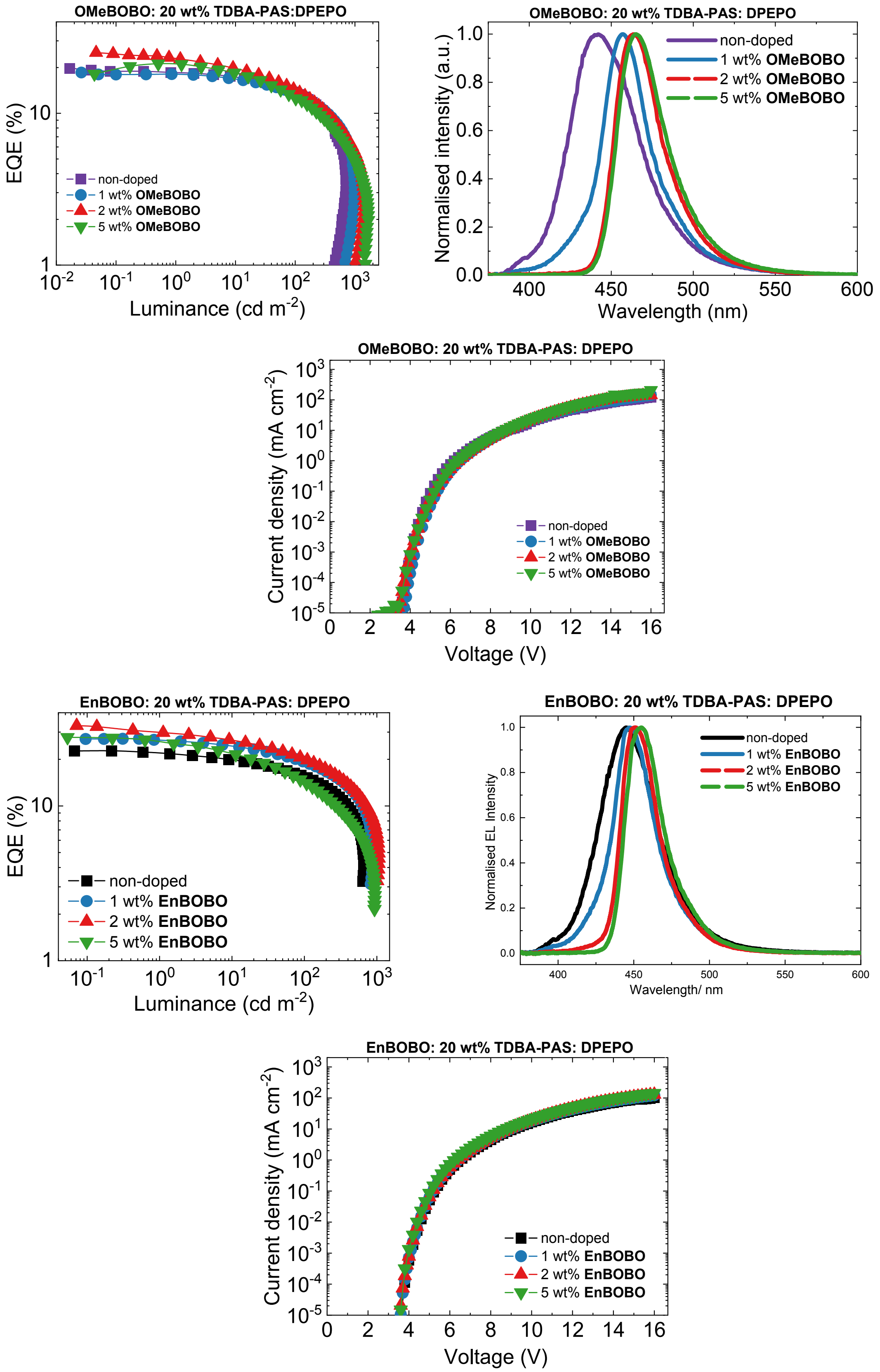

Figure S47. External quantum efficiency roll-off, current-voltage characteristics, and electroluminescence spectra of of the non-doped and doped OLED devices.

Table S17. Key photophysical and device performance parameters of **EnBOBO** versus other high performance reported blue MR-TADF emitters. EQEmax > 30%, FWHM < 25 nm, and CIEy ≤ 0.5 are highlighted with green. *Hyperfluorescent device. Note: some reported lifetimes relate to the solution-state, while others relate to a film.

Emitter	τ_p/ ns	τ_d/ μs	EQE_{max}/%	λ_{EL}/ nm	FWHM/ nm	CIExy
**ν-DABNA Analogues**						
EnBOBO*	5.3	12	33.0	451	28	(0.15, 0.05)
ν-DABNA[22]	4.1	4.1	34.4	469	18	(0.12, 0.11)
t-Bu-ν-DABNA*[23]	5.8	1.6	36.2	474	19	(0.13, 0.19)
BOBO-Z[24]	3.2	16	13.6	445	18	(0.15, 0.04)
BOBS-Z[24]	1.3	18	26.9	456	23	(0.14, 0.06)
BSBS-Z[24]	1.0	15	26.8	463	22	(0.13, 0.08)
ν-DABNA-O-Me[25]	5.1	7.7	29.5	465	23	(0.13, 0.10)
4F-ν-DABNA[26]	8.7	3.1	35.8	464	18	(0.13, 0.10)
4F-m-ν-DABNA[26]	8.2	3.2	33.7	461	18	(0.13, 0.06)
DMBNO*[27]	3.4	41	36.8	450	33	(0.15, 0.07)
t-BuCz-DABNA*[28]	4.1	4.3	29.9	472	17	(0.14, 0.19)
o-Tol-ν-DABNA-Me*[29]	4.6	1.7	35.4	472	18	(0.12, 0.15)
NO-DBMR[30]	2.0	1.4	33.7	469	26	(0.12, 0.12)
**Other MR-TADFs**						
t-DABNA*[31]	6.0	83	31.4	462	31	(0.13, 0.15)
Me-t-DABNA*[32]	8.0	72	32.5	457	22	(0.14, 0.12)
CzBN	4.7	102	27.8	487	26	-
BBCz-DB[31]	10.5	38	29.8	476	36	(0.11, 0.22)
tBu-Cz-DABNA*[32]	7.5	42	28.5	464	27	(0.13, 0.13)
Tp-DABNA*[33]	4.9	200	27.5	462	29	(0.14, 0.13)
TPD4PA[34]	7.8	4.7	30.7	455	29	(0.14, 0.06)
tBu-TPD4PA[34]	8.1	5.6	32.5	460	29	(0.14, 0.07)
BnCz-NPO*[35]	4.8	14	37.6	480	26	(0.11, 0.18)
BnCz-NPS*[35]	4.7	9.7	32.2	476	30	(0.13, 0.18)
TBDON[36]	4.0	36	28.1	471	30	(0.12, 0.17)
DOB2-DABNA-A[37]	6.2	1.6	24.1	452	24	(0.15, 0.05)
DOB2-DABNA-B-NP[37]	7.5	3.0	29.1	471	23	(0.12, 0.13)
BN1*[38]	4.3	130	31.2	456	28	(0.14, 0.08)
BN2*[38]	3.4	75	33.2	468	23	(0.13, 0.11)
BN3*[38]	1.3	18	37.6	457	23	(0.14, 0.08)

Supporting References

(1) Rodriguez Moreno, M.; Setelin, M. L.; Hansen, J. D.; Corey, J. L.; Noble, K. L.; Stillwell, L. R.; Angell, E.; Stubbs, O. A.; Kumawat, J.; Muñoz Gomez, C. S.; Smith, S. J.; Ess, D. H.; Michaelis, D. J. Controlling catalyst speciation to achieve room-temperature Pd-catalyzed aminations with aryl and heteroaryl chlorides. *Adv. Synth. Catal.* **2025**, *367*, 8. https://doi.org/10.1002/adsc.202401337.

(2) Yamamoto, Y.; Moritani, I. Carbon-13 nuclear magnetic resonance studies of organoboranes. Relative importance of mesomeric boron–carbon π-bonding forms in alkenyl- and alkynylboranes. *J. Org. Chem.* **1975**, *40*, 3434–3437. https://doi.org/10.1021/jo00911a029.

(3) Cho, H.H.; Congrave, D. G.; Gillett, A. J.; Montanaro, S.; Francis, H. E.; Riesgo-Gonzalez, V.; Ye, J.; Chowdury, R.; Zeng, W.; Etherington, M. K.; Royakkers, J.; Millington, O.; Bond, A. D.; Plasser, F.; Frost, J. M.; Grey, C. P.; Rao, A.; Friend, R. H.; Greenham, N. C.; Bronstein, H. Suppression of Dexter transfer by covalent encapsulation for efficient matrix-free narrowband deep-blue hyperfluorescent OLEDs. *Nat. Mater.* **2024**, *23*, 519–526. https://doi.org/10.1038/s41563-024-01812-4.

(4) Reck, L. M.; Haberhauer, G.; Lüning, U. Enantiopure chiral concave 1,10-phenanthrolines. *Eur. J. Org. Chem.* **2016**, *2016*, 1119–1131. https://doi.org/10.1002/ejoc.201501289.

(5) Tan, H.; Yang, G.; Deng, Y.; Cao, C.; Tan, J.; Zhu, Z.; Chen, W.; Xiong, Y.; Jian, J.; Lee, C.; Tong, Q. Deep-blue OLEDs with Rec.2020 blue gamut compliance and EQE over 22% achieved by conformation engineering. *Adv. Mater.* **2022**, *34*, 2200537. https://doi.org/10.1002/adma.202200537.

(6) Tan, H.J.; Yu, J.R.; Lin, Z.Z.; Yang, G.X.; Long, Z.Q.; Deng, Y.L.; Zhu, Z.L.; Chen, X.K.; Jian, J.X.; Tong, Q.X.; Lee, C.S. The role of a small molecular dipole moment for efficient non-doped deep-blue thermally activated delayed fluorescence emitters. *Chem. Eng. J.* **2024**, *481*, 148567. https://doi.org/10.1016/j.cej.2024.148567.

(7) Sheldrick, G. M. SHELXT – integrated space-group and crystal-structure determination. *Acta Crystallogr. A* **2015**, *71*, 3–8. https://doi.org/10.1107/S2053273314026370.

(8) Sheldrick, G. M. Crystal structure refinement with SHELXL. *Acta Crystallogr. C* **2015**, *71*, 3–8. https://doi.org/10.1107/S2053229614024218.

(9) Spek, A. L. PLATON SQUEEZE: a tool for the calculation of the disordered solvent contribution to the calculated structure factors. *Acta Crystallogr. C* **2015**, *71*, 9–18. https://doi.org/10.1107/S2053229614024929.

(10) Ahn, T.-S.; Al-Kaysi, R. O.; Müller, A. M.; Wentz, K. M.; Bardeen, C. J. Self-absorption correction for solid-state photoluminescence quantum yields obtained from integrating sphere measurements. *Rev. Sci. Instrum.* **2007**, *78*, 083102. https://doi.org/10.1063/1.2768926.

(11) Masui, K.; Nakanotani, H.; Adachi, C. Analysis of exciton annihilation in high-efficiency sky-blue organic light-emitting diodes with thermally activated delayed fluorescence. *Org. Electron.* **2013**, *14*, 2721–2726. https://doi.org/10.1016/j.orgel.2013.07.010.

(12) Frisch, M. J.; Trucks, G. W.; Schlegel, H. B.; Scuseria, G. E.; Robb, M. A.; Cheeseman, J. R.; Scalmani, G.; Barone, V.; Petersson, G. A.; Nakatsuji, H.; Li, X.; Caricato, M.; Marenich, A. V.; Bloino, J.; Janesko, B. G.; Gomperts, R.; Mennucci, B.; Hratchian, H. P.; Ortiz, J. V.; Izmaylov, A. F.; Sonnenberg, J. L.; Williams-Young, D.; Ding, F.; Lipparini, F.; Egidi, F.; Goings, J.; Peng, B.; Petrone, A.; Henderson, T.; Ranasinghe, D.; Zakrzewski, V. G.; Gao, J.; Rega, N.; Zheng, G.; Liang, W.; Hada, M.; Ehara, M.; Toyota, K.; Fukuda, R.; Hasegawa, J.; Ishida, M.; Nakajima, T.; Honda, Y.; Kitao, O.; Nakai, H.; Vreven, T.; Throssell, K.; Montgomery, J. A. Gaussian 16, Revision A.03; Gaussian, Inc.: Wallingford, CT, 2016.

(13) Lee, C.; Yang, W.; Parr, R. G. Development of the Colle-Salvetti correlation-energy formula into a functional of the electron density. *Phys. Rev. B* **1988**, *37*, 785–789. https://doi.org/10.1103/PhysRevB.37.785.

(14) Becke, A. D. Density-functional thermochemistry. III. The role of exact exchange. *J. Chem. Phys.* **1993**, *98*, 5648–5652. https://doi.org/10.1063/1.464913.

(15) Petersson, G. A.; Al-Laham, M. A. A complete basis set model chemistry. II. Open-shell systems and the total energies of the first-row atoms. *J. Chem. Phys.* **1991**, *94*, 6081–6090. https://doi.org/10.1063/1.460447.

(16) Petersson, G. A.; Bennett, A.; Tensfeldt, T. G.; Al-Laham, M. A.; Shirley, W. A.; Mantzaris, J. A complete basis set model chemistry. I. The total energies of closed-shell atoms and hydrides of the first-row elements. *J. Chem. Phys.* **1988**, *89*, 2193–2218. https://doi.org/10.1063/1.455064.

(17) Grimme, S.; Antony, J.; Ehrlich, S.; Krieg, H. A consistent and accurate ab initio parametrization of density functional dispersion correction (DFT-D) for the 94 elements H–Pu. *J. Chem. Phys.* **2010**, *132*, 154104. https://doi.org/10.1063/1.3382344.

(18) Lu, T.; Chen, F. Multiwfn: a multifunctional wavefunction analyzer. *J. Comput. Chem.* **2012**, *33*, 580–592. https://doi.org/10.1002/jcc.22885.

(19) Liu, Z.; Lu, T.; Chen, Q. An sp-hybridized all-carboatomic ring, cyclo[18]carbon: electronic structure, electronic spectrum, and optical nonlinearity. *Carbon* **2020**, *165*, 461–467. https://doi.org/10.1016/j.carbon.2020.05.023.

(20) Le Bahers, T.; Adamo, C.; Ciofini, I. A qualitative index of spatial extent in charge-transfer excitations. *J. Chem. Theory Comput.* **2011**, *7*, 2498–2506. https://doi.org/10.1021/ct200308m.

(21) Humphrey, W.; Dalke, A.; Schulten, K. VMD: visual molecular dynamics. *J. Mol. Graph.* **1996**, *14*, 33–38. https://doi.org/10.1016/0263-7855(96)00018-5.

(22) Kondo, Y.; Yoshiura, K.; Kitera, S.; Nishi, H.; Oda, S.; Gotoh, H.; Sasada, Y.; Yanai, M.; Hatakeyama, T. Narrowband Deep-Blue Organic Light-Emitting Diode Featuring an Organoboron-Based Emitter. *Nat. Photon.* **2019**, *13*, 678–682. https://doi.org/10.1038/s41566-019-0476-5.

(23) Naveen, K. R.; Lee, H.; Braveenth, R.; Karthik, D.; Yang, K. J.; Hwang, S. J.; Kwon, J. H. Achieving High Efficiency and Pure Blue Color in Hyperfluorescence Organic Light Emitting Diodes Using Organo-Boron Based Emitters. *Adv. Funct. Mater.* **2022**, *32*. https://doi.org/10.1002/adfm.202110356 .

(24) Park, I. S.; Yang, M.; Shibata, H.; Amanokura, N.; Yasuda, T. Achieving Ultimate Narrowband and Ultrapure Blue Organic Light-Emitting Diodes Based on Polycyclo-Heteraborin Multi-Resonance Delayed-Fluorescence Emitters. *Adv. Mater.* **2022**, *34* https://doi.org/10.1002/adma.202107951 .

(25) Tanaka, H.; Oda, S.; Ricci, G.; Gotoh, H.; Tabata, K.; Kawasumi, R.; Beljonne, D.; Olivier, Y.; Hatakeyama, T. Hypsochromic Shift of Multiple-Resonance-Induced Thermally Activated Delayed Fluorescence by Oxygen Atom Incorporation. *Angew. Chem.* **2021**, *133*, 18054–18058. https://doi.org/10.1002/ange.202105032 .

(26) Rayappa Naveen, K.; Lee, H.; Braveenth, R.; Joon Yang, K.; Jae Hwang, S.; Hyuk Kwon, J. Deep Blue Diboron Embedded Multi-Resonance Thermally Activated Delayed Fluorescence Emitters for Narrowband Organic Light Emitting Diodes. *Chem. Eng. J.* **2022**, *432*, 134381. https://doi.org/10.1016/j.cej.2021.134381 .

(27) Xue, Z.; Xiao, Z.; Zou, Y.; Chen, Z.; Liu, J.; Huang, Z.; Yang, C. A Mesityl-Functionalized Double-Boron–Nitrogen–Oxygen-Embedded Multi-Resonance Framework Achieves Anti-Quenching Narrowband Deep-Blue Electroluminescence with EQE over 30% and CIE $_y$ of 0.046. *Chem. Sci.* **2025**, *16*, 3655–3661. https://doi.org/10.1039/D4SC07503J .

(28) Zhang, K.; Wang, X.; Chang, Y.; Wu, Y.; Wang, S.; Wang, L. Carbazole-Decorated Organoboron Emitters with Low-Lying HOMO Levels for Solution-Processed Narrowband Blue Hyperfluorescence OLED Devices. *Angew. Chem. Int. Ed.* **2023**, *62*. https://doi.org/10.1002/anie.202313084 .

(29) Kim, H. S.; Cheon, H. J.; Lee, D.; Lee, W.; Kim, J.; Kim, Y.-H.; Yoo, S. Toward Highly Efficient Deep-Blue OLEDs: Tailoring the Multiresonance-Induced TADF Molecules for Suppressed Excimer Formation and near-Unity Horizontal Dipole Ratio. *Sci. Adv.* **2023**, *9*. https://doi.org/10.1126/sciadv.adf1388 .

(30) Naveen, K. R.; Oh, J. H.; Lee, H. S.; Kwon, J. H. Tailoring Extremely Narrow FWHM in Hypsochromic and Bathochromic Shift of Polycyclo-Heteraborin MR-TADF Materials for High-Performance OLEDs. *Angew. Chem. Int. Ed.* **2023**, *62*. https://doi.org/10.1002/anie.202306768 .

(31) Han, S. H.; Jeong, J. H.; Yoo, J. W.; Lee, J. Y. Ideal Blue Thermally Activated Delayed Fluorescence Emission Assisted by a Thermally Activated Delayed Fluorescence Assistant Dopant through a Fast Reverse Intersystem Crossing Mediated Cascade Energy Transfer Process. *J. Mater. Chem. C* **2019**, *7*, 3082–3089. https://doi.org/10.1039/C8TC06575F .

(32) Hou, L.; Wang, J.; Nie, Y.; Guo, K.; Hu, L.; Wang, Y.; Peng, J. Enhancing Device Efficiency Through Subtle Substituent Tuning in DABNA-Based Emitters. *FlexTech* **2025**, *1*, 115–122. https://doi.org/10.1002/fle2.70005 .

(33) Yang, M.; Park, I. S.; Yasuda, T. Full-Color, Narrowband, and High-Efficiency Electroluminescence from Boron and Carbazole Embedded Polycyclic Heteroaromatics. *J. Am. Chem. Soc.* **2020**, *142*, 19468–19472. https://doi.org/10.1021/jacs.0c10081 .

(34) Guo, L.; Cui, W.; Li, L.; Pu, Y.; Wang, K.; Zheng, P.; Wang, Y.; Li, C. Synergetic Multiple Charge-Transfer Excited States for Anti-Quenching and Rapid Spin-Flip Multi-Resonance Thermally Activated Delayed Fluorescence Emitter. *Adv. Mater.* **2025**, *37*. https://doi.org/10.1002/adma.202500269 .

(35) Tirupati, M.; Ham, J. H.; Muruganantham, S.; Cha, S. C.; Jung, Y. H.; Kwon, J. H. Toward Narrow-Band, Stable Blue MR-TADF Emission via Combined Heterocyclic Analogues Marching B.T.2020. *Angew. Chem. Int. Ed.* **2025**, *64*. https://doi.org/10.1002/anie.202510190 .

(36) Mubarok, H.; Amin, A.; Lee, T.; Jung, J.; Lee, J.; Lee, M. H. Triptycene-Fused Sterically Shielded Multi-Resonance TADF Emitter Enables High-Efficiency Deep Blue OLEDs with Reduced Dexter Energy Transfer. *Angew. Chem. Int. Ed.* **2023**, *62*. https://doi.org/10.1002/anie.202306879 .

(37) Naveen, K. R.; Lee, H.; Seung, L. H.; Jung, Y. H.; Keshavananda Prabhu, C. P.; Muruganantham, S.; Kwon, J. H. Modular Design for Constructing Narrowband Deep-Blue Multiresonant Thermally Activated Delayed Fluorescent Emitters for Efficient Organic Light Emitting Diodes. *Chem. Eng. J.* **2023**, *451*, 138498. https://doi.org/10.1016/j.cej.2022.138498 .

(38) Xing, L.; Wang, J.; Chen, W.-C.; Liu, B.; Chen, G.; Wang, X.; Tan, J.-H.; Chen, S. S.; Chen, J.-X.; Ji, S.; Zhao, Z.; Tang, M.-C.; Huo, Y. Highly Efficient Pure-Blue Organic Light-Emitting Diodes Based on Rationally Designed Heterocyclic Phenophosphazinine-Containing Emitters. *Nat. Commun.* **2024**, *15*, 6175. https://doi.org/10.1038/s41467-024-50370-5 .

(39) Wu, S.; Chen, D.; Seinfeld, M.; McKay, A. P.; Cordes, D. B.; Zhang, X.; Zysman-Colman, E. A Three Boron Doped B/O/N Multi-Resonant TADF Emitter for Improved Reverse Intersystem Crossing Rate and Efficient Pure Blue Organic Light-Emitting Diodes. *Chem. Sci.* **2025**, *16*, 15256–15264. https://doi.org/10.1039/D5SC03560K .

(40) Ochi, J.; Yamasaki, Y.; Tanaka, K.; Kondo, Y.; Isayama, K.; Oda, S.; Kondo, M.; Hatakeyama, T. Highly Efficient Multi-Resonance Thermally Activated Delayed Fluorescence Material toward a BT.2020 Deep-Blue Emitter. *Nat. Commun.* **2024**, *15*, 2361. https://doi.org/10.1038/s41467-024-46619-8 .

(41) Lv, X.; Miao, J.; Liu, M.; Peng, Q.; Zhong, C.; Hu, Y.; Cao, X.; Wu, H.; Yang, Y.; Zhou, C.; Ma, J.; Zou, Y.; Yang, C. Extending the Π-Skeleton of Multi-Resonance TADF Materials towards High-Efficiency Narrowband Deep-Blue Emission. *Angew. Chem. Int. Ed.* **2022**, *61*. https://doi.org/10.1002/anie.202201588 .
